# Synthesis of Aryl‐Substituted Hexa‐Alkynyl Hexaazatrinaphthylenes via Sonogashira Coupling and Evaluation of Their Photophysical Properties

**DOI:** 10.1002/asia.202501003

**Published:** 2026-02-16

**Authors:** Yuchen Wu, Chisae Kumagai, Natsuhiko Sugimura, Takanori Shibata

**Affiliations:** ^1^ Department of Chemistry and Biochemistry School of Advanced Science and Engineering Waseda University Tokyo Japan; ^2^ Materials Characterization Central Laboratory Waseda University Tokyo Japan

**Keywords:** heterocycles, hexaazatrinaphthylene, polycyclic aromatic compounds, Sonogashira coupling

## Abstract

Hexaazatrinaphthylene (HATNA) has recently been a subject of great interest as a polycyclic heteroaromatic scaffold in materials science, specifically in the field of discotic liquid crystals (DLCs), battery cathodes, n‐type semiconductors, and so on. However, little is known about the photophysical properties of HATNA derivatives. Here we report the synthesis of a new class of hexa(arylethynyl) HATNA derivatives via consecutive Sonogashira coupling. This protocol showed good functional‐group tolerance and moderate to good yields were achieved. The obtained HATNA products showed favorable photophysical properties, with molar absorption coefficients up to 1.69 × 10^5^ (M^−1^ cm^−1^) and high quantum yields up to 0.73.

## Graphical abstract

A new series of hexaalkynyl‐substituted hexaazatrinaphthylene (HATNA) derivatives was successfully synthesized via Sonogashira coupling between hexabromo‐HATNA and aryl‐substituted terminal alkynes. This protocol exhibited good functional‐group tolerance, affording the desired products in moderate to good yields. The resulting HATNA derivatives were confirmed to possess favorable photophysical properties, including high molar absorption coefficients and quantum yields of up to 0.73.

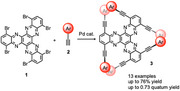

## 1 Introduction

Polycyclic aromatic compounds have attracted widespread interest due to their unique optical, electrical, and magnetic properties, which arise from their highly conjugated π‐systems. Among them, those with *C*_3_‐symmetric geometries generally exhibit favorable physicochemical characteristics and have been widely used in electronic devices as well as in the synthesis of bioactive and pharmaceutical compounds [1, 2, 3, 4, 5].

As a representative example, hexaazatrinaphthylene (HATNA) features a fully conjugated planar π‐system composed of three fused naphthalene units arranged around a central benzene core with six imine‐type nitrogen atoms symmetrically embedded. This configuration forms a rigid *C*_3_‐symmetric framework with extensive electron delocalization and a strong propensity for intermolecular π–π stacking. The highly conjugated planar structure endows HATNA with strong electron‐accepting ability and pronounced self‐assembly, giving its derivatives excellent optoelectronic properties, enhanced polymerization, and the formation of metal–organic frameworks (MOFs) and covalent organic frameworks (COFs) [6, 7, 8, 9]. In recent decades, extensive research has focused on HATNA derivatives as versatile building blocks in diverse applications, including n‐type semiconductors [10, 11], discotic liquid crystals (DLCs) [12, 13, 14], organic electrode materials [15, 16, 17], organogels [18], and photochemical hydrogen storage [19]. However, most of the reported works have concentrated on the electrical properties of HATNA or the formation of porous polymer structures, and little is known about its photophysical properties in the visible light range.

**SCHEME 1:**
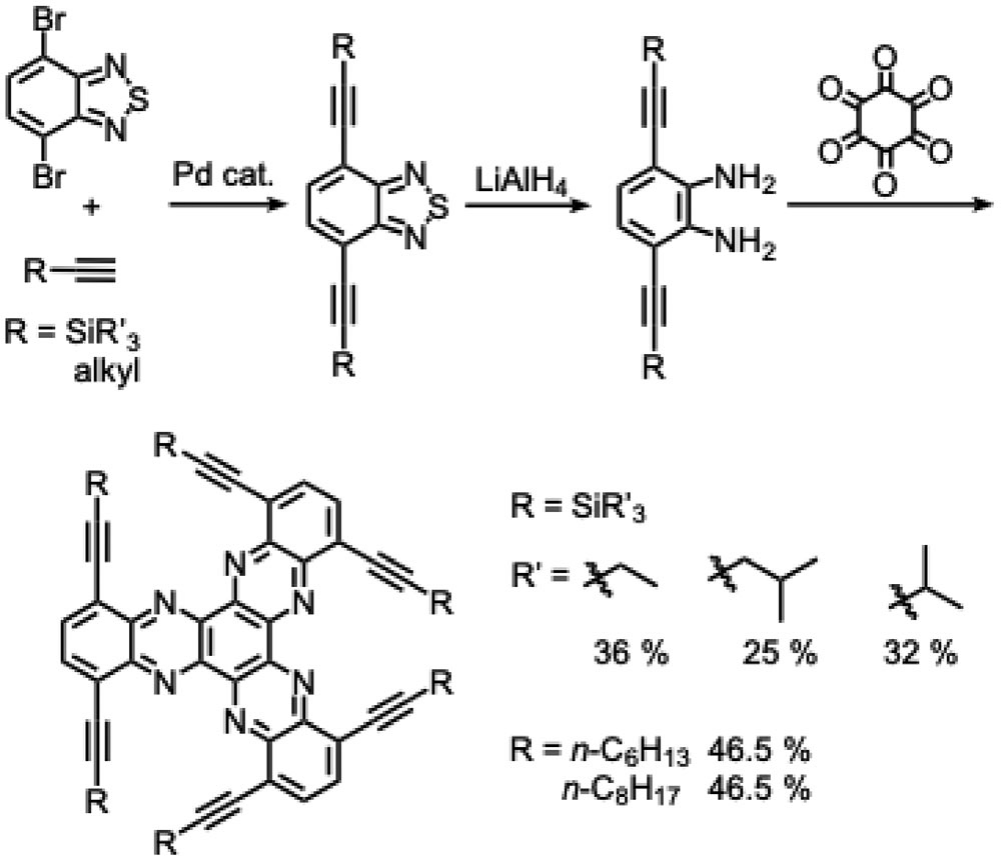
Hexa‐alkynyl‐substituted HATNAs at their bay positions.

Since HATNA offers numerous advantages in materials science applications, functionalization of the HATNA skeleton has attracted considerable attention [20, 21, 22]. Meanwhile, extensive research has demonstrated that twisted structures in polycyclic aromatic compounds can significantly influence their electronic and chiroptical properties [23, 24, 25, 26]. Consequently, twisted HATNA derivatives have aroused growing interest, and can be obtained through backbone functionalization—for instance, by introducing rigid alkynyl substituents at the bay positions. In 2014, Mateo‐Alonso and co‐workers reported the synthesis of HATNAs with varying degrees of twist by incorporating rigid acetylenes with bulky silyl substituents (R = SiR_3_ in Scheme 1) [27]. By tuning the size of the terminal silyl groups, the twist angles of HATNA could be significantly altered, resulting in shifted emission wavelengths and changes in the redox potentials of the twisted HATNAs. In the same year, Liu's group disclosed the synthesis of alkynylated HATNA derivatives with linear alkyl chains at the alkyne termini, which exhibited self‐directed molecular column orientation (R = alkyl in Scheme 1) [28]. However, in these reported studies, only a few examples were obtained in low to moderate yields, and no remarkable photophysical properties were described.

**SCHEME 2:**
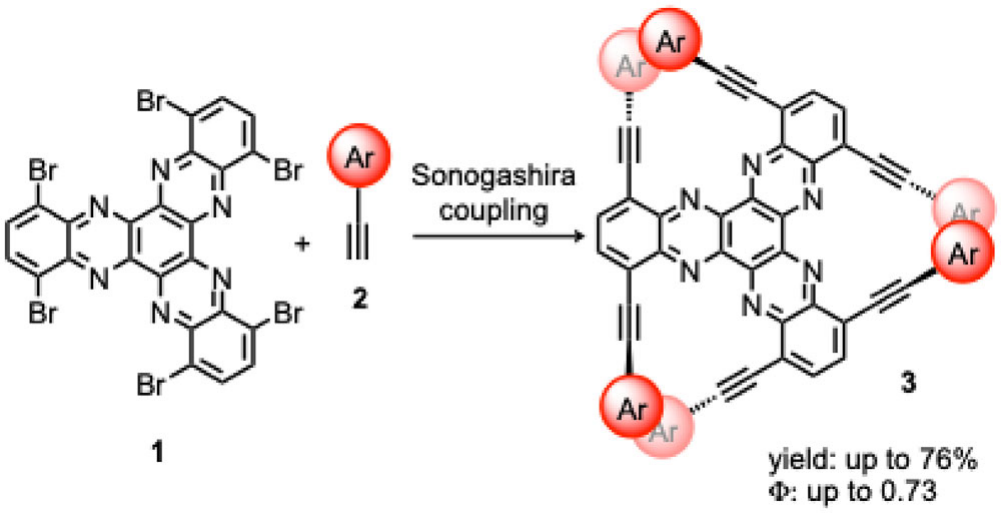
Synthetic protocol of new HATNA derivatives.

Herein, we report a new synthetic protocol for a new series of bay‐substituted hexa‐arylethynyl HATNA derivatives **3**, obtained by consecutive Sonogashira coupling of hexabromo‐HATNA **1** with arylalkyne **2**. This protocol exhibited good functional‐group tolerance, affording hexa‐arylethynyl HATNAs in moderate to good yields. By introducing aryl groups at the alkyne terminals, the resulting HATNA derivatives displayed favorable photophysical properties and achieved high quantum yields of up to 0.73 (Scheme 2).

## 2 Results and Discussion

First, we optimized the reaction conditions using hexabromo‐HATNA **1** and *p*‐tolylacetylene **2a** (Table 1). Conventional conditions for Sonogashira coupling were initially examined, affording the target compound **3a** in 41% yield (entry 1). When the reaction was conducted without PPh_3_, the yield significantly decreased (entry 2). In addition to THF, several other solvents were tested, and DMSO proved to be the most effective (entries 3–5). The reaction was then performed at different temperatures: 100°C gave the best results (entries 5–8). Replacement of diisopropylamine with triethylamine or *N,N,N′,N′*‐tetramethylethylenediamine (TMEDA) afforded **3a** in only moderate yields (entries 9–10). The use of inorganic bases instead of amines proved ineffective (entry 11). Therefore, we determined that Entry 6 represented the optimal reaction conditions.

**TABLE 1:** Optimization of Reaction Conditions^a^.

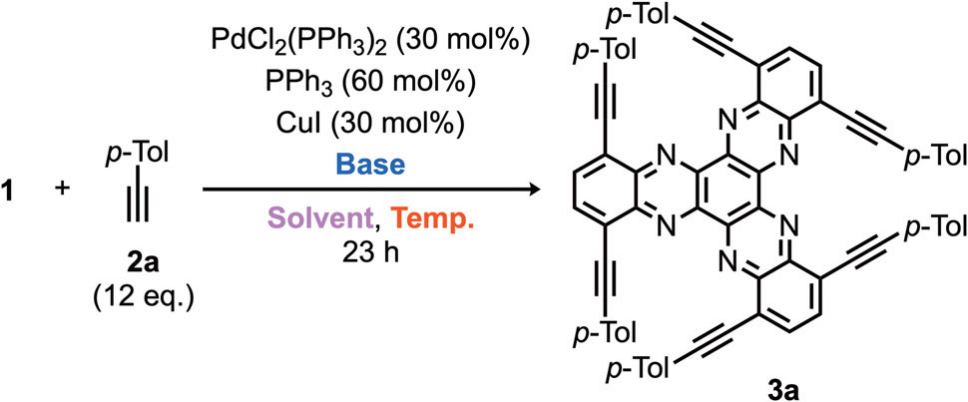				
Entry	Base	Solvent	Temp./ °C	NMR Yield / %
1	*i*‐Pr_2_NH	THF	60	41
2^b^	*i*‐Pr_2_NH	THF	60	20
3	*i*‐Pr_2_NH	1,4‐Dioxane	60	28
4	*i*‐Pr_2_NH	DMF	60	55
5	*i*‐Pr_2_NH	DMSO	60	63
6	*i*‐Pr_2_NH	DMSO	80	81(70^c^)
7	*i*‐Pr_2_NH	DMSO	100	58
8	*i*‐Pr_2_NH	DMSO	140	2
9	Et_3_N	DMSO	80	50
10	TMEDA	DMSO	80	47
11^d^	Cs_2_CO_3_	DMSO	80	53

^a^ Unless otherwise noted, the reaction was conducted at 25 µmol scale in solvent (1.5 mL), amine (1.5 mL) under argon. The NMR yield was determined using 1,1,2,2‐tetrachloroethane as an internal standard.

^b^ Reaction was conducted without the addition of PPh_3_.

^c^ Isolated yield.

^d^ Twelve equivalent amounts of Cs_2_CO_3_ were used instead of amine.

Next, under the optimal conditions, we examined a variety of aryl acetylenes (Table 2). The use of phenyl acetylene **2b** as the coupling partner afforded **3b** in moderate yield. Substituent variation at the *para*‐position from methyl (**3a**) to electron‐donating (**3c**), electron‐withdrawing (**3e**, **3f**), and bulky groups (**3d**) was generally well tolerated. Only in the case of the cyano group was a pronounced decrease in yield (**3f**). In addition to monosubstituted aryl acetylenes, derivatives bearing multiple substituents at the *para*‐ and *meta*‐positions also furnished the desired products **3g** and **3h** in high yields. In contrast, substitution at the *ortho*‐position exerted a clear negative effect on the reaction outcome (**3i**). Moreover, bulkier aryl acetylenes such as naphthyl and 1‐pyrenyl derivatives were well accommodated, giving products **3j–3l** in good yields. Heteroaryl acetylene was also compatible with this protocol, affording the corresponding product **3m** in moderate yield. We also evaluated the solubility of the obtained compounds using **3a** as a representative example. Compound **3a** showed good solubility in several common organic solvents: 1 mg dissolved in approximately 0.1 mL of dichloromethane (DCM), ∼1.5 mL of THF, and ∼3 mL of DMF. In contrast, its solubility in DMSO and acetone was significantly low, requiring more than 20 mL of solvent to dissolve 1 mg of **3a**. Moreover, **3a** was practically insoluble in alcohols such as methanol and ethanol.

**TABLE 2:** Substrate Scope^a^ ^.^

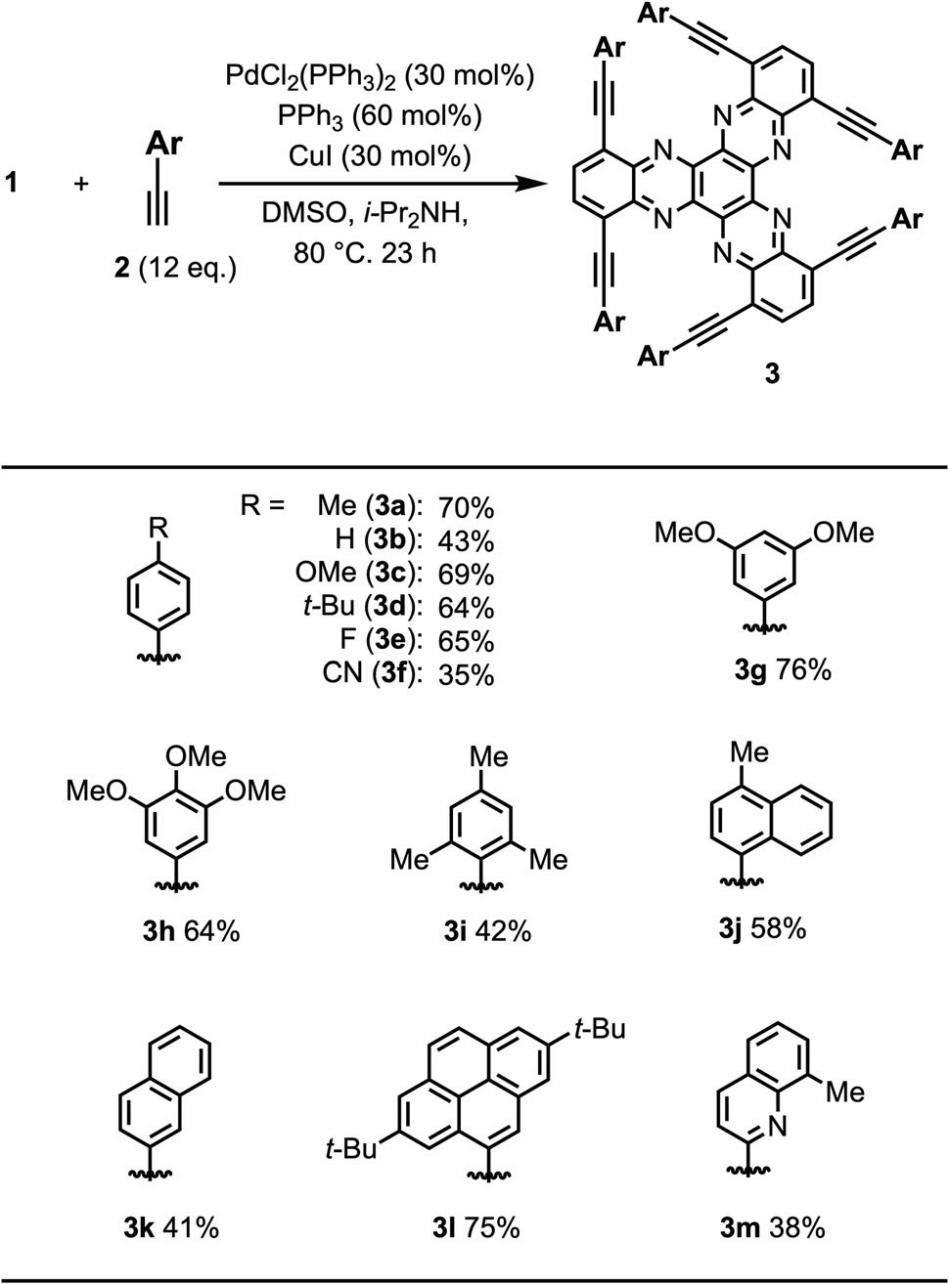

^a^ The reaction was conducted at 25 µmol scale in DMSO (1.5 mL), diisopropylamine (1.5 mL) under argon.

**FIGURE 1:**
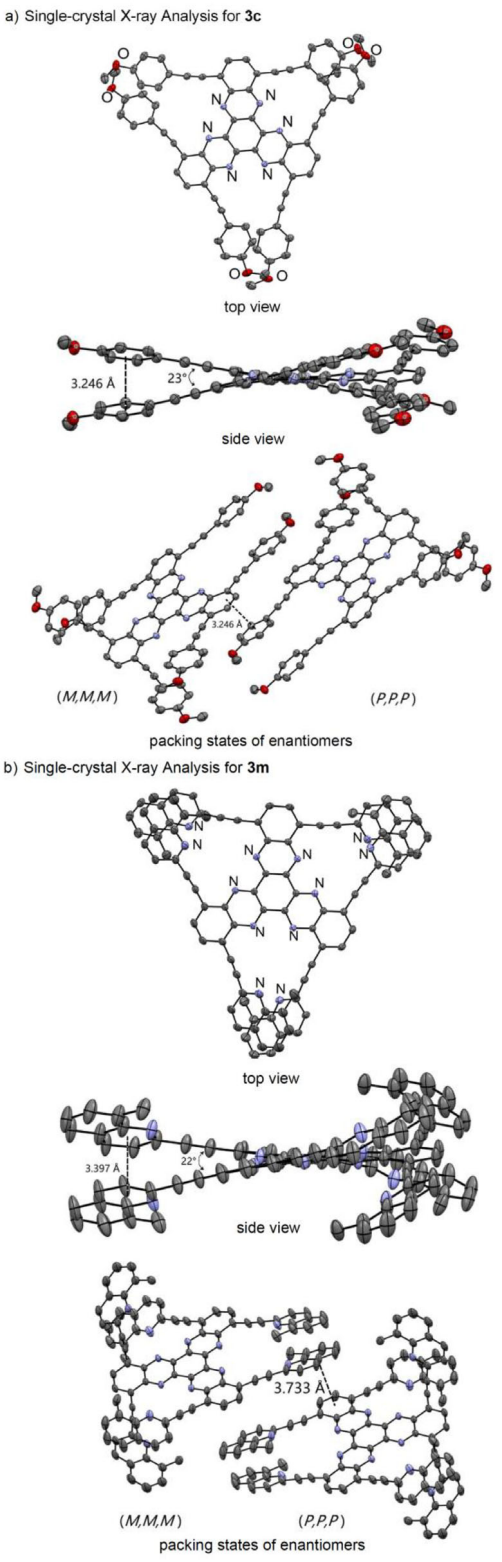
ORTEP Diagrams of **3c** and **3m** (thermal ellipsoids are shown at 50% probability level, hydrogen atoms are omitted for clarity).

Among the newly obtained hexa‐alkynyl HATNA derivatives, **3c** and **3m** were suitable for single‐crystal x‐ray structure analysis (Figure 1) [29]. Overlap between the terminal aryl groups of adjacent blades was clearly observed. The inter‐blade distances were measured to be 3.246 Å for **3c** and 3.397 Å for **3m**, indicating the presence of π–π stacking interactions between the aromatic groups, with only a slight influence from substituent bulkiness. From the side‐views, the twist angles between the blades were measured to be 23° for **3c** and 22° for **3m**, with almost no difference. Examination of the intermolecular packing revealed the presence of both (*P,P,P*) and (*M,M,M*) enantiomeric pairs in the crystals of **3c** and **3m**. These enantiomers were arranged in an orderly manner, with the terminal aryl groups of one enantiomer engaging in π–π stacking interactions with the benzene ring at the periphery of another HATNA skeleton. In **3c**, the distance between the interacting units was 3.246 Å, whereas in **3m** it was 3.733 Å, highlighting the influence of substituent bulkiness on intermolecular π–π stacking. Since enantiomeric pairs were observed in the single‐crystal structures, high‐performance liquid chromatography (HPLC) analysis was attempted using various chiral columns provided by Daicel Corporation. However, the chirality could not be confirmed, possibly due to the flipping of molecules in solution at room temperature [30].

**FIGURE 2:**
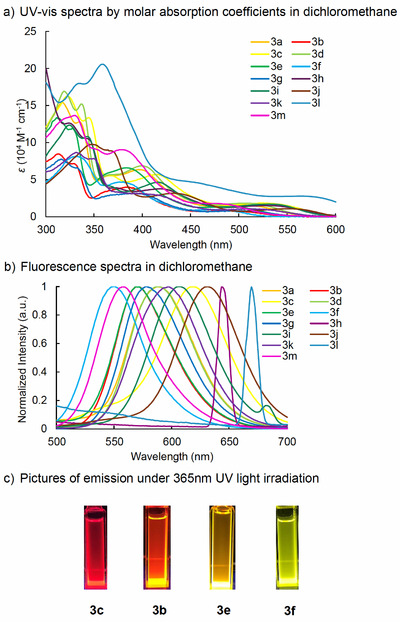
UV–vis and Fluorescence Spectra of Hexaalkynyl HATNAs.

The photophysical properties of the obtained hexa‐alkynyl HATNA derivatives were measured in dichloromethane, and the results are summarized in Table 3 and Figure 2. The maximum molar absorption coefficients (*ε*) were found to depend strongly on the substituents at the aryl groups on the alkyne termini. Derivatives bearing electron‐donating substituents exhibited higher *ε* values than those with electron‐withdrawing groups, while bulky aryl substituents led to a decrease in *ε* [31]. All samples displayed absorption bands in the long‐wavelength region (450–550 nm) (Figure 2a). The maximum emission wavelengths also showed a strong dependence on the electron density of the aryl groups, with electron‐withdrawing substituents inducing significant hypsochromic shifts (Figure 2b). Representative emission photographs of derivatives with *para*‐methoxy (**3c**), *para*‐methyl (**3b**), *para*‐fluoro (**3e**), and *para*‐cyano groups (**3f**) under 365 nm UV irradiation revealed distinct color changes from red to yellow (Figure 2c). Remarkably, all the obtained HATNA derivatives exhibit very large Stokes shifts, exceeding 200 nm. Besides dichloromethane, we also examined the absorption and emission behaviors in THF solution, where only a slight decrease in *ε* and a small hypsochromic shift of the absorption and emission maximum were observed (Figure S1 and Table S1). In addition, fluorescence quantum yields were measured for all samples in dichloromethane (DCM) solution and solid state (Table 4). Most derivatives exhibited moderate to high values in solution, with the highest reaching 0.73, markedly surpassing previously reported HATNA derivatives with precisely determined quantum yields [32]. Some of the samples also exhibited solid‐state fluorescence, with quantum yields of up to 0.22, which we attribute to aggregation enabled by intermolecular π–π stacking interactions. In general, HATNA derivatives bearing electron‐deficient substituents exhibited higher quantum yields than those with electron‐rich groups, while the quantum yield of the trimethoxyphenyl‐substituted HATNA **3h** decreased dramatically. Notably, the pyrene‐substituted derivative **3l** displayed the largest redshift, with a maximum emission wavelength of 669 nm. However, the quantum yield of **3l** was lower than that of other HATNA derivatives. This reduction may be attributed to the bulkiness of the aryl substituent, which could hinder intramolecular π–π stacking interactions—believed to play a significant role in the favorable fluorescence properties of these new HATNA derivatives.

**TABLE 3:** Photophysical Properties of Hexa‐alkynyl HATNAs **3**.

Comp.	*λ*_max(abs)_ (nm) (*ε* [× 10^4^ M^−1^ cm^−1^])^a^	λ_max(em)_ (nm) ^b^, ^c^	Stokes Shift (nm)
**3a**	317(15.4), 335(12.8), 396(6.4), 500(1.6)	587	270
**3b**	313(8.5), 328(7.2), 383(4.0), 485(0.79)	567	254
**3c**	322(16.3), 344(13.4), 415(5.7), 525(1.9)	619	297
**3d**	319(16.9), 336 (15.2), 399(6.9), 506(1.8)	585	266
**3e**	311(11.4), 327(12.0), 383(6.6), 487(1.3)	569	258
**3f**	331(8.1), 376(4.7), 478(0.97)	547	216
**3g**	315(7.8), 332(sh), 391(3.2), 498(0.81)	575	260
**3h**	323(12.6), 418(3.8), 530(1.9)	643	320
**3i**	324(12.4), 342(10.8), 416(4.7), 531(1.7)	606	282
**3j**	347(9.8), 433(3.2), 551(1.1)	589	242
**3k**	331(8.7), 349(7.9), 400(sh), 510(1.0)	595	264
**3l**	334(18.0), 358(20.6)	669	335
**3m**	330 (13.7), 378(9.0), 480(sh)	558	228

^a^ **3a**: 5.60×10^−6 ^M; **3b**: 2.79×10^−6 ^M; **3c**: 5.23×10^−6 ^M; **3d**: 2.86×10^−5 ^M; **3e**: 2.85×10^−5 ^M; **3f**: 2.47×10^−6 ^M; **3g**: 2.23×10^−6 ^M; **3h**: 2.79×10^−6 ^M; **3i**: 2.63×10^−6 ^M; **3j**: 2.60×10^−5 ^M; **3k**: 2.20×10^−6 ^M; **3l**: 2.39×10^−6 ^M; **3m**: 2.84×10^−6 ^M.

^b^ Excitation wavelengths: **3a**: 317 nm; **3b**: 313 nm; **3c**: 322 nm; **3d**: 319 nm; **3e**: 311 nm; **3f**: 331 nm; **3g**: 315 nm; **3h**: 323 nm; **3i**: 342 nm; **3j**: 347 nm; **3k**: 331 nm; **3l**: 334 nm; **3m**: 330 nm.

^c^ **3a**: 5.60×10^−6 ^M; **3b**: 2.79×10^−6 ^M; **3c**: 5.23×10^−6 ^M; **3d**: 2.86×10^−6 ^M; **3e**: 2.85×10^−6 ^M; **3f**: 2.47×10^−6 ^M; **3g**: 2.23×10^−6 ^M; **3h**: 2.79×10^−6 ^M; **3i**: 2.63×10^−6 ^M; **3j**: 2.60×10^−6 ^M; **3k**: 2.20×10^−6 ^M, **3l**: 2.39×10^−6 ^M; **3m**: 2.84×10^−6 ^M.

**TABLE 4:** Fluorescence Quantum Yields of Hexa‐alkynyl HATNAs **3**.

Comp.	Φ in DCM^a^, ^b^	Φ in solid state^b^
**3a**	0.54	0.22
**3b**	0.54	0.08
**3c**	0.20	0.09
**3d**	0.55	0.04
**3e**	0.73	0.11
**3f**	0.65	0.03
**3g**	0.38	0.05
**3h**	0.01	—
**3i**	0.69	—
**3j**	0.28	0.06
**3k**	0.44	0.02
**3l**	0.02	—
**3m**	0.59	0.03

^a^ **3a**: 5.60×10^−6 ^M; **3b**: 2.79×10^−6 ^M; **3c**: 5.23×10^−6 ^M; **3d**: 2.86×10^−5 ^M; **3e**: 2.85×10^−5 ^M; **3f**: 2.47×10^−6 ^M; **3g**: 2.23×10^−6 ^M; **3h**: 2.79×10^−6 ^M; **3i**: 2.63×10^−6 ^M; **3j**: 2.60×10^−5 ^M; **3k**: 2.20×10^−6 ^M; **3l**: 2.39×10^−6 ^M; **3m**: 2.84×10^−6 ^M.

^b^ Excitation wavelengths: **3a**: 317 nm; **3b**: 313 nm; **3c**: 322 nm; **3d**: 319 nm; **3e**: 311 nm; **3f**: 331 nm; **3g**: 315 nm; **3h**: 323 nm; **3i**: 342 nm; **3j**: 347 nm; **3k**: 331 nm; **3l**: 334 nm; **3m**: 330 nm.

## 3 Conclusion

We have developed a synthetic method for a new class of hexaalkynyl HATNA derivatives via Sonogashira coupling, starting from easily prepared hexabromo‐HATNA **1** and commercially available or readily accessible arylalkynes **2**. This protocol exhibited broad functional‐group tolerance and afforded hexa(arylethynyl) HATNAs **3** with diverse substituents in moderate to high yields. Unlike in previous studies on HATNA, the hexa‐arylalkynyl derivatives **3** displayed favorable photophysical properties, achieving moderate to high fluorescent quantum yields in dichloromethane solutions, thereby highlighting their potential in photophysical applications of HATNA‐based materials.

## Conflicts of Interest

The authors have nothing to report.

## Supporting information



Experimental details, analytical data, and NMR spectral copies of substrates and products (PDF).**Supporting File 1**: asia70632‐sup‐0001‐SuppMat.pdf.

**Supporting File 2**: asia70632‐sup‐0002‐DataFile.zip.

## References

[1] M. A. Alshubramy , K. A. Alamry , and M. A. Hussein , “An Overview of the Synthetic Strategies of C 3‐symmetric Polymeric Materials Containing Benzene and Triazine Cores and Their Biomedical Applications,” RSC Advances 13 (2023): 14317–14339, 10.1039/D3RA01336G. 37179987 PMC10170496

[2] J. De , S. P. Gupta , S. S. Swayamprabha , et al., “Blue Luminescent Organic Light Emitting Diode Devices of a New Class of Star‐Shaped Columnar Mesogens Exhibiting π–π Driven Supergelation,” The Journal of Physical Chemistry C 122 (2018): 23659–23674, 10.1021/acs.jpcc.8b05811.

[3] C. Wang , H. Dong , W. Hu , Y. Liu , and D. Zhu , “Semiconducting π‐Conjugated Systems in Field‐Effect Transistors: A Material Odyssey of Organic Electronics,” Chemical Reviews 112 (2012): 2208–2267, 10.1021/cr100380z. 22111507

[4] M. Antonijevic , C. Rochais , and P. Dallemagne , “C3‐Symmetric Ligands in Drug Design: When the Target Controls the Aesthetics of the Drug,” Molecules 28 (2023): 679, 10.3390/molecules28020679. 36677739 PMC9862528

[5] M. Bashiri , A. Jarrahpour , B. Rastegari , et al., “Synthesis and Evaluation of Biological Activities of Tripodal Imines and β‐lactams Attached to the 1,3,5‐Triazine Nucleus,” Monatshefte für Chemie—Chemical Monthly 151 (2020): 821–835, 10.1007/s00706-020-02592-8.

[6] J. L. Segura , R. Juárez , M. Ramos , and C. Seoane , “Hexaazatriphenylene (HAT) Derivatives: From Synthesis to Molecular Design, Self‐organization and Device Applications,” Chemical Society Reviews 44 (2015): 6850–6885, 10.1039/C5CS00181A. 26168289

[7] X. Wang , Z. Zhou , X. Lin , Z. Pei , D. Liu , and S. Zhao , Chemical Engineering Journal 427 (2022): 130995.

[8] S.‐W. Kim , H. Jung , M. S. Okyay , et al., Angewandte Chemie International Edition 62 (2023): e202310560. 37654107 10.1002/anie.202310560

[9] Z. Li , W. He , P. Zhang , J. Lei , and Q.‐G. Zhai , “Hexaazatrinaphthylene‐Based Ultrastable Metal–Organic Frameworks Modulated by the Chelating Coordination Configuration for CO_2_ Capture,” Inorganic Chemistry 64 (2025): 3057–3065, 10.1021/acs.inorgchem.4c05364. 39908016

[10] T. Ishi‐i , K. Yaguma , R. Kuwahara , Y. Taguri , and S. Mataka , “Self‐Assembling of n‐Type Semiconductor Tri(phenanthrolino)Hexaazatriphenylenes With a Large Aromatic Core,” Organic Letters 8 (2006): 585–588, 10.1021/ol052779t. 16468717

[11] B. Gao , J. Li , Y. Cheng , and L. Wang , “Hierarchical Self‐Assembled Nanostructures From the Organic n‐Type Semiconductor Hexaazatrinaphthylene,” Chemphyschem 10 (2009): 3197–3200, 10.1002/cphc.200900562. 19904798

[12] S. Sergeyev , W. Pisula , and Y. H. Geerts , “Discotic Liquid Crystals: A New Generation of Organic Semiconductors,” Chemical Society Reviews 36 (2007): 1902–1929, 10.1039/b417320c. 17982517

[13] B. R. Kaafarani , “Discotic Liquid Crystals for Opto‐Electronic Applications,” Chemistry of Materials 23 (2011): 378–396, 10.1021/cm102117c.

[14] S. M. Said , M. S. Mahmood , M. N. Daud , M. F. M. Sabri , and N. A. Sairi , “Structure‐Electronics Relations of Discotic Liquid Crystals From a Molecular Modelling Perspective,” Liquid Crystals 43 (2016): 2092–2113, 10.1080/02678292.2016.1209792.

[15] Z. Liu , Z. Wang , Y. Shi , et al., “A High‐capacity Hexaazatrinaphthylene Anode for Aqueous Organic Hybrid Flow Batteries,” Journal of Materials Chemistry A 9 (2021): 27028–27033, 10.1039/D1TA06138K.

[16] S. Li , Y. Liu , L. Dai , et al., “A Stable Covalent Organic Framework Cathode Enables Ultra‐Long Cycle Life for Alkali and Multivalent Metal Rechargeable Batteries,” Energy Storage Materials 48 (2022): 439–446, 10.1016/j.ensm.2022.03.033.

[17] J. Weng , Q. Xi , X. Zeng , et al., “Recent Progress of Hexaazatriphenylene‐Based Electrode Materials for Rechargeable Batteries,” Catalysis Today 400‐401 (2022): 102–114, 10.1016/j.cattod.2021.09.040.

[18] D. G. Velázquez , A. G. Orive , A. H. Creus , R. Luque , and Á. G. Ravelo , “Novel Organogelators Based on Amine‐derived Hexaazatrinaphthylene,” Organic & Biomolecular Chemistry 9 (2011): 6524, 10.1039/c1ob05811h. 21853165

[19] O. Morawski , P. Gawryś , J. Sadło , and A. L. Sobolewski , “Photochemical Hydrogen Storage With Hexaazatrinaphthylene,” Chemphyschem 23 (2022): e202200077, 10.1002/cphc.202200077. 35377513

[20] S. Marder , B. Kaafarani , S. Barlow , et al., U.S. Patent No. US 7994423 B2 (U.S. Patent and Trademark Office, 2011).

[21] D. Zhao , Z. Zhu , M. Y. Kuo , C. C. Chueh , and A. K.‐Y. Jen , “Hexaazatrinaphthylene Derivatives: Efficient Electron‐Transporting Materials With Tunable Energy Levels for Inverted Perovskite Solar Cells,” Angewandte Chemie International Edition 55 (2016): 8999–9003, 10.1002/anie.201604399. 27273656

[22] K. Isoda and K. Shimooka , “Synthesis and Characterization of Crown‐Ether Appended Hexaazatrinaphthylene‐Based Liquid‐Crystalline Derivative,” Crystals 10 (2020): 377, 10.3390/cryst10050377.

[23] A. Bedi and O. Gidron , “The Consequences of Twisting Nanocarbons: Lessons From Tethered Twisted Acenes,” Accounts of Chemical Research 52 (2019): 2482–2490, 10.1021/acs.accounts.9b00271. 31453688

[24] R. Mokrai , R. Szűcs , M. P. Duffy , et al., “Topologically Diverse Polycyclic Aromatic Hydrocarbons From Pericyclic Reactions With Polyaromatic Phospholes,” New Journal of Chemistry 45 (2021): 8118–8124, 10.1039/D1NJ01194D.

[25] H. V. Anderson , N. D. Gois , and W. A. Chalifoux , “New Advances in Chiral Nanographene Chemistry,” Organic Chemistry Frontiers 10 (2023): 4167–4197, 10.1039/D3QO00517H.

[26] Z. Zhou and M. A. Petrukhina , “Planar, Curved and Twisted Molecular Nanographenes: Reduction‐Induced Alkali Metal Coordination,” Coordination Chemistry Reviews 486 (2023): 215144, 10.1016/j.ccr.2023.215144.

[27] S. Choudhary , C. Gozalves , A. Higelin , I. Krossing , M. Melle‐Franco , and A. Mateo‐Alonso , “Hexaazatrinaphthylenes With Different Twists,” Chemistry—A European Journal 20 (2014): 1525–1528, 10.1002/chem.201304071. 24323954

[28] X.‐Y. Liu , T. Usui , and J. Hanna , “Self‐Directed Orientation of Molecular Columns Based on n‐Type Hexaazatrinaphthylenes (HATNAs) for Electron Transport,” Chemistry—A European Journal 20 (2014): 14207–14212, 10.1002/chem.201403472. 25223273

[29] CCDC, 2488187 for 3c and CCDC, 2487906 for 3m contain the supplementary crystallographic data. These data are provided free of charge by the joint Cambridge Crystallographic Data Centre and Fachinformationszentrum Karlsruhe, (http://www.ccdc.cam.ac.uk/structures).

[30] Using the nudged elastic band (NEB) method, we roughly estimated the flipping energies for two of the six arylethynyl moieties of 3g and included the results in the Supplementary Information.

[31] We performed DFT and TD‐DFT calculations for 3c, 3e, and 3h, which reasonably explain the differences of ε: see the Supporting Information in detail.

[32] O. Morawski , J. Kapiuk , P. Gawryś , and A. L. Sobolewski , “Aggregation Controlled Photoluminescence of Hexaazatri‐naphthylene (HATN)—An Experimental and Theoretical Study,” Physical Chemistry Chemical Physics 22 (2020): 15437–15447, 10.1039/D0CP01289K. 32602508

