# Supplementary material for: Synthesis of Aryl‐Substituted Hexa‐Alkynyl Hexaazatrinaphthylenes via Sonogashira Coupling and Evaluation of Their Photophysical Properties
Source: Chem Asian J. 2026 Feb 16;21(4):e01003. doi: 10.1002/asia.202501003 (PMC12909292; doi:10.1002/asia.202501003)
Supplement: Supplementary file 1 — Experimental details, analytical data, and NMR spectral copies of substrates and products (PDF).Supporting File 1: asia70632‐sup‐0001‐SuppMat.pdf. [file ASIA-21-e01003-s001.pdf]

Supporting Information

Synthesis of Aryl-Substituted Hexa-alkynyl Hexaazatrinaphthylenes via Sonogashira Coupling and Evaluation of their Photophysical Properties

Yuchen Wu,[a] Chisae Kumagai,[a] Natsuhiko Sugimura,[b] and Takanori Shibata*[a]

[a] Department of Chemistry and Biochemistry, School of Advanced Science and Engineering,
Waseda University, 3-4-1 Okubo, Shinjuku, Tokyo 169-8555, Japan
E-mail: tshibata@waseda.jp
[b] Materials Characterization Central Laboratory
Waseda University, 3-4-1 Okubo, Shinjuku, Tokyo 169-8555, Japan

Table of Content

1. General information **S2**
2. Procedure for Aryl alkynes synthesis **S2**
3. General procedure of hexaazatrinaphthylenes 3 **S4**
4. Physical properties of new compounds **S4**
5. Solvent effect in photophysical properties **S12**
6. DFT and TD-DFT calculations for 3c, 3e, and 3h **S13**
7. Flipping energy of 3g calculated by NEB method **S25**
8. Crystal data **S39**
9. References **S41**
10. ^{1}H- and ^{13}C-NMR spectra **S42**

1. General information

^{1}H NMR spectra were recorded with JEOL ECS400 (400 MHz). The chemical shifts were reported in parts per million (δ) relative to internal standard TMS (0.0 ppm). ^{13}C NMR spectra were recorded with BRUKER AVANCE NEO (150 MHz) and JEOL ECS (101 MHz). The chemical shifts were reported in parts per million (δ) relative to internal solvent signals (77.16 ppm in $CDCl_3$). Data are presented as follows: chemical shift, multiplicity (s = singlet, d = doublet, dd = double doublet, m = multiplet), coupling constants, *J*, are reported in Hertz (Hz), and integration. High-resolution mass spectra (HRMS) were measured on a Bruker QTOF compact (positive mode) source with APCI II. Ultraviolet spectrum was measured on a JASCO V-630 spectrometer. Fluorescence spectra were measured on a JASCO FP-8200 spectrofluorometer. X-ray structures were obtained by a Rigaku XtaLAB Synergy-S diffractometer. Preparative thin-layer chromatography (PTLC) was performed with silica gel-precoated glass plates (Merck 60 GF254) prepared in our laboratory; flash column chromatography was performed over silica gel 40-50 μm purchased from Kanto Chemical. Unless otherwise stated, all reagents were weighed and handled in air, and all reactions were performed under an argon atmosphere and heated with an oil bath. Solvents were degassed by bubbling argon gas through the liquid before injecting them into the reaction vessel if necessary. All reagents were purchased from BLDpharm, Fujifilm Wako Pure Chemical Co., Kanto Chemical Co., Inc., Sigma-Aldrich Co., and Tokyo Chemical Industry Co., Ltd.

2. Procedure for Aryl alkynes synthesis

Alkynes **2a**, **2b**, **2c**, **2d**, **2e, 2f**, **2g**, **2k**, and **2i** were obtained from commercial sources. Alkyne **2h** was synthesized according to the reported procedure.[1]

1-Ethynyl-4-methylnaphthalene (2j)

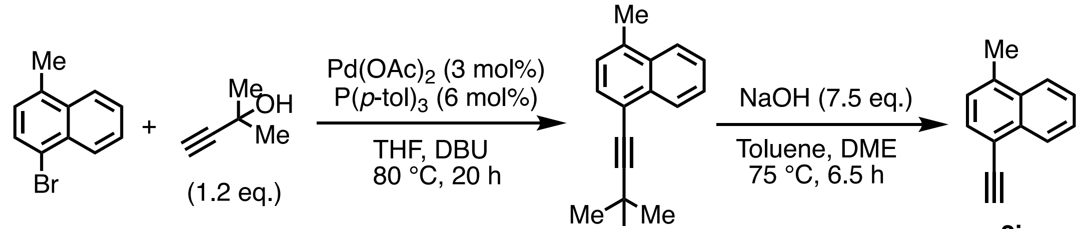

2j

A flame-dried 50 mL two-necked round-bottom flask equipped with a magnetic stir bar was charged with palladium(II) acetate (0.15 mmol, 33.7 mg, 3 mol%) and tri(*p*-tolyl)phosphine (0.30 mmol, 91.3 mg, 6 mol%) and sealed with a rubber septum. The vessel was evacuated and backfilled with argon three times. Dehydrated THF (10 mL), 1-bromo-4-methylnaphthalene (5.0 mmol, 0.76 mL) in dehydrated THF (5 mL), 1,8-diazabicyclo[5.4.0]undec-7-ene (15 mmol, 2.3 mL, 3.0 eq.), and 2-methyl-3-butyn-2-ol (6.0 mmol, 0.6 mL, 1.2 eq.) were added. The reaction mixture was heated at 80 °C in a preheated oil bath for 20 h, then cooled to room temperature and extracted with ethyl acetate. The solvent was removed under reduced pressure, and the residue was purified by flash column

chromatography on silica gel (hexane/ethyl acetate = 1:1) to afford propargyl alcohol (1.14 g, >99% yield).

The obtained product (5 mmol, 1.12 g) and NaOH (37.5 mmol, 1.50 g) were placed in a flame-dried 200 mL two-necked round-bottom flask equipped with a magnetic stir bar and sealed with a rubber septum. The flask was evacuated and backfilled with argon three times. Toluene (50 mL) and 1,2-dimethoxyethane (6.0 mL) were added, and the mixture was heated at 75 °C in a preheated oil bath for 6.5 h. After cooling to room temperature, the mixture was washed with 1 N HCl and brine. The solvent was removed under reduced pressure, and the residue was purified by flash column chromatography on silica gel (hexane) to give alkyne **2j** (634.4 mg, 76% yield).

2-Ethynyl-8-methylquinoline (2m)

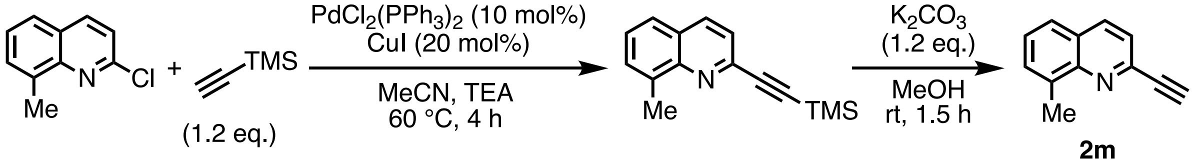

2m

A flame-dried 30 mL two-necked round-bottom flask equipped with a magnetic stir bar was charged with 2-chloro-8-methylquinoline (0.77 mmol, 165.2 mg), bis(triphenylphosphine)-palladium(II) dichloride (0.077 mmol, 54.1 mg, 10 mol%), and copper(I) iodide (0.154 mmol, 29.3 mg, 20 mol%) and sealed with a rubber septum. The vessel was evacuated and backfilled with argon three times. Dehydrated MeCN (1.9 mL), triethylamine (0.5 mL), and trimethylsilylacetylene (1.46 mmol, 0.20 mL, 2.0 eq.) were added. The mixture was heated at 60 °C in a preheated oil bath for 4 h, then cooled to room temperature. The solvent was removed under reduced pressure, and the residue was extracted with ethyl acetate and purified by preparative TLC to afford the product in TMS-alkyne (181.2 mg, 97% yield).

The obtained product (0.75 mmol, 181.2 mg) and K_2CO_3 (0.90 mmol, 124.4 mg) were placed in a flame-dried 30 mL two-necked round-bottom flask equipped with a magnetic stir bar and sealed with a rubber septum. The vessel was evacuated and backfilled with argon three times. MeOH (10 mL) was added, and the mixture was stirred for 1.5 h at room temperature. The solvent was removed under reduced pressure, and the residue was extracted with ethyl acetate to afford alkyne **2m** (119.5 mg, 95% yield).

2,7-Di-tert-butyl-4-ethynylpyrene (2l)

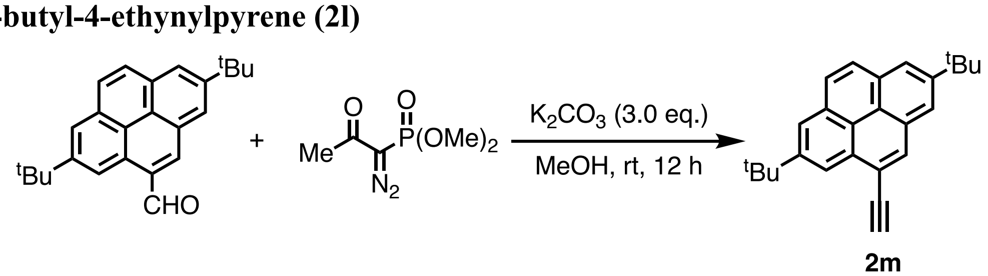

2m

2,7-Di-tert-butylpyrene-4-carbaldehyde (1.0 mmol, 342.5 mg, synthesized according to the

reported procedure [2]) and K_2CO_3 (3.0 mmol, 414.6 mg, 3.0 eq.) were placed in a flame-dried 30 mL two-necked round-bottom flask equipped with a magnetic stir bar and sealed with a rubber septum. The flask was evacuated and backfilled with argon three times. Dimethyl (1-diazo-2-oxopropyl)phosphonate (1.5 mmol, 288.1 mg, 1.5 eq.) and MeOH (4.0 mL) were then added, and the mixture was stirred for 12 h at room temperature. The solvent was removed under reduced pressure, and the residue was quenched with aqueous NH_4Cl solution. The crude mixture was extracted with ethyl acetate and purified by flash column chromatography on silica gel (hexane) to afford alkyne **2m** (144.3 mg, 43% yield).

3. General procedure of hexaazatrinaphthylenes 3

A flame-dried sealed tube equipped with a magnetic stir bar was charged with 1,4,7,10,13,16-hexabromodiquinoxaline **1** (0.025 mmol, synthesized according to the reported procedure[3]) , aryl alkyne **2** (0.30 mmol, 12 eq.), bis(triphenylphosphine)palladium(II) dichloride (0.0075 mmol, 30 mol%), triphenylphosphine (0.015 mmol, 60 mol%), and copper(I) iodide (0.0075 mmol, 30 mol%), and then sealed with a rubber septum. The vessel was evacuated and backfilled with argon three times. DMSO (1.5 mL) and diisopropylamine (1.5 mL) were added. The rubber septum was quickly replaced with a screw cap under argon flow, and the reaction mixture was heated at 80 °C in a preheated oil bath for 23 h. After cooling to room temperature, the mixture was filtered to remove precipitates and extracted with dichloromethane. The crude product was passed through a pad of silica gel, and the red fraction was collected. The solvent was removed under reduced pressure, and the residue was purified by preparative TLC to afford the pure product.

4. Physical properties of new compounds

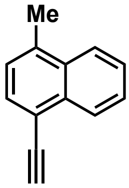

1-Ethynyl-4-methylnaphthalene (2j).

Isolated by preparative TLC (hexane, Rf = 0.5). The title compound was obtained as colorless oil. ^{1}H NMR (400 MHz, $CDCl_3$) δ 8.40-8.37 (m, 1H), 7.98-7.96 (m, 1H), 7.62 (d, J = 7.3 Hz, 1H), 7.58-7.50 (m, 2H), 7.23 (d, J = 7.3 Hz, 1H), 3.42 (s, 1H), 2.66 (s, 3H); ^{13}C NMR (101 MHz, $CDCl_3$) δ 136.3, 133.6, 132.4, 131.1, 126.8, 126.7, 126.4, 126.1, 124.6, 118.1, 82.2, 81.5, 19.8; HRMS (APCI II positive) m/z: $[M+H]^+$ Calcd. for $C_{13}H_{11}$, 167.0855; Found, 167.0861.

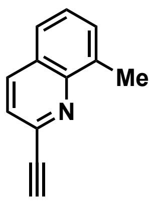

2-Ethynyl-8-methylquinoline (2m).
Isolated by preparative TLC (hexane/ethyl acetate = 10/1, Rf = 0.5). The title compound was obtained as a yellow solid. Mp = 77-78 °C; ^{1}H NMR (400 MHz, $CDCl_3$) δ 8.01 (d, *J* = 8.2 Hz, 1H), 7.57-7.47 (m, 3H), 7.40-7.36 (m, 1H), 3.24 (s, 1H), 2.80 (s, 3H); ^{13}C NMR (101 MHz, $CDCl_3$) δ 147.2, 141.3, 137.3, 136.4, 130.1, 127.4, 127.1, 125.5, 124.0, 83.9, 77.2, 18.1; HRMS (APCI II positive) m/z: $[M+H]^+$ Calcd. for $C_{12}H_{10}N$, 168.0808; Found, 168.0813.

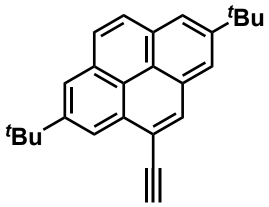

2,7-Di-*tert*-butyl-4-ethynylpyrene (2l).
Isolated by preparative TLC (hexane, Rf = 0.8). The title compound was obtained as a white solid. Mp = 127-128 °C; ^{1}H NMR (400 MHz, $CDCl_3$) δ 8.70 (d, *J* = 1.8 Hz, 1H), 8.35 (s, 1H), 8.22-8.20 (m, *J* = 6.9 Hz, 2H), 8.15 (d, *J* = 1.8 Hz, 1H), 8.05-7.99 (m, 2H), 3.55 (s, 1H), 1.60 (s, 9H), 1.57 (s, 9H); ^{13}C NMR (101 MHz, $CDCl_3$) δ 149.2, 149.1, 133.6, 131.0, 130.8, 130.0, 129.8, 127.8, 127.4, 123.4, 122.9, 122.7, 122.7, 122.4, 121.0, 119.2, 82.4, 81.6, 35.6, 35.4, 32.1, 32.0; HRMS (APCI II positive) m/z: $[M+H]^+$ Calcd. for $C_{26}H_{26}$, 339.2107; Found, 339.2112.

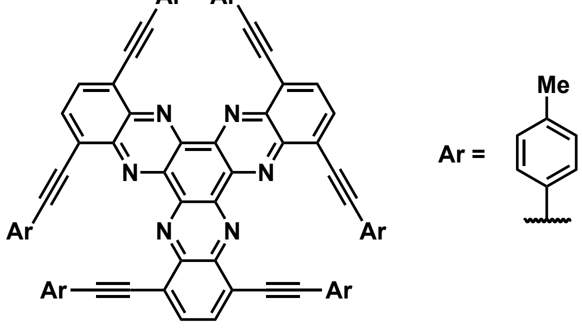

1,4,7,10,13,16-Hexakis(*p*-tolylethynyl)diquinoxalino[2,3-*a*:2',3'-*c*]phenazine (3a).
Isolated by preparative TLC (dichloromethane, Rf = 0.3). The title compound was obtained as a red solid (18.7 mg, 70%). Mp = >300 °C; ^{1}H NMR (400 MHz, $CDCl_3$) δ 8.19 (s, 6H), 7.49 (d, *J* = 7.8 Hz, 12H), 6.82 (d, *J* = 7.8 Hz, 12H), 2.19 (s, 18H); ^{13}C NMR (101 MHz, $CDCl_3$) δ 143.9, 143.4, 138.6, 135.6, 132.2, 128.7, 125.1, 119.8, 100.0, 86.6, 21.6; HRMS (APCI II positive) m/z: $[M+H]^+$ Calcd. for

$C_{78}H_{49}N_6$, 1069.4013; Found,1069.3971.

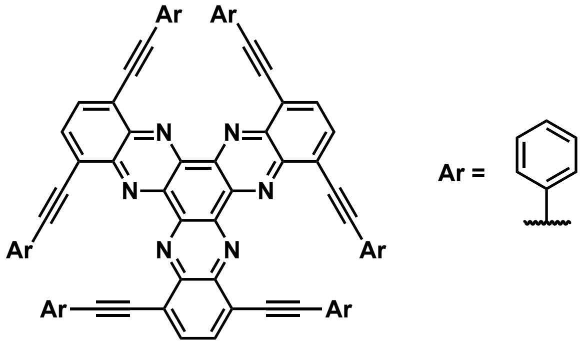

1,4,7,10,13,16-Hexakis(phenylethynyl)diquinoxalino[2,3-*a*:2',3'-*c*]phenazine (3b).
Isolated by preparative TLC (hexane/dichloromethane = 10/1, Rf = 0.4). The title compound was obtained as an orange solid (10.5 mg, 43%). Mp = >300 °C; ^{1}H NMR (400 MHz, $CDCl_3$) δ 8.27 (s, 6H), 7.66-7.64 (m, 12H), 7.10-7.06 (m, 18H); ^{13}C NMR (101 MHz, $CDCl_3$) δ 143.9, 143.5, 135.8, 132.4, 128.7, 128.1, 125.2, 122.8, 99.9, 87.0; HRMS (APCI II positive) m/z: $[M+H]^+$ Calcd. for $C_{72}H_{37}N_6$, 984.3074; Found, 985.3087.

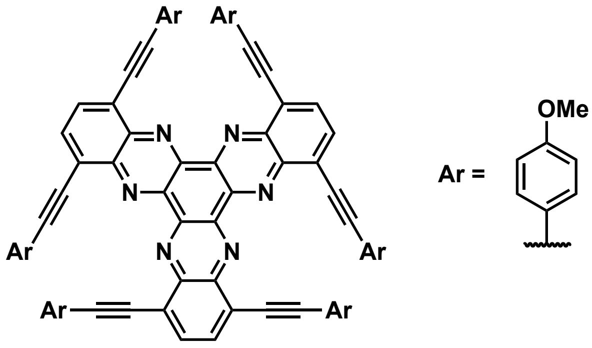

1,4,7,10,13,16-Hexakis((4-methoxyphenyl)ethynyl)diquinoxalino[2,3-*a*:2',3'-*c*]phenazine (3c).
Isolated by preparative TLC (dichloromethane/MeOH = 100/1, Rf = 0.5). The title compound was obtained as a deep red solid (20.1 mg, 69%). Mp = >300 °C; ^{1}H NMR (400 MHz, $CDCl_3$) δ 8.22 (s, 6H), 7.58 (d, J = 8.7 Hz, 12H), 6.57 (d, J = 8.7 Hz, 12H), 3.73 (s, 18H); ^{13}C NMR (101 MHz, $CDCl_3$) δ 159.9, 143.9, 143.4, 135.4, 133.9, 125.1, 115.1, 113.7, 100.0, 86.3, 55.2; HRMS (APCI II positive) m/z: $[M+H]^+$ Calcd. for $C_{78}H_{49}N_6O_6$, 1165.3708; Found, 1165.3683.

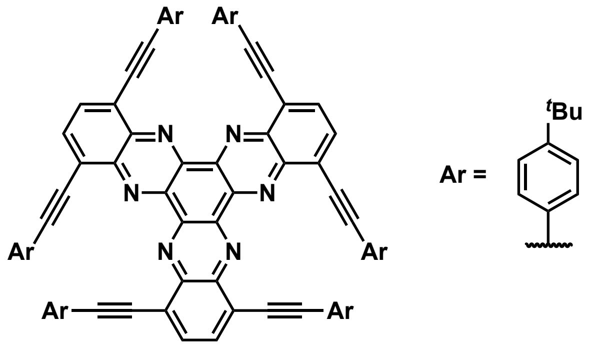

1,4,7,10,13,16-Hexakis((4-(*tert*-butyl)phenyl)ethynyl)diquinoxalino[2,3-*a*:2',3'-*c*]phenazine (3d). Isolated by preparative TLC (hexane/dichloromethane = 1/1, Rf = 0.3). The title compound was obtained as a red solid (21.1 mg, 64%). Mp = >300 °C; ^{1}H NMR (400 MHz, $CDCl_3$) δ 8.23 (s, 6H), 7.62 (d, J = 8.7 Hz, 12H), 7.12 (d, J = 8.7 Hz, 12H), 1.20 (s, 54H); ^{13}C NMR (101 MHz, $CDCl_3$) δ 151.7, 143.8, 143.6, 135.2, 132.3, 125.2, 125.1, 120.0, 100.4, 86.7, 34.8, 31.3; HRMS (APCI II positive) m/z: $[M+H]^+$ Calcd. for $C_{96}H_{85}N_6$, 1321.6830; Found, 1321.6837.

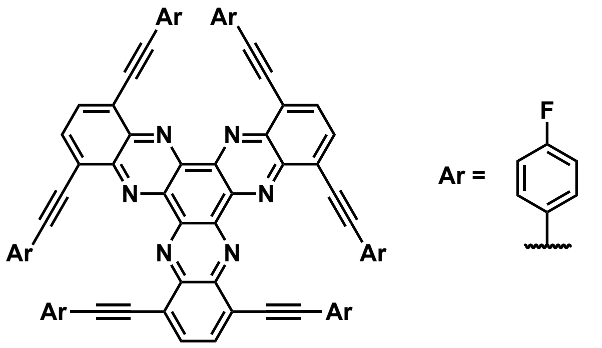

1,4,7,10,13,16-Hexakis((4-fluorophenyl)ethynyl)diquinoxalino[2,3-*a*:2',3'-*c*]phenazine (3e). Isolated by preparative TLC (dichloromethane, Rf = 0.6). The title compound was obtained as an orange solid (17.9 mg, 66%). Mp = >300 °C; ^{1}H NMR (400 MHz, $CDCl_3$) δ 8.23 (s, 6H), 7.64-7.60 (m, 12H), 6.82-6.78 (m, 12H);^{13}C NMR (101 MHz, $CDCl_3$) δ 162.8 (d, J_{C-F} = 251.1 Hz), 143.9, 143.4, 135.5, 134.2 (d, J_{C-F} = 8.6 Hz), 125.0, 118.9, 115.5 (d, J_{C-F} = 22.0 Hz), 98.7, 86.7; HRMS (APCI II positive) m/z: $[M+H]^+$ Calcd. for $C_{72}H_{31}F_6N_6$, 1093.2509; Found, 1093.2529.

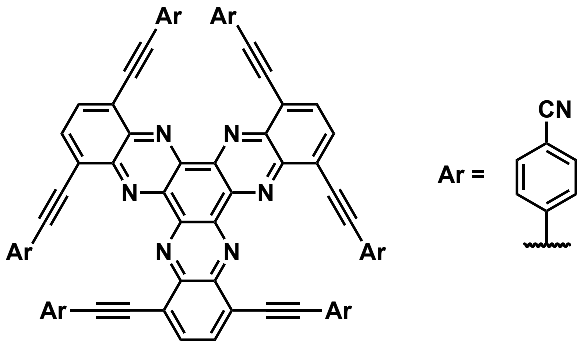

4,4',4'',4''',4'''',4'''''-(Diquinoxalino[2,3-*a*:2',3'-*c*]phenazine-1,4,7,10,13,16-hexaylhexakis(ethyne-2,1-diyl))hexabenzonitrile (3f).

Isolated by preparative TLC (dichloromethane/MeOH = 100/1, Rf = 0.2). The title compound was obtained as a yellow solid (9.3 mg, 35%). Mp = >300 °C; ^{1}H NMR (400 MHz, $CDCl_3$) δ 8.34 (s, 6H), 7.72 (d, J = 8.2 Hz, 12H), 7.43 (d, J = 8.2 Hz, 12H); HRMS (APCI II positive) m/z: $[M+H]^+$ Calcd. for $C_{78}H_{31}N_{12}$, 1135.2789; Found, 1135.2826. We could not measure the C NMR of this compound due to its low solubility for any deuterated solvents.

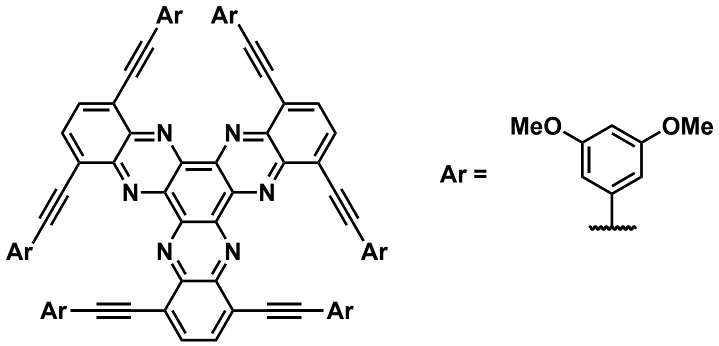

1,4,7,10,13,16-Hexakis((3,5-dimethoxyphenyl)ethynyl)diquinoxalino[2,3-*a*:2',3'-*c*]phenazine (3g).

Isolated by preparative TLC (dichloromethane/MeOH = 50/1, Rf = 0.3). The title compound was obtained as an orange solid (25.3 mg, 75%). Mp = >300 °C; ^{1}H NMR (400 MHz, $CDCl_3$) δ 8.22 (s, 6H), 6.72 (d, J = 2.3 Hz, 12H), 6.19 (dd, J = 2.3, 2.3 Hz, 6H), 3.75 (s, 36H); ^{13}C NMR (150 MHz, $CDCl_3$): δ 159.9, 143.9, 143.5, 135.6, 125.1, 123.7, 109.6, 102.7, 99.9, 86.0, 55.28; HRMS (APCI II positive) m/z: $[M+H]^+$ Calcd. for $C_{84}H_{61}N_6O_{12}$, 1345.4342; Found, 1345.4368.

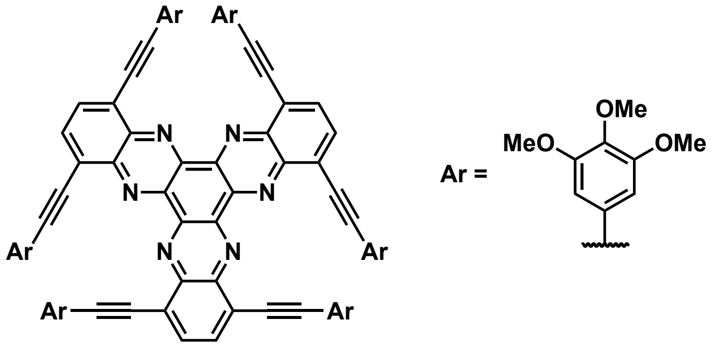

1,4,7,10,13,16-Hexakis((3,4,5-trimethoxyphenyl)ethynyl)diquinoxalino[2,3-*a*:2',3'-*c*]phenazine (3h).

Isolated by preparative TLC (dichloromethane/MeOH =20/1, Rf = 0.4). The title compound was obtained as a deep red solid (24.4 mg, 64%). Mp = >300 °C; ^{1}H NMR (400 MHz, $CDCl_3$) δ 8.21 (s, 6H), 6.87 (s, 12H), 3.86 (s, 36H), 3.78 (s, 18H); ^{13}C NMR (101 MHz, $CDCl_3$) δ 152.7, 143.9, 143.7, 139.3, 134.9, 125.0, 117.5, 109.5, 100.7, 86.1, 60.9, 56.2; HRMS (APCI II positive) m/z: $[M+H]^+$ Calcd. for $C_{90}H_{73}N_6O_{18}$, 1525.4976; Found,1525.4934.

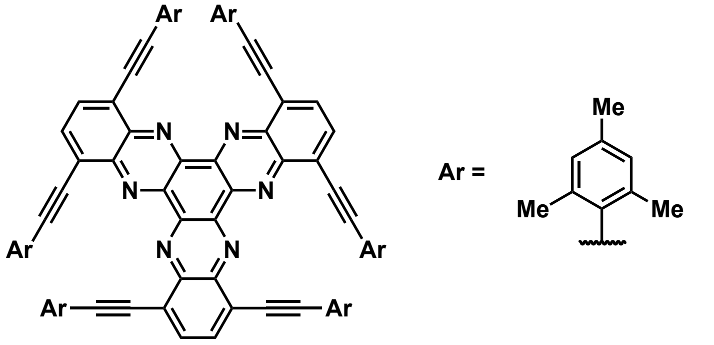

1,4,7,10,13,16-Hexakis(mesitylethynyl)diquinoxalino[2,3-*a*:2',3'-*c*]phenazine (3i).

Isolated by preparative TLC (hexane/dichloromethane =2/1, Rf = 0.4). The title compound was obtained as a red solid (11.8 mg, 38%). Mp = >300 °C; ^{1}H NMR (400 MHz, $CDCl_3$) δ 8.07 (s, 6H), 6.48 (s, 12H), 2.51 (s, 36H), 2.14 (s, 18H); ^{13}C NMR (101 MHz, $CDCl_3$) δ 144.1, 143.6, 141.0, 137.6, 133.7, 126.9, 125.1, 119.7, 98.0, 94.8, 21.6, 21.5; HRMS (APCI II positive) m/z: $[M+H]^+$ Calcd. for $C_{90}H_{73}N_6$, 1237.5891; Found, 1237.5873.

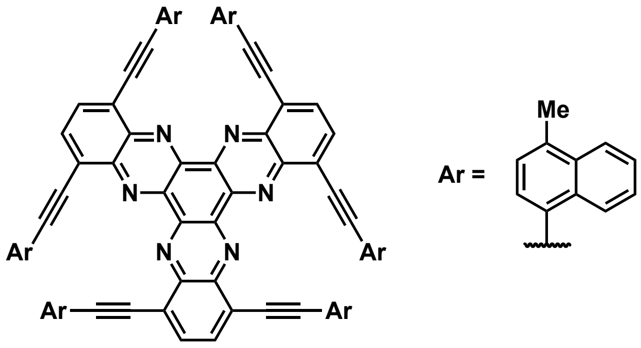

1,4,7,10,13,16-Hexakis((4-methylnaphthalen-1-yl)ethynyl)diquinoxalino[2,3-*a*:2',3'-*c*]phenazine (3j).

Isolated by preparative TLC (hexane/dichloromethane =1/5, Rf = 0.1). The title compound was obtained as a red solid (19.7 mg, 58%). Mp = >300 °C; ^{1}H NMR (400 MHz, $CDCl_3$) δ 8.63 (d, *J* = 7.8 Hz, 6H), 8.25 (s, 6H), 7.57 (d, *J* = 8.2 Hz, 6H), 7.44-7.32 (m, 18H), 6.60 (d, *J* = 7.3 Hz, 6H), 2.25 (s, 18H); ^{13}C NMR (150 MHz, $CDCl_3$) δ 144.0, 143.7, 135.4, 134.4, 133.0, 131.6, 130.9, 127.8, 126.7, 125.8, 125.6, 125.2, 123.7, 118.7, 98.6, 91.9, 19.5; HRMS (APCI II positive) m/z: $[M+H]^+$ Calcd. for $C_{102}H_{61}N_6$, 1369.4925; Found, 1369.4952.

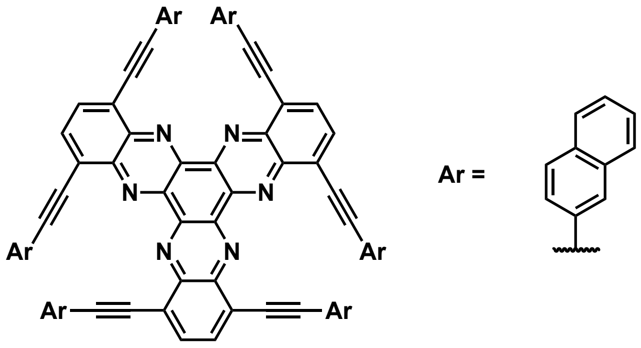

1,4,7,10,13,16-Hexakis(naphthalen-2-ylethynyl)diquinoxalino[2,3-*a*:2',3'-*c*]phenazine (3k).

Isolated by preparative TLC (hexane/dichloromethane =1/5, Rf = 0.1). The title compound was obtained as an orange solid (14.2 mg, 41%). Mp = >300 °C; ^{1}H NMR (400 MHz, $CDCl_3$) δ 8.37 (s, 6H), 8.05 (s, 6H), 7.54 (d, *J* = 7.8 Hz, 6H), 7.46 (d, *J* = 8.2 Hz, 6H), 7.36-7.29 (m, 12H), 7.17 (d, *J* = 8.2 Hz, 6H); ^{13}C NMR (150 MHz, $CDCl_3$) δ 144.2, 143.8, 135.8, 132.8, 132.7, 132.4, 128.3, 128.0, 127.4, 127.3, 126.5, 125.9, 125.4, 119.9, 100.6, 87.3; HRMS (APCI II positive) m/z: $[M+H]^+$ Calcd. for $C_{96}H_{49}N_6$, 1285.4013; Found, 1285.3994.

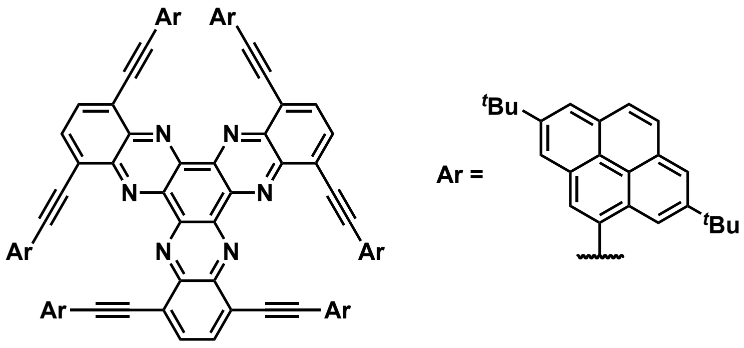

1,4,7,10,13,16-Hexakis((2,7-di-*tert*-butylpyren-4-yl)ethynyl)diquinoxalino[2,3-*a*:2',3'-*c*]phenazine (3l).

Isolated by preparative TLC (hexane/dichloromethane =1/1, Rf = 0.5). The title compound was obtained as a deep red solid (75%). Mp = >300 °C; ^{1}H NMR (400 MHz, $CDCl_3$) δ 8.68 (d, *J* = 1.8 Hz, 6H), 8.47 (s, 6H), 8.10 (s, 6H), 7.84 (d, *J* = 1.4 Hz, 6H), 7.79-7.76 (m, 12H), 7.45 (q, *J* = 8.7 Hz, 12H), 1.62 (s, 54H), 1.55 (s, 54H); ^{13}C NMR (101 MHz, $CDCl_3$) δ 148.0, 147.6, 144.6, 144.2, 135.3, 133.6, 130.1, 129.8, 129.3, 128.8, 126.9, 126.3, 125.9, 122.8, 122.7, 122.2, 121.8, 121.5, 120.5, 119.4, 99.5, 91.4, 35.3, 35.2, 32.2, 32.0; HRMS (APCI II positive) m/z: $[M+H]^{+}$ Calcd. for $C_{180}H_{157}N_6$, 2402.2464; Found, 2402.2473.

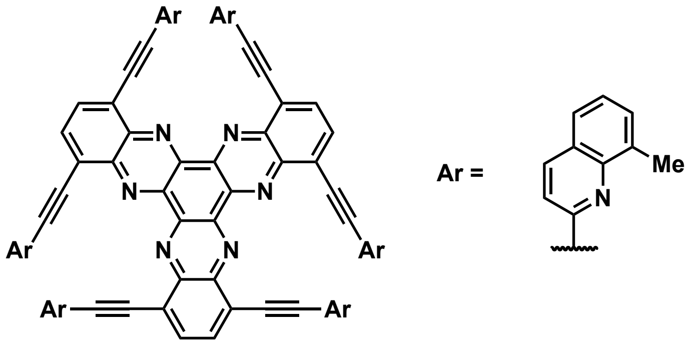

1,4,7,10,13,16-Hexakis((8-methylquinolin-2-yl)ethynyl)diquinoxalino[2,3-*a*:2',3'-*c*]phenazine (3m).

Isolated by preparative TLC (dichloromethane/MeOH =20/1, Rf = 0.4). The title compound was obtained as an orange solid (13.1 mg, 38%). Mp = >300 °C; ^{1}H NMR (400 MHz, $CDCl_3$) δ 8.50 (s, 6H), 7.72 (d, *J* = 8.2 Hz, 6H), 7.55 (d, *J* = 8.2 Hz, 6H), 7.31 (m, 6H), 7.12 (m, 12H), 2.74 (s, 18H); NMR (150 MHz, $CDCl_3$) δ 146.8, 144.2, 144.1, 142.0, 137.0, 136.8, 135.8, 129.8, 126.8, 126.7, 125.3, 125.2, 125.0, 100.1, 86.4, 18.4; HRMS (APCI II positive) m/z: $[M+H]^{+}$ Calcd. for $C_{96}H_{55}N_{12}$, 1375.4667; Found, 1375.4644.

5. Solvent effect in photophysical properties

We examined the absorption and emission behaviors of **3a** in other solvents. However, except in THF, the spectra measurements were failed due to the extremely low solubility. The measurement of **3a** in THF solution was shown in Figure S1 and Table S1. A general decrease of the absorbance was observed when changing the solvent to THF. Also, the slight hypsochromic shift of both absorption maximum wavelength (2 nm) and emission maximum wavelength (17 nm) were also ascertained. These photophysical property changes were attributed to the difference of the solvent polarity.

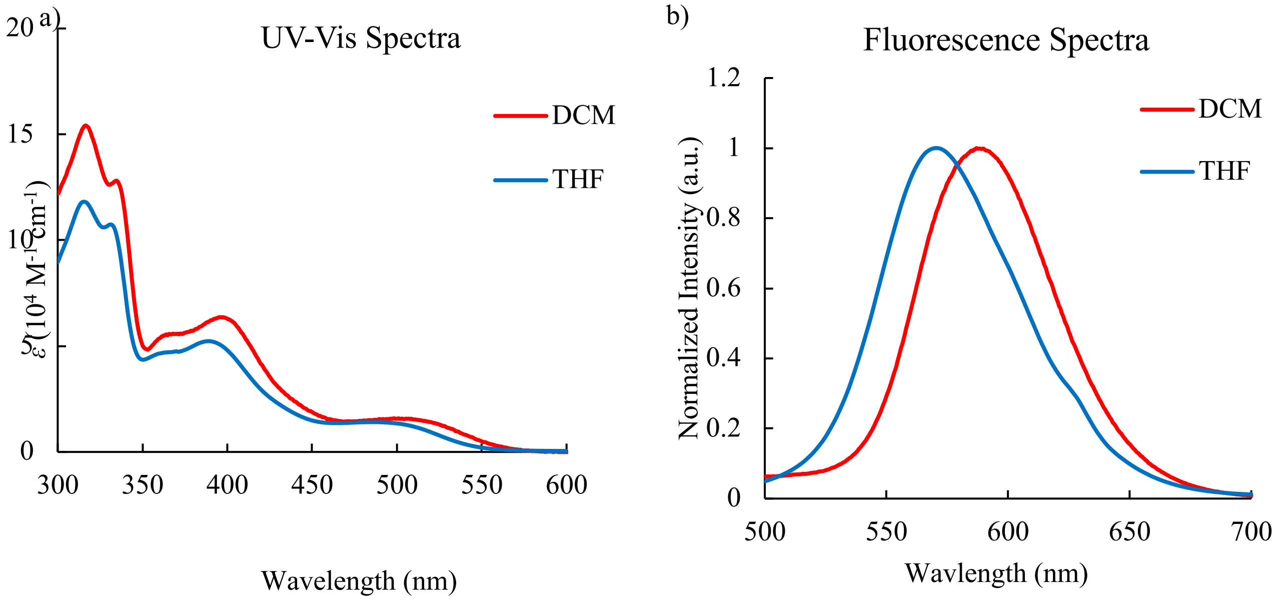

Figure S1. UV-vis and Fluorescence Spectra in DCM and THF

Table S1. Comparison of Photophysical Properties of **3a** in

Entry	$\lambda_{max\ (abs)}$ [nm] (ε [×10^4 M^{-1} cm^{-1}]) [a,b]	$\lambda_{max\ (em)}$ / nm [a,b]
**3a** in DCM	317(15.4), 335(12.8), 396(6.4), 500(1.6)	587
**3a** in THF	315(11.8), 331(10.7), 389(5.2), 485(1.4)	570

[a] **3a** in DCM: 5.6×10^{-5} M, **3a** in THF: 2.5×10^{-5} M [b] Excitation wavelengths: **3a** in DCM: 317 nm, **3a** in THF: 315 nm.

6. DFT and TD-DFT calculations for 3c, 3e, and 3h

Comp	ε_max	HOMO/ eV	LUMO / eV	ΔE/ eV	Oscillator Strength f	Dihedral angles /deg
**3c**	163000	-6.7928	-1.1478	5.6450	0.0859	31.4
**3e**	114000	-7.2864	-1.4520	5.8344	0.0858	33.4
**3h**	126000	-6.8959	-1.2504	5.6455	0.1081	38.8

SI Cartesian coordinates

The ground state of **3c**, **3e**, and **3h** are calculated at ωB97XD/6-31G(d,p) level using the Gaussian 16 program. The molecular structure information for each compound are listed below.

Cartesian coordinates

Atomic number	x	y	z
**3c** ground state			
7	0.64021	2.68972	−0.12943
8	7.48222	7.78595	−1.89574
6	0.31379	1.41959	−0.02615
6	−0.05203	4.98500	−0.27493
6	−0.35860	3.59442	−0.10532
6	1.26675	5.41320	−0.58712
6	−1.07797	5.90231	−0.14673
1	−0.85658	6.95670	−0.26861
6	2.35486	5.84465	−0.89802
6	3.65470	6.32633	−1.22992
6	6.25592	7.24521	−1.71297
6	4.68309	5.43056	−1.53811
1	4.47255	4.36623	−1.54777
6	3.94409	7.70070	−1.19857
1	3.15163	8.40622	−0.97145
6	5.97443	5.87960	−1.78091
1	6.75261	5.15516	−1.98413
6	5.22654	8.15530	−1.43934
1	5.46565	9.21292	−1.41366
6	8.56966	6.90649	−2.09089
1	8.46410	6.33667	−3.02234
1	8.67310	6.21184	−1.24922
1	9.45773	7.53503	−2.15448
7	−2.03160	1.87710	0.12987

8	−10.55281	2.30273	1.89474
6	−1.05321	1.00371	0.02764
6	−2.73460	4.16930	0.27345
6	−1.70568	3.18476	0.10475
6	−4.06854	3.79108	0.58581
6	−2.39309	5.50242	0.14447
1	−3.16408	6.25504	0.26572
6	−5.21263	3.54393	0.89683
6	−6.56049	3.22070	1.22930
6	−9.23303	2.53627	1.71239
6	−6.91618	1.90428	1.53831
1	−6.14879	1.13731	1.54864
6	−7.56602	4.20128	1.19702
1	−7.30046	5.22831	0.96894
6	−8.23893	1.55861	1.78129
1	−8.48207	0.52386	1.98577
6	−8.88455	3.86513	1.43772
1	−9.67197	4.61056	1.41103
6	−10.96643	0.96679	2.09031
1	−10.56224	0.55280	3.02234
1	−10.66465	0.33175	1.24929
1	−12.05413	0.99427	2.15292
7	2.63909	0.82082	0.13081
8	7.27476	7.98644	1.89313
6	1.39364	0.41006	0.02797
6	4.97565	0.28391	0.27492
6	3.60871	−0.11508	0.10561
6	5.31478	1.62816	0.58806
6	5.95966	−0.67807	0.14543
1	6.99686	−0.38656	0.26711
6	5.67330	2.74229	0.89940
6	6.06889	4.07085	1.23135
6	6.81555	6.72706	1.71223
6	5.10792	5.03828	1.54051
1	4.05974	4.75826	1.55185
6	7.42127	4.44984	1.19828
1	8.17699	3.70537	0.97031
6	5.47147	6.35643	1.78244

1	4.69778	7.08535	1.98668
6	7.79096	5.75955	1.43792
1	8.83053	6.06765	1.41050
6	6.32612	9.01396	2.08905
1	5.76623	8.87223	3.02172
1	5.62451	9.07068	1.24871
1	6.89506	9.94147	2.15056
7	−2.65072	−0.78923	−0.12871
8	−10.48871	2.58151	−1.89519
6	−1.38764	−0.43635	−0.02592
6	−4.29174	−2.53712	−0.27317
6	−2.93437	−2.10671	−0.10373
6	−5.32227	−1.60979	−0.58634
6	−4.57274	−3.88427	−0.14403
1	−5.59643	−4.22014	−0.26582
6	−6.24050	−0.88419	−0.89804
6	−7.30835	−0.00042	−1.23027
6	−9.40654	1.79079	−1.71286
6	−7.04810	1.33812	−1.53933
1	−6.02141	1.68887	−1.54976
6	−8.64290	−0.43809	−1.19776
1	−8.85662	−1.47715	−0.96965
6	−8.08356	2.23088	−1.78210
1	−7.84641	3.26712	−1.98613
6	−9.67875	0.44424	−1.43820
1	−10.71395	0.12166	−1.41141
6	−10.27190	3.96306	−2.09110
1	−9.72636	4.15657	−3.02297
1	−9.72186	4.40075	−1.24995
1	−11.26064	4.41713	−2.15430
7	2.00736	−1.89924	−0.12989
8	3.00702	−10.37330	−1.89538
6	1.07043	−0.98165	−0.02630
6	4.34145	−2.44682	−0.27560
6	3.29023	−1.48642	−0.10546
6	4.05369	−3.80295	−0.58887
6	5.64873	−2.01678	−0.14681
1	6.45134	−2.73543	−0.26912

6	3.88449	−4.96096	−0.90061
6	3.65282	−6.32763	−1.23267
6	3.15060	−9.04058	−1.71395
6	2.36325	−6.77182	−1.54032
1	1.54603	−6.05819	−1.55003
6	4.69935	−7.26431	−1.20135
1	5.70628	−6.92964	−0.97452
6	2.10774	−8.11507	−1.78229
1	1.09155	−8.42788	−1.98514
6	4.45313	−8.60266	−1.44121
1	5.25024	−9.33772	−1.41511
6	1.70214	−10.87701	−2.08931
1	1.26071	−10.50266	−3.02121
1	1.04879	−10.61853	−1.24790
1	1.80352	−11.96036	−2.15115
7	−0.61050	−2.69603	0.13147
8	3.28411	−10.28766	1.89527
6	−0.34378	−1.41203	0.02817
6	−2.24347	−4.45142	0.27660
6	−1.90575	−3.06804	0.10699
6	−1.24849	−5.41696	0.58920
6	−3.56860	−4.82273	0.14785
1	−3.83461	−5.86675	0.26976
6	−0.46193	−6.28379	0.90010
6	0.49228	−7.28930	1.23201
6	2.42194	−9.26130	1.71362
6	1.81049	−6.93893	1.53950
1	2.09098	−5.89085	1.54887
6	0.14579	−8.65040	1.20097
1	−0.87670	−8.93405	0.97426
6	2.77159	−7.91149	1.78152
1	3.78963	−7.60431	1.98373
6	1.09646	−9.62405	1.44099
1	0.84460	−10.67869	1.41516
6	4.64843	−9.97875	2.08848
1	4.80667	−9.42019	3.01929
1	5.04707	−9.40194	1.24583
1	5.16761	−10.93487	2.15220

3e ground state

7	−2.46651	−1.25119	−0.12983
6	−1.28676	−0.67833	−0.02779
6	−3.76754	−3.26517	−0.27303
6	−2.50947	−2.59797	−0.10416
6	−4.94229	−2.52977	−0.59022
6	−3.80465	−4.64011	−0.14300
1	−4.75154	−5.15401	−0.26467
6	−5.95966	−1.95536	−0.90684
6	−7.14181	−1.23453	−1.25078
6	−9.43475	0.21920	−1.76930
6	−7.05668	0.12339	−1.59012
1	−6.08547	0.60612	−1.60772
6	−8.39887	−1.85245	−1.19657
1	−8.46570	−2.90250	−0.93382
6	−8.20481	0.85467	−1.85280
1	−8.16022	1.91017	−2.09246
6	−9.55320	−1.12664	−1.45313
1	−10.53525	−1.58115	−1.39939
7	−0.11812	−2.76229	0.13021
6	−0.08544	−1.45139	0.02628
6	−1.41195	−4.78043	0.27786
6	−1.32616	−3.35926	0.10664
6	−0.25534	−5.54410	0.59487
6	−2.64805	−5.38411	0.15003
1	−2.72333	−6.45862	0.27344
6	0.69018	−6.23057	0.91105
6	1.83756	−7.00554	1.25495
6	4.11352	−8.48600	1.77266
6	3.03820	−6.36308	1.58972
1	3.07397	−5.27906	1.60409
6	1.79754	−8.40583	1.20460
1	0.86958	−8.90353	0.94519
6	4.18091	−7.10297	1.85190
1	5.12310	−6.62328	2.08790
6	2.93781	−9.15364	1.46080
1	2.93219	−10.23590	1.41012
7	−2.33444	1.48184	0.12819

6	−1.21543	0.79805	0.02547
6	−3.43534	3.61160	0.27311
6	−2.24739	2.82649	0.10342
6	−4.67542	2.99250	0.58996
6	−3.33986	4.98383	0.14441
1	−4.23282	5.58642	0.26683
6	−5.74338	2.51879	0.90662
6	−6.98989	1.91619	1.25078
6	−9.41335	0.69259	1.76997
6	−7.03723	0.55650	1.59041
1	−6.11761	−0.01844	1.60798
6	−8.18089	2.65344	1.19641
1	−8.14525	3.70493	0.93334
6	−8.25104	−0.05952	1.85352
1	−8.30936	−1.11431	2.09334
6	−9.40032	2.04342	1.45321
1	−10.33351	2.59127	1.39942
7	2.31457	−1.51034	−0.13181
6	1.22875	−0.77503	−0.02859
6	4.70897	−1.63016	−0.27842
6	3.50240	−0.87413	−0.10797
6	4.65904	−3.01532	−0.59515
6	5.91847	−0.97487	−0.15026
1	6.83676	−1.53800	−0.27317
6	4.67007	−4.18372	−0.91131
6	4.63703	−5.56814	−1.25435
6	4.52517	−8.28139	−1.76976
6	3.41809	−6.17456	−1.59020
1	2.51398	−5.57548	−1.60626
6	5.80132	−6.34701	−1.20181
1	6.74435	−5.87896	−0.94157
6	3.35914	−7.53483	−1.85131
1	2.42252	−8.02489	−2.08812
6	5.75021	−7.70991	−1.45676
1	6.63538	−8.33249	−1.40441
7	0.14862	2.75986	−0.13167
6	0.05481	1.45184	−0.02877
6	−0.94481	4.89347	−0.27671

6	−0.99628	3.47044	−0.10703
6	0.27992	5.54267	−0.59297
6	−2.11701	5.61324	−0.14806
1	−2.08848	6.69015	−0.27029
6	1.28688	6.13586	−0.90826
6	2.50286	6.79858	−1.25116
6	4.90931	8.05618	−1.76800
6	3.63608	6.04529	−1.58987
1	3.56792	4.96287	−1.60756
6	2.59706	8.19610	−1.19646
1	1.72129	8.77945	−0.93408
6	4.84399	6.67332	−1.85171
1	5.73565	6.10649	−2.09075
6	3.80331	8.83221	−1.45213
1	3.90132	9.90988	−1.39817
7	2.44945	1.27911	0.12725
6	1.29771	0.65190	0.02494
6	4.84435	1.16757	0.27183
6	3.57039	0.53136	0.10240
6	4.92846	2.55097	0.58906
6	5.98496	0.39879	0.14249
1	6.95337	0.87077	0.26455
6	5.05273	3.71261	0.90594
6	5.15447	5.09322	1.25083
6	5.30763	7.80345	1.77190
6	4.00071	5.81457	1.58985
1	3.04269	5.30613	1.60632
6	6.38882	5.75540	1.19786
1	7.28153	5.19848	0.93519
6	4.07468	7.17358	1.85379
1	3.19049	7.75184	2.09320
6	6.47081	7.11627	1.45564
1	7.41224	7.64996	1.40312
9	5.22354	−9.20189	2.00021
9	4.46554	−9.60106	−1.99631
9	5.37692	9.12216	2.00131
9	6.08242	8.66333	−1.99518
9	−10.58993	0.09266	1.99835

9	−10.54740	0.93085	−1.99730

3h ground state

7	2.72544	−0.38116	−0.14892
8	8.15637	−6.71073	−2.38623
6	1.42827	−0.18668	−0.04896
6	4.93745	0.54523	−0.28004
6	3.52184	0.70513	−0.11402
6	5.48517	−0.72220	−0.61782
6	5.74321	1.65822	−0.13276
1	6.81536	1.54804	−0.25066
6	5.98287	−1.77133	−0.95974
6	6.54200	−3.02144	−1.36039
6	7.61431	−5.50752	−2.04494
6	5.68618	−4.07355	−1.72007
1	4.61622	−3.91075	−1.68454
6	7.92504	−3.19985	−1.37785
1	8.59674	−2.39659	−1.10153
6	6.21932	−5.30964	−2.06529
6	8.45389	−4.43900	−1.72299
6	7.96387	−7.74272	−1.42207
1	6.90060	−7.93329	−1.25418
1	8.43442	−7.48759	−0.46761
1	8.43604	−8.63524	−1.83452
7	1.64134	2.19072	0.12333
8	0.48239	10.33241	2.35748
6	0.87405	1.12870	0.00925
6	3.84671	3.12294	0.29609
6	2.97497	1.99997	0.10583
6	3.30608	4.39289	0.63476
6	5.20805	2.92303	0.17326
1	5.87692	3.76533	0.31018
6	2.85557	5.46335	0.97480
6	2.26339	6.70002	1.36791
6	1.05218	9.13424	2.03167
6	0.88328	6.75813	1.59355
1	0.26436	5.87599	1.47300
6	3.03992	7.85487	1.50689

1	4.10870	7.83201	1.33127
6	0.28364	7.96614	1.91632
6	2.43870	9.06116	1.84018
6	−0.31419	10.90820	1.32179
1	−1.11989	10.23164	1.02420
1	0.29298	11.14606	0.44402
1	−0.73284	11.82618	1.73603
7	1.07206	−2.55959	0.10227
8	8.69721	−5.72007	2.31339
6	0.53530	−1.36350	0.00043
6	0.77760	−4.93700	0.24928
6	0.23994	−3.61891	0.07661
6	2.15197	−5.10390	0.57321
6	−0.07474	−6.01624	0.11859
1	0.32129	−7.01783	0.24457
6	3.31217	−5.24374	0.89025
6	4.68580	−5.36747	1.25620
6	7.39093	−5.58395	1.94407
6	5.43311	−4.21658	1.53022
1	4.98630	−3.23201	1.44891
6	5.29028	−6.62654	1.33955
1	4.73095	−7.53032	1.12811
6	6.77315	−4.32556	1.87066
6	6.63157	−6.73323	1.68177
6	9.64915	−5.23391	1.36530
1	9.46457	−4.18358	1.12610
1	9.61908	−5.81990	0.44349
1	10.62646	−5.34327	1.83673
7	−1.07163	2.53331	−0.15550
8	2.03840	10.18522	−2.28868
6	−0.59148	1.31341	−0.04974
6	−2.97870	3.98473	−0.30887
6	−2.41082	2.67949	−0.13121
6	−2.14284	5.08426	−0.64326
6	−4.34619	4.12798	−0.17458
1	−4.78579	5.11095	−0.30147
6	−1.44419	6.01468	−0.97592
6	−0.56988	7.07560	−1.35598

6	1.19487	9.15473	−1.98386
6	0.78280	6.80101	−1.58799
1	1.16952	5.79321	−1.48542
6	−1.04353	8.38634	−1.47252
1	−2.08580	8.62011	−1.29175
6	1.65732	7.83305	−1.89361
6	−0.16801	9.41654	−1.78800
6	2.95077	10.52862	−1.24530
1	3.56868	9.67078	−0.96637
1	2.41932	10.88717	−0.35948
1	3.57924	11.32663	−1.64240
7	−1.69820	−2.21146	−0.15921
8	−9.86977	−3.33692	−2.32008
6	−0.88210	−1.18555	−0.05325
6	−2.00021	−4.58977	−0.31214
6	−1.15449	−3.44422	−0.13627
6	−3.37087	−4.41661	−0.64420
6	−1.43925	−5.84562	−0.18065
1	−2.07050	−6.71822	−0.30538
6	−4.52559	−4.27704	−0.97839
6	−5.87962	−4.04817	−1.36376
6	−8.55817	−3.55368	−2.00684
6	−6.31348	−2.73876	−1.60070
1	−5.63225	−1.90157	−1.49683
6	−6.78000	−5.11161	−1.48302
1	−6.46511	−6.13170	−1.29883
6	−7.64209	−2.49469	−1.91417
6	−8.10756	−4.86581	−1.80644
6	−10.62607	−2.69647	−1.29159
1	−10.18241	−1.73494	−1.01933
1	−10.68824	−3.32551	−0.39938
1	−11.62520	−2.54335	−1.70109
7	−2.75889	0.30958	0.11024
8	−9.27952	−4.73115	2.32127
6	−1.45476	0.17606	0.00395
6	−4.67000	1.75547	0.26618
6	−3.26032	1.55997	0.08661
6	−5.50286	0.65524	0.60617

6	−5.17642	3.03374	0.13173
1	−6.24124	3.19263	0.26010
6	−6.20915	−0.26603	0.94812
6	−6.99293	−1.39079	1.34191
6	−8.51601	−3.64434	2.00156
6	−6.36335	−2.61864	1.57626
1	−5.28977	−2.72152	1.46412
6	−8.38143	−1.28538	1.47225
1	−8.88819	−0.34519	1.29080
6	−7.11970	−3.73573	1.89755
6	−9.13568	−2.40279	1.80391
6	−9.37225	−5.71173	1.28736
1	−8.38145	−6.07505	1.00143
1	−9.87106	−5.30572	0.40301
1	−9.96501	−6.53007	1.69773
8	7.20750	−7.97353	1.69565
8	7.49308	−3.18221	2.08560
8	5.48883	−6.38945	−2.42931
8	9.81220	−4.60280	−1.68003
8	−8.97817	−5.92079	−1.84699
8	−8.05588	−1.19958	−2.06752
8	−6.48624	−4.93944	2.04626
8	−10.49795	−2.28252	1.85410
8	−0.64879	10.69737	−1.82470
8	2.98740	7.54799	−2.04006
8	3.21562	10.18628	1.89988
8	−1.07680	8.01090	2.05615
6	−1.52507	8.25118	3.38332
1	−2.61513	8.23634	3.34642
1	−1.17536	7.46066	4.05809
1	−1.18135	9.22334	3.75030
6	3.33047	10.76778	3.19200
1	4.00269	11.62042	3.08529
1	2.35891	11.10776	3.56047
1	3.76481	10.05175	3.90037
6	3.48376	7.71141	−3.36207
1	4.53723	7.43039	−3.33100
1	2.95355	7.05011	−4.05781

1	3.38750	8.74854	−3.69837
6	−0.62035	11.31214	−3.10613
1	−1.06731	12.29958	−2.98183
1	0.40438	11.41479	−3.47319
1	−1.21451	10.73450	−3.82477
6	−9.51824	−6.20392	−3.13117
1	−10.15196	−7.08358	−3.00943
1	−10.11618	−5.36726	−3.50235
1	−8.71735	−6.43163	−3.84519
6	−8.42824	−0.85297	−3.39485
1	−8.70779	0.20104	−3.36963
1	−7.58284	−0.98661	−4.08031
1	−9.27679	−1.45197	−3.74007
6	−11.06967	−2.46950	3.14220
1	−10.66630	−1.73828	3.85332
1	−12.14215	−2.30553	3.02768
1	−10.88882	−3.48225	3.51232
6	−6.49813	−5.45281	3.37179
1	−5.94214	−6.39078	3.34230
1	−5.99987	−4.75859	4.05900
1	−7.51961	−5.63939	3.71774
6	10.42630	−4.84588	−2.93921
1	10.23096	−4.01648	−3.62988
1	11.49875	−4.90998	−2.74877
1	10.07160	−5.78210	−3.37789
6	4.08986	−6.22879	−2.55739
1	3.71198	−7.18775	−2.91216
1	3.62002	−5.98259	−1.59948
1	3.84621	−5.45054	−3.29074
6	7.61380	−8.43235	2.97856
1	6.75554	−8.48341	3.65983
1	8.01602	−9.43562	2.83117
1	8.38461	−7.78450	3.40441
6	7.88181	−2.97898	3.43784
1	8.42352	−2.03263	3.46239
1	7.00038	−2.90858	4.08638
1	8.53172	−3.78573	3.79134

7. Flipping energy of 3g calculated by NEB method

The flip energy of 3g was calculated using the nudge elastic band (NEB) method[Ref)]. Gaussian 16 program was used for the calculation, with the level as ωB97XD/6-31G(d,p). The Reaction Plus software (HPC System Inc.)[4] was used for the NEB method, and the coordinates near the vertex of the flip motion were extracted. While the energy calculated from this NEB method is not what is commonly known as transition state, however a value close to it is obtained. The actual flip energy is thought to be slightly smaller than this.

As a result, there are 1.2 kcal/mol gradient between the ground state (propeller form) and the flipped form, and that the transition requires 28.1 kcal/mol. Considering that the true flip energy is smaller than that calculated by the NEB method, flipping at room temperature cannot be completely ruled out.

Cartesian coordinates

3g ground state (propeller form)

Atomic number	x	y	z
7	−1.69030	−2.28483	−0.15400
6	−0.91650	−1.22866	−0.03575
6	−1.90754	−4.67359	−0.27814
6	−1.10507	−3.49741	−0.09940
6	−3.27874	−4.53440	−0.62431
6	−1.30750	−5.90780	−0.12748
1	−1.90612	−6.80259	−0.25620
6	−4.43063	−4.36816	−0.95310
6	−5.77680	−4.06392	−1.32099
6	−8.41117	−3.36485	−1.89816
6	−6.06013	−2.78270	−1.79871
1	−5.27918	−2.03824	−1.89073
6	−6.79406	−5.00488	−1.13875
1	−6.58188	−5.99009	−0.74338
6	−7.37941	−2.44063	−2.07983
6	−8.10591	−4.63793	−1.41342
7	1.07944	−2.53189	0.19702
6	0.50568	−1.35572	0.07142
6	0.86819	−4.92054	0.33672
6	0.28788	−3.62147	0.14988
6	2.24272	−5.02541	0.68182
6	0.05784	−6.02919	0.19428
1	0.48776	−7.01534	0.32927

6	3.40644	-5.06443	1.00861
6	4.78600	-4.99889	1.37277
6	7.50389	-4.76738	1.93927
6	5.62124	-6.10629	1.20120
1	5.23804	-7.04339	0.81781
6	5.29159	-3.78212	1.83533
1	4.65386	-2.91094	1.91881
6	6.97795	-5.97266	1.47073
6	6.65126	-3.67415	2.11114
7	-2.85600	0.20996	0.00515
6	-1.54308	0.11155	-0.02931
6	-4.81142	1.62154	0.01880
6	-3.39041	1.44572	-0.07982
6	-5.67158	0.53709	0.34828
6	-5.33219	2.88552	-0.18605
1	-6.40560	3.02254	-0.11707
6	-6.46626	-0.31683	0.66972
6	-7.39353	-1.34116	1.03421
6	-9.20177	-3.37792	1.65092
6	-6.91720	-2.58282	1.45520
1	-5.85444	-2.78339	1.50816
6	-8.76868	-1.10254	0.93191
1	-9.14693	-0.13813	0.61589
6	-7.82395	-3.59643	1.75295
6	-9.66014	-2.12541	1.23539
7	2.67047	-0.28719	0.02046
6	1.36171	-0.14867	0.05747
6	4.84659	0.74577	0.00259
6	3.41779	0.83279	0.10293
6	5.49453	-0.47659	-0.32894
6	5.58988	1.89206	0.21185
1	6.67042	1.83165	0.14485
6	6.12160	-1.45978	-0.65163
6	6.85299	-2.63346	-1.01133
6	8.27441	-4.96058	-1.61303
6	8.24913	-2.63806	-0.91531
1	8.79163	-1.75191	-0.60962
6	6.16496	-3.77615	-1.41939

1	5.08328	−3.78857	−1.46701
6	8.94669	−3.80381	−1.21119
6	6.87920	−4.93542	−1.70946
7	−1.21965	2.47154	−0.23483
6	−0.71215	1.27195	−0.06387
6	−3.14258	3.86864	−0.55172
6	−2.56036	2.58051	−0.29102
6	−2.26305	4.93053	−0.89330
6	−4.51423	3.99029	−0.49174
1	−4.96970	4.95589	−0.68047
6	−1.37464	5.68673	−1.21312
6	−0.17951	6.39371	−1.54848
6	2.26762	7.64736	−1.99742
6	0.96031	5.61915	−1.77697
1	0.91880	4.53984	−1.69818
6	−0.11466	7.78759	−1.55852
1	−0.98733	8.39401	−1.35172
6	2.17665	6.25246	−1.99826
6	1.11444	8.40427	−1.76819
7	1.46645	2.23367	0.25302
6	0.75261	1.14240	0.08615
6	3.61240	3.25656	0.57924
6	2.80530	2.09955	0.31309
6	2.98140	4.48351	0.91720
6	4.98468	3.12373	0.52307
1	5.60698	3.98944	0.72162
6	2.38820	5.49443	1.21623
6	1.58808	6.64592	1.48909
6	−0.05290	8.87656	1.81321
6	0.22506	6.46789	1.73212
1	−0.21482	5.47839	1.73117
6	2.14342	7.92647	1.42774
1	3.19611	8.07112	1.21876
6	−0.58662	7.58684	1.89171
6	1.31506	9.03245	1.57325
8	−10.98044	−1.82679	1.07414
8	−7.28035	−4.77825	2.12736
8	−7.58248	−1.16976	−2.50375

8	−9.05363	−5.58448	−1.15408
8	1.10566	9.76446	−1.71420
8	3.24070	5.43043	−2.19013
8	−1.90205	7.33066	2.10260
8	1.92307	10.24450	1.45421
8	6.13571	−6.00724	−2.07088
8	10.29946	−3.73892	−1.05669
8	7.07595	−2.45388	2.51967
8	7.74355	−7.07444	1.22333
6	9.13549	−6.96460	1.41042
1	9.55984	−7.92080	1.10226
1	9.56011	−6.16077	0.79557
1	9.38991	−6.78274	2.46213
6	8.45823	−2.25752	2.70982
1	8.57964	−1.20552	2.96847
1	8.84615	−2.87430	3.53114
1	9.02415	−2.47363	1.79527
6	11.07904	−4.79656	−1.56596
1	12.11912	−4.49213	−1.44684
1	10.91945	−5.73150	−1.01255
1	10.87223	−4.97404	−2.62842
6	6.76637	−7.26913	−2.12419
1	5.97459	−7.99091	−2.32572
1	7.50453	−7.32040	−2.93532
1	7.24588	−7.51010	−1.16784
6	2.34292	10.43849	−1.77646
1	2.11077	11.50063	−1.68881
1	2.99528	10.14595	−0.94538
1	2.85245	10.26565	−2.73261
6	4.52940	5.95696	−1.98018
1	5.21076	5.10555	−2.00633
1	4.81946	6.66707	−2.76569
1	4.59956	6.44713	−1.00056
6	1.10093	11.38226	1.31720
1	0.53542	11.59050	2.23423
1	1.77340	12.21890	1.12363
1	0.40918	11.26595	0.47405
6	−2.79796	8.41535	2.13234

1	−3.79161	7.98346	2.25243
1	−2.59737	9.08855	2.97605
1	−2.76639	8.99097	1.19818
6	−10.40457	−5.23358	−1.34489
1	−10.62382	−5.02372	−2.39930
1	−10.99055	−6.09596	−1.02500
1	−10.68058	−4.35955	−0.74126
6	−8.90865	−0.73530	−2.69875
1	−9.50417	−0.83991	−1.78344
1	−8.84313	0.31912	−2.96746
1	−9.39851	−1.28218	−3.51511
6	−11.93398	−2.72432	1.59466
1	−11.76154	−2.92207	2.65967
1	−12.90433	−2.24326	1.46940
1	−11.94136	−3.67964	1.05324
6	−8.12281	−5.90905	2.19414
1	−7.47027	−6.75584	2.40784
1	−8.85970	−5.81928	3.00309
1	−8.63584	−6.07487	1.23920
1	−9.89892	−4.17400	1.87197
1	−9.43508	−3.08261	−2.09416
1	8.82030	−5.86835	−1.82883
1	8.56220	−4.66761	2.13036
1	−0.69145	9.74191	1.91965
1	3.22285	8.13091	−2.14652

3g flipped form

7	0.47294	−2.48120	0.46164
6	0.18441	−1.22321	0.21574
6	−0.27259	−4.70796	0.93866
6	−0.55511	−3.33137	0.65379
6	1.07519	−5.14675	1.05534
6	−1.33338	−5.56860	1.13904
1	−1.13169	−6.61258	1.35103
6	2.21962	−5.50736	1.20672
6	3.59244	−5.86308	1.38314
6	6.29527	−6.49444	1.63765
6	4.49041	−4.88594	1.81678

1	4.16159	−3.87206	2.00741
6	4.02943	−7.15641	1.08181
1	3.34119	−7.91160	0.72401
6	5.83955	−5.20798	1.93516
6	5.38107	−7.45477	1.20105
7	−2.18359	−1.58384	0.33138
6	−1.17106	−0.76960	0.12875
6	−2.97305	−3.78549	0.88092
6	−1.90108	−2.87280	0.61012
6	−4.32198	−3.33915	0.96461
6	−2.66325	−5.11181	1.11552
1	−3.46916	−5.80945	1.31394
6	−5.48232	−3.03696	1.12742
6	−6.85019	−2.67528	1.32793
6	−9.53223	−1.96650	1.65205
6	−7.19158	−1.33692	1.52431
1	−6.43612	−0.56115	1.51437
6	−7.83717	−3.66544	1.30095
1	−7.58070	−4.70570	1.14410
6	−8.53096	−0.98966	1.67938
6	−9.16922	−3.30095	1.45606
7	2.53837	−0.68809	0.09229
6	1.29664	−0.25326	0.10725
6	4.89237	−0.17600	0.08638
6	3.52003	0.23338	0.17388
6	5.23655	−1.50673	−0.28008
6	5.87367	0.76563	0.33172
1	6.91321	0.46212	0.27775
6	5.58882	−2.60288	−0.65209
6	5.98347	−3.90305	−1.09395
6	6.74168	−6.47157	−1.88661
6	5.00952	−4.81624	−1.49864
1	3.95850	−4.55688	−1.47534
6	7.33632	−4.25829	−1.09183
1	8.09880	−3.55326	−0.78470
6	5.39355	−6.09684	−1.88688
6	7.70239	−5.54149	−1.48182
7	−2.68159	1.00528	−0.48799

6	−1.45000	0.64745	−0.19567
6	−4.21999	2.73002	−1.14980
6	−2.89729	2.30048	−0.79697
6	−5.27811	1.78451	−1.24499
6	−4.42262	4.06455	−1.44394
1	−5.42127	4.39618	−1.70548
6	−6.17388	0.98582	−1.39592
6	−7.19690	0.00352	−1.56823
6	−9.17658	−1.94498	−1.81515
6	−6.83173	−1.32921	−1.76492
1	−5.79023	−1.62600	−1.77442
6	−8.54328	0.37502	−1.50215
1	−8.83285	1.40438	−1.33285
6	−7.82586	−2.29746	−1.87905
6	−9.51967	−0.60595	−1.61845
7	1.93993	2.04432	0.27147
6	1.00128	1.14375	0.08461
6	4.25242	2.54166	0.69156
6	3.20891	1.60533	0.38270
6	3.90335	3.87345	1.04043
6	5.55850	2.09717	0.66272
1	6.35762	2.79300	0.89382
6	3.52334	4.98102	1.34428
6	2.96578	6.26783	1.61523
6	1.81234	8.78563	1.93421
6	1.57751	6.38198	1.70630
1	0.94022	5.51418	1.58872
6	3.78509	7.39645	1.70206
1	4.86128	7.31604	1.61173
6	1.01038	7.64296	1.86419
6	3.20025	8.64865	1.84414
7	−0.57426	2.85648	−0.49516
6	−0.37837	1.59219	−0.19668
6	−2.06878	4.60531	−1.17353
6	−1.82912	3.23763	−0.80353
6	−0.94481	5.46639	−1.29762
6	−3.36189	4.99083	−1.45835
1	−3.55787	6.02086	−1.73438

6	0.11190	6.03671	−1.44388
6	1.45704	6.49543	−1.59252
6	4.14095	7.23540	−1.70800
6	2.43696	5.51407	−1.75832
1	2.17145	4.46434	−1.76380
6	1.80134	7.84460	−1.49784
1	1.05045	8.60825	−1.33927
6	3.77274	5.89151	−1.81504
6	3.14491	8.20248	−1.54077
8	9.03540	−5.81907	−1.41354
8	4.38571	−6.92582	−2.24626
8	6.65574	−4.19952	2.32664
8	5.74228	−8.72142	0.84414
8	4.06135	9.70281	1.87431
8	−0.34463	7.67266	1.92076
8	4.66397	4.87714	−1.96027
8	3.40494	9.53057	−1.39371
8	−10.80880	−0.17200	−1.50237
8	−7.39300	−3.57185	−2.03337
8	−10.06937	−4.32254	1.37429
8	−8.77749	0.33112	1.84677
6	−10.11684	0.77293	1.80886
1	−10.07550	1.85928	1.88997
1	−10.59989	0.49953	0.86300
1	−10.69831	0.37786	2.65232
6	−11.41891	−4.04685	1.66442
1	−11.94119	−5.00286	1.62100
1	−11.53743	−3.61521	2.66591
1	−11.86661	−3.36478	0.92927
6	−8.35216	−4.60496	−2.06044
1	−7.78870	−5.53468	−2.14109
1	−8.95092	−4.62516	−1.14156
1	−9.01766	−4.51530	−2.92909
6	−11.83898	−1.12742	−1.58697
1	−12.77320	−0.58018	−1.45847
1	−11.84836	−1.63047	−2.56191
1	−11.75127	−1.88405	−0.79614
6	3.51950	10.99794	1.73893

1	4.37110	11.67571	1.66784
1	2.91235	11.07493	0.82857
1	2.91599	11.28131	2.61048
6	−0.98568	8.92524	1.94366
1	−2.05563	8.71750	1.92969
1	−0.74375	9.49169	2.85244
1	−0.72477	9.52854	1.06448
6	4.75311	9.93651	−1.31135
1	5.30468	9.70269	−2.23049
1	4.73299	11.01899	−1.17871
1	5.25241	9.48082	−0.44820
6	6.00592	5.12069	−1.61219
1	6.50508	4.15122	−1.64039
1	6.50198	5.79477	−2.32279
1	6.08095	5.53792	−0.59972
6	7.10820	−9.05912	0.90293
1	7.49505	−9.00871	1.92840
1	7.18334	−10.08595	0.54368
1	7.71049	−8.40336	0.26105
6	8.04085	−4.45115	2.39687
1	8.44244	−4.77089	1.42743
1	8.50342	−3.50645	2.68316
1	8.27588	−5.20861	3.15607
6	9.49706	−7.01807	−1.99072
1	9.20073	−7.09907	−3.04373
1	10.58486	−6.98873	−1.92384
1	9.13407	−7.90319	−1.45146
6	4.67762	−8.29406	−2.43225
1	3.72165	−8.77928	−2.62997
1	5.33939	−8.45467	−3.29349
1	5.12570	−8.72786	−1.53041
1	7.03078	−7.47168	−2.17806
1	7.34705	−6.72942	1.70862
1	−9.94135	−2.70509	−1.88245
1	−10.57103	−1.68632	1.75740
1	5.18392	7.51878	−1.72737
1	1.36532	9.76385	2.03905

3g near-transition structure of flip (obtained by NEB method)

7	−0.59710	−2.53070	−0.50130
6	−0.26490	−1.27960	−0.29140
6	0.05900	−4.77500	−0.98790
6	0.39630	−3.41200	−0.71130
6	−1.29670	−5.16150	−1.09910
6	1.06520	−5.69740	−1.18030
1	0.81540	−6.73210	−1.38380
6	−2.43450	−5.53480	−1.24560
6	−3.80230	−5.88970	−1.40760
6	−6.50060	−6.51700	−1.66710
6	−4.67820	−4.94260	−1.93770
1	−4.33340	−3.95980	−2.23320
6	−4.25400	−7.14860	−1.01090
1	−3.57910	−7.88180	−0.58800
6	−6.02420	−5.26010	−2.05380
6	−5.60650	−7.44300	−1.12660
7	2.08910	−1.70380	−0.46690
6	1.10720	−0.84230	−0.29960
6	2.75850	−3.99140	−0.86390
6	1.75180	−3.00080	−0.66880
6	4.14150	−3.72630	−0.75290
6	2.40390	−5.30330	−1.11410
1	3.19540	−6.03040	−1.25550
6	5.33460	−3.84520	−0.62380
6	6.70240	−3.93920	−0.26320
6	9.33910	−4.06690	0.55090
6	7.65110	−4.48340	−1.13370
1	7.38930	−4.85320	−2.12010
6	7.03790	−3.48000	1.01180
1	6.29390	−3.06780	1.68660
6	8.97430	−4.52860	−0.71420
6	8.36210	−3.54820	1.40510
7	−2.60490	−0.76460	0.02310
6	−1.37260	−0.31960	−0.11350
6	−4.95330	−0.25080	0.22850
6	−3.59310	0.15330	0.02330
6	−5.29360	−1.58600	0.57340

6	−5.95330	0.69330	0.09300
1	−6.98210	0.38550	0.24300
6	−5.70360	−2.67770	0.89190
6	−6.16910	−3.97810	1.24930
6	−7.06590	−6.53160	1.91710
6	−5.26650	−4.91080	1.75930
1	−4.22010	−4.66570	1.89620
6	−7.51970	−4.30470	1.07640
1	−8.22710	−3.58330	0.68650
6	−5.72170	−6.18510	2.08080
6	−7.95270	−5.58310	1.40250
7	2.65820	1.02740	−0.06070
6	1.40960	0.60760	−0.12950
6	4.18440	2.90170	0.18600
6	2.86230	2.35590	0.11560
6	5.36460	2.11900	0.09190
6	4.35400	4.26250	0.37050
1	5.36200	4.65760	0.40650
6	6.49240	1.68920	0.05010
6	7.81370	1.15400	0.05840
6	10.33510	−0.02250	0.14750
6	8.56350	1.23210	1.23410
1	8.18420	1.71310	2.12880
6	8.32620	0.54280	−1.08700
1	7.75140	0.50720	−2.00510
6	9.81780	0.64050	1.26650
6	9.58520	−0.04890	−1.03200
7	−2.03750	1.96940	−0.24710
6	−1.08380	1.07050	−0.14980
6	−4.37550	2.46470	−0.40680
6	−3.30740	1.52610	−0.21590
6	−4.05750	3.79620	−0.77500
6	−5.67340	2.02870	−0.24740
1	−6.48960	2.72950	−0.38280
6	−3.71510	4.90360	−1.11580
6	−3.21030	6.19090	−1.46340
6	−2.15300	8.70940	−2.02420
6	−1.85570	6.31260	−1.77680

1	−1.19780	5.45280	−1.75940
6	−4.04220	7.30980	−1.43740
1	−5.09100	7.22340	−1.18480
6	−1.33780	7.57200	−2.04530
6	−3.50540	8.56340	−1.70460
7	0.50040	2.81130	0.15500
6	0.31430	1.52960	−0.04850
6	1.98510	4.63850	0.50660
6	1.76760	3.24680	0.25330
6	0.86930	5.45730	0.77340
6	3.26870	5.12990	0.53800
1	3.43160	6.18710	0.71500
6	−0.11360	6.08560	1.07480
6	−1.35150	6.68630	1.41520
6	−3.84640	7.78380	1.99570
6	−2.40310	5.84600	1.76850
1	−2.29520	4.76900	1.77920
6	−1.52250	8.06540	1.35000
1	−0.71350	8.72100	1.05410
6	−3.64200	6.39910	2.04420
6	−2.77120	8.60140	1.62830
8	−9.26890	−5.84780	1.16280
8	−4.78180	−7.03970	2.54680
8	−6.81220	−4.26060	−2.52240
8	−5.97970	−8.66600	−0.65520
8	−4.38360	9.59810	−1.61260
8	−0.00260	7.60810	−2.28810
8	−4.60250	5.48530	2.32290
8	−2.84670	9.95180	1.49490
8	10.00570	−0.63940	−2.17830
8	10.48160	0.75190	2.44590
8	8.63980	−3.08730	2.64690
8	9.87450	−5.03460	−1.59570
6	11.21430	−5.06980	−1.17430
1	11.77620	−5.55010	−1.97400
1	11.59300	−4.05460	−1.00080
1	11.32630	−5.63770	−0.24290
6	9.99190	−3.14650	3.00450

1	10.08060	−2.70730	3.99740
1	10.36100	−4.17950	2.98930
1	10.57450	−2.57680	2.27610
6	11.63790	−0.03000	2.58860
1	12.00160	0.13210	3.60220
1	11.39220	−1.08400	2.45440
1	12.40530	0.23930	1.85420
6	11.27120	−1.25180	−2.20700
1	11.36140	−1.70040	−3.19650
1	12.08230	−0.52670	−2.05880
1	11.35510	−2.04070	−1.44970
6	−3.91310	10.89700	−1.88350
1	−4.76830	11.56040	−1.75000
1	−3.11720	11.18730	−1.18710
1	−3.55010	10.98580	−2.91550
6	0.52660	8.76740	−2.88040
1	1.58200	8.56280	−3.05810
1	0.02990	8.98020	−3.83530
1	0.44180	9.64250	−2.22430
6	−4.04060	10.58080	1.88600
1	−4.27140	10.36000	2.93510
1	−3.87040	11.65230	1.77650
1	−4.87660	10.27460	1.24630
6	−5.80100	5.95110	2.87610
1	−6.42570	5.07250	3.02520
1	−5.61330	6.44580	3.83700
1	−6.31550	6.63920	2.19700
6	−7.30790	−9.08010	−0.86140
1	−7.53750	−9.17260	−1.93050
1	−7.39510	−10.05980	−0.38980
1	−8.02510	−8.39130	−0.39750
6	−8.09990	−4.58870	−2.98200
1	−8.66810	−5.15860	−2.24060
1	−8.61240	−3.64300	−3.15400
1	−8.04250	−5.15500	−3.92050
6	−9.84080	−6.97060	1.79290
1	−9.68260	−6.94240	2.87820
1	−10.90880	−6.92540	1.57810

1	−9.43970	−7.91530	1.40260
6	−5.21280	−8.31050	2.97140
1	−4.32670	−8.81850	3.35020
1	−5.95450	−8.22260	3.77610
1	−5.63620	−8.88350	2.13790
1	−7.40660	−7.53000	2.15100
1	−7.55250	−6.75140	−1.73500
1	11.30130	−0.50460	0.19030
1	10.36850	−4.10940	0.86560
1	−4.82030	8.21180	2.19130
1	−1.73420	9.68880	−2.20800

8. Crystal data

3c (CCDC 2488187)

Formula	$C_{84}H_{60}Cl_{12}N_6O_{12}$
$D_{calc.}$/ g cm^{-3}	1.359
m/mm^{-1}	4.030
Formula Weight	1770.78
Colour	dark red
Shape	prism
Size/mm^3	0.45×0.35×0.20
T/K	100.01(10)
Crystal System	trigonal
Space Group	*R*-3*c*
a/Å	23.2705(3)
b/Å	23.2705(3)
c/Å	27.6726(4)
a/°	90
b/°	90
g/°	120
V/Å^3	12977.5(4)
Z	6
Z'	0.166667
Wavelength/Å	1.54184
Radiation type	Cu K_a
Q_{min}/°	3.799
Q_{max}/°	75.402
Measured Refl.	29574
Independent Refl.	2928
Reflections with I > 2(I)	2694
R_{int}	0.0658
Parameters	173
Restraints	0
Largest Peak	1.258
Deepest Hole	-0.461
GooF	1.120
wR_2 (all data)	0.2271
wR_2	0.2228
R_1 (all data)	0.0750
R_1	0.0721

3m (CCDC 2487906)

Formula	$C_{117}H_{101}Cl_{12}N_{12}$
$D_{calc.}$/ g cm^{-3}	1.405
m/mm^{-1}	3.527
Formula Weight	2100.49
Colour	dark orange
Shape	prism
Size/mm^3	0.39×0.29×0.15
T/K	100.00(10)
Crystal System	trigonal
Space Group	R-3c
a/Å	25.2385(2)
b/Å	25.2385(2)
c/Å	26.9958(3)
a/°	90
b/°	90
g/°	120
V/Å^3	14892.0(3)
Z	6
Z'	0.166667
Wavelength/Å	1.54184
Radiation type	Cu K_a
Q_{min}/°	3.502
Q_{max}/°	75.475
Measured Refl.	33695
Independent Refl.	3407
Reflections with I > 2(I)	3183
R_{int}	0.0291
Parameters	191
Restraints	0
Largest Peak	1.589
Deepest Hole	-0.855
GooF	1.116
wR_2 (all data)	0.2577
wR_2	0.2551
R_1 (all data)	0.0834
R_1	0.0815

9. References

[1] E.Rasolofonjatovo, O. Provot, A.Hamze, J. Bignon, S. Thoret, J. -D. Brion, M. Alami *Eur. J. Med. Chem.* **2010**, *45*, 3617–3626.

[2] J. Hu, A. Paudel, T. Yamato, *J. Chem. Res.* **2009**, 109-113.

[3] R. Liu, C. Malotki, L. Arnold, N. Koshino, H. Higashimura, M. Baumgarten, K. Müllen, *J. Am. Chem. Soc.* **2011**, *133*, 10372-10375.

[4] N. Sugimura, Y. Igarashi, R. Aoyama T. Shibue, *Eur. J. Mass Spectrom.* **2017**, *23*, 40–44.

10. ^{1}H- and ^{13}C-NMR spectra

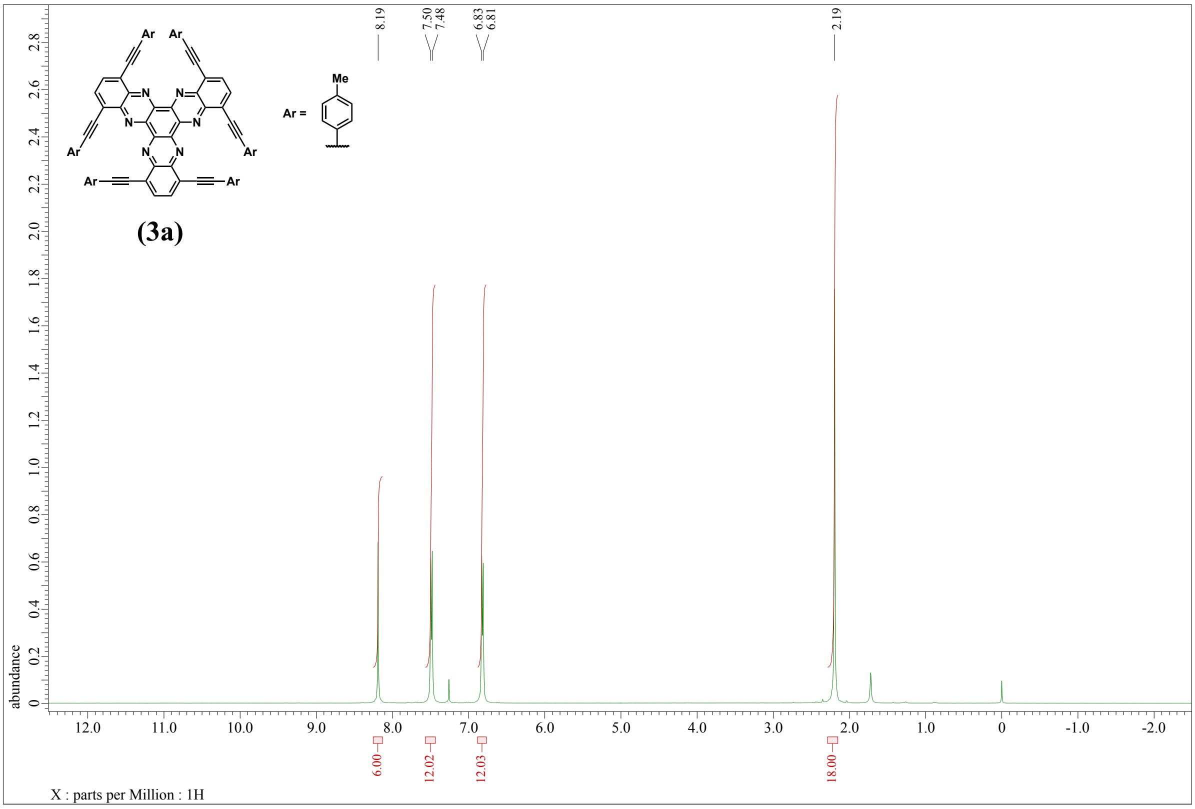

400 MHz

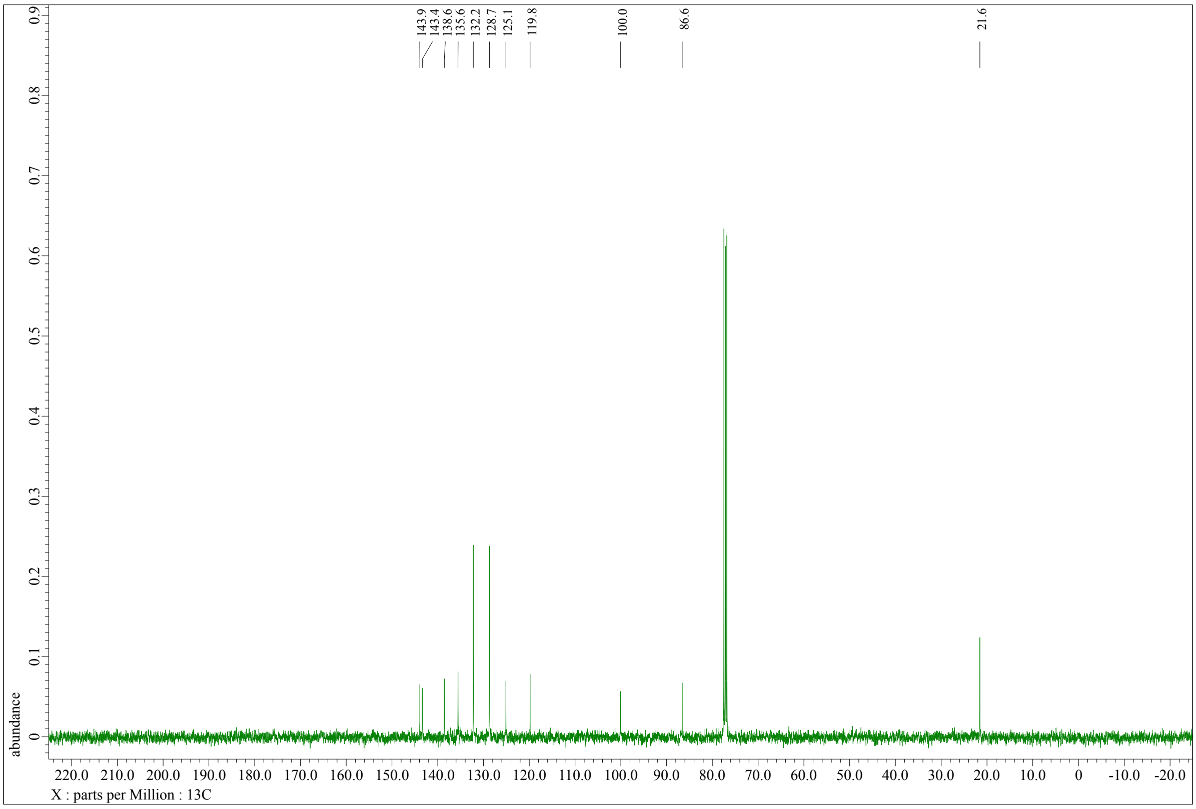

101 MHz

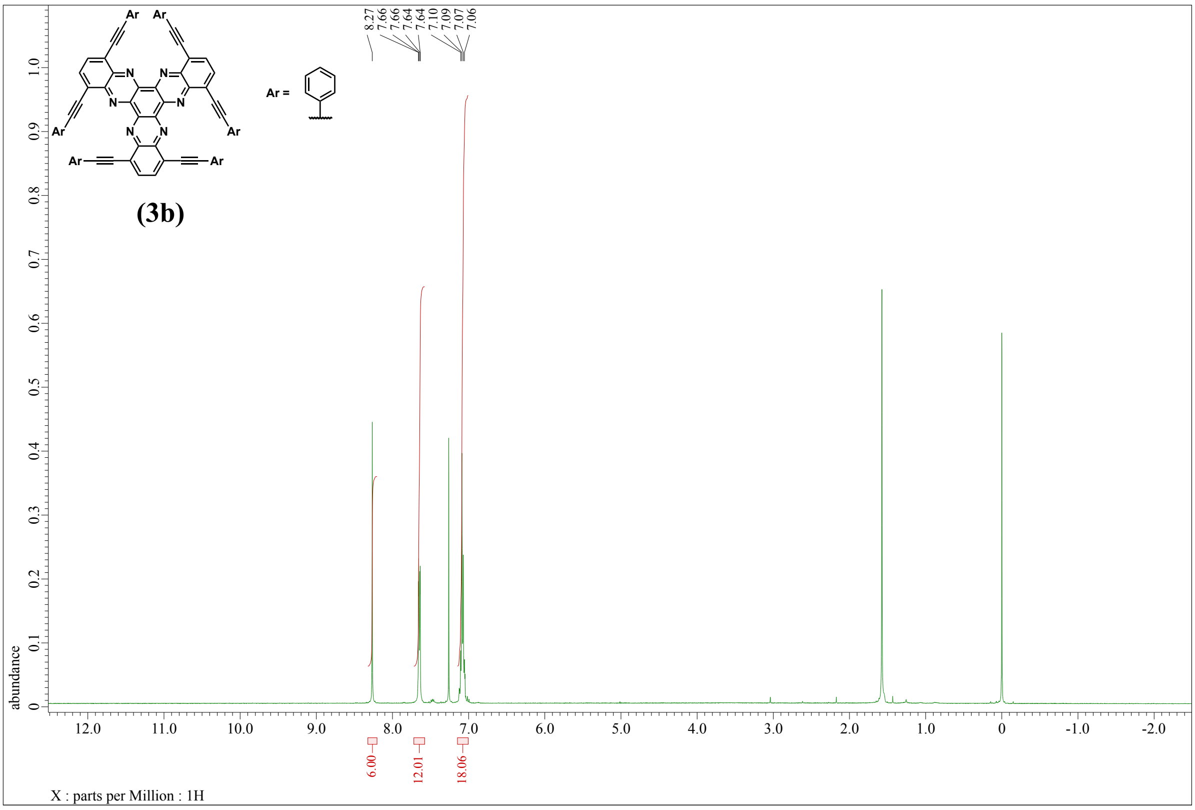

400 MHz

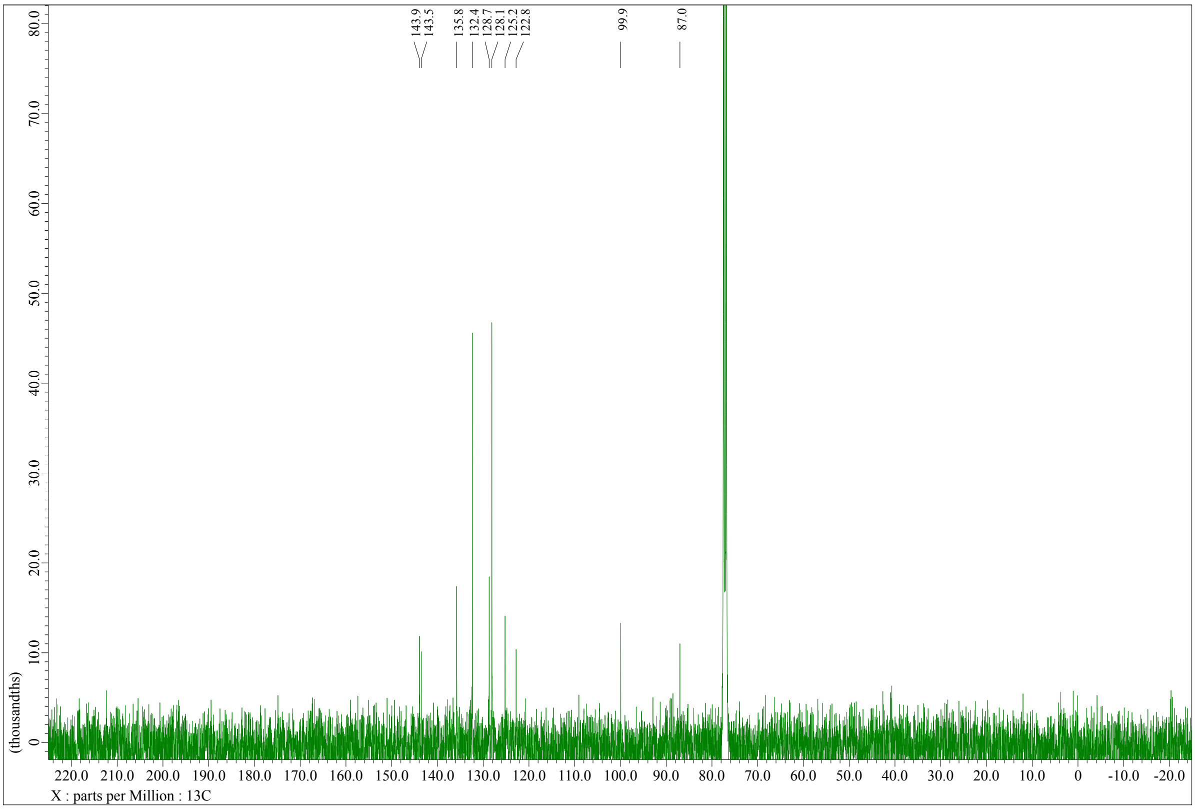

101 MHz

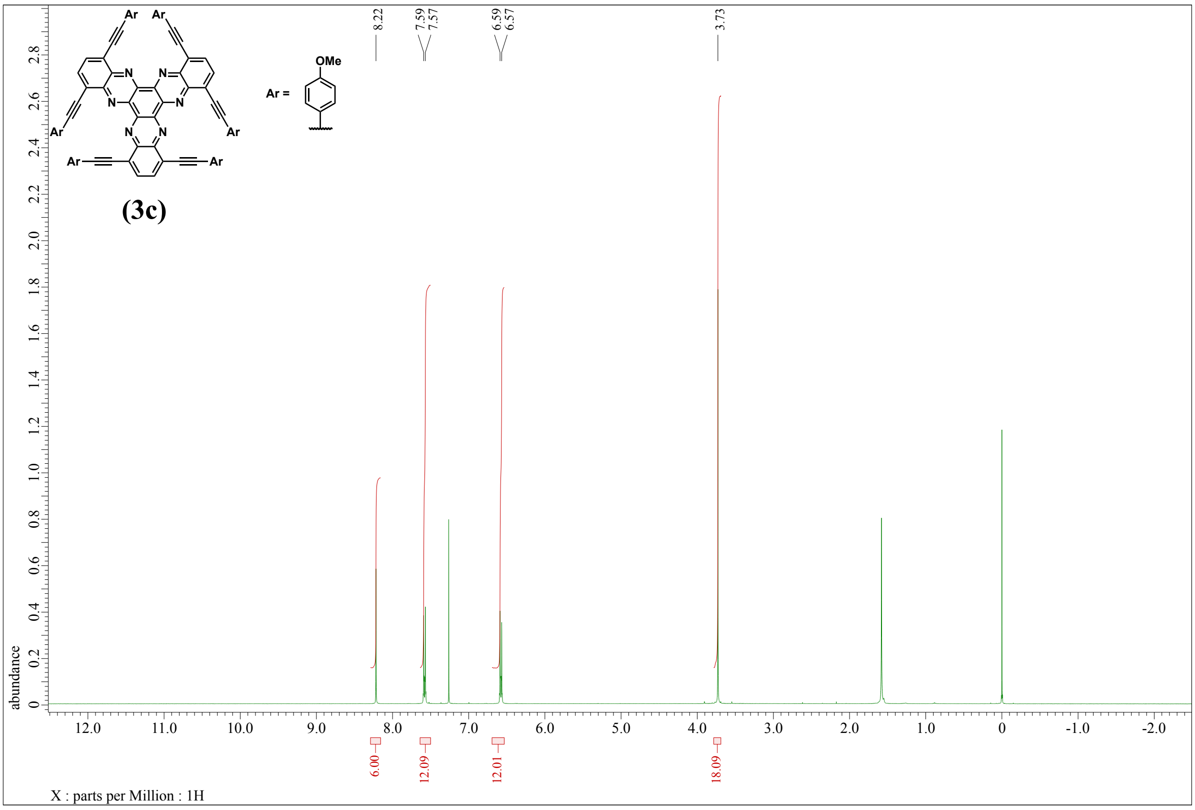

400 MHz

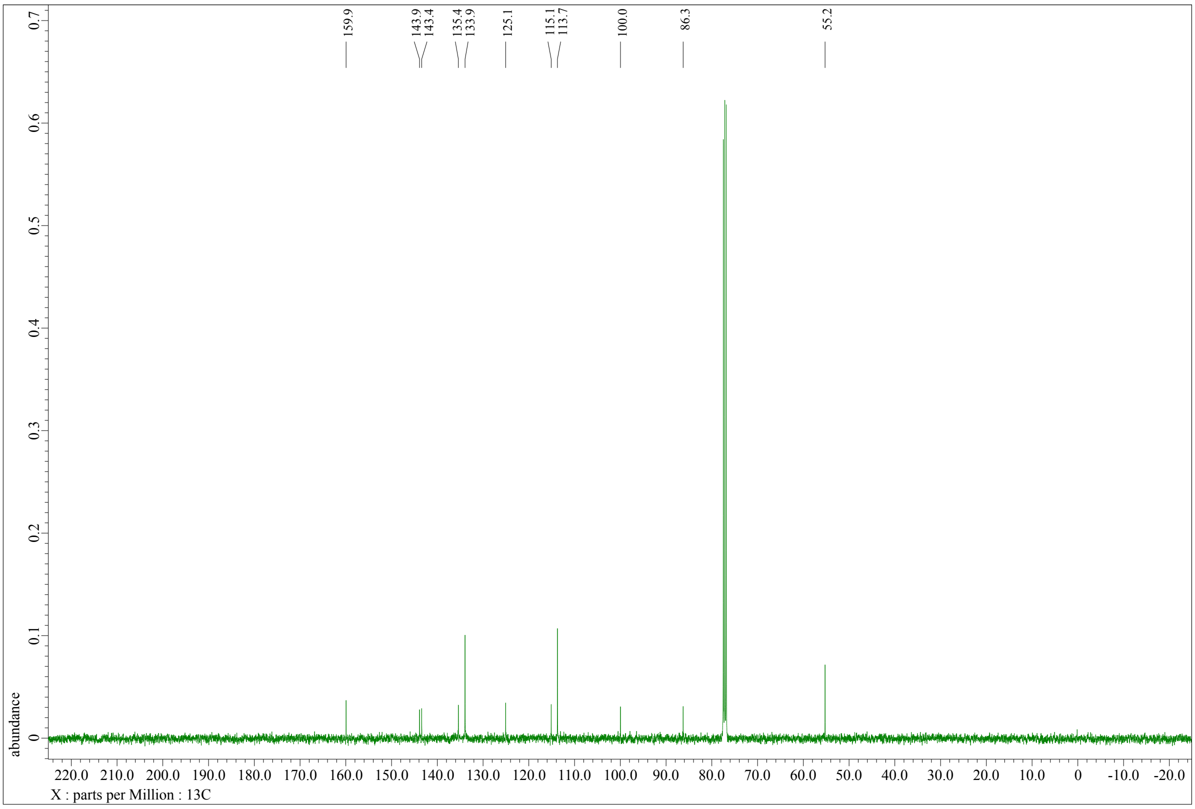

101 MHz

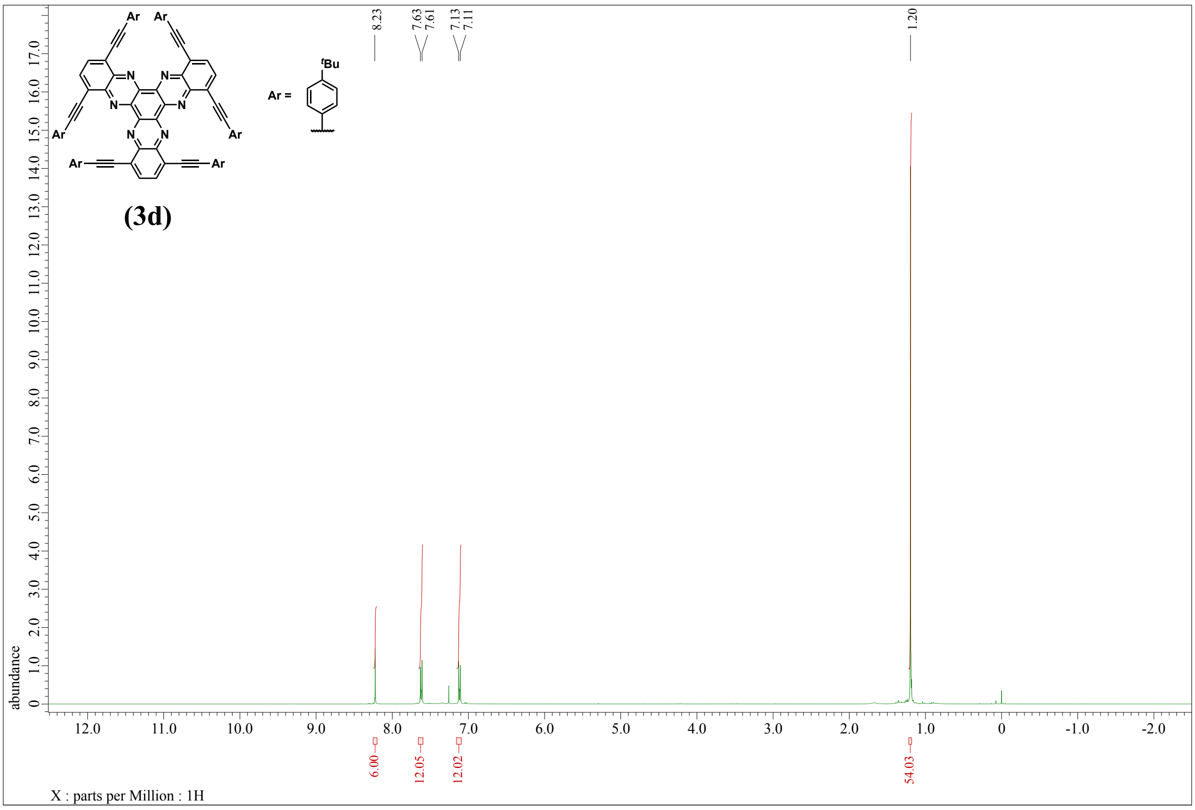

400 MHz

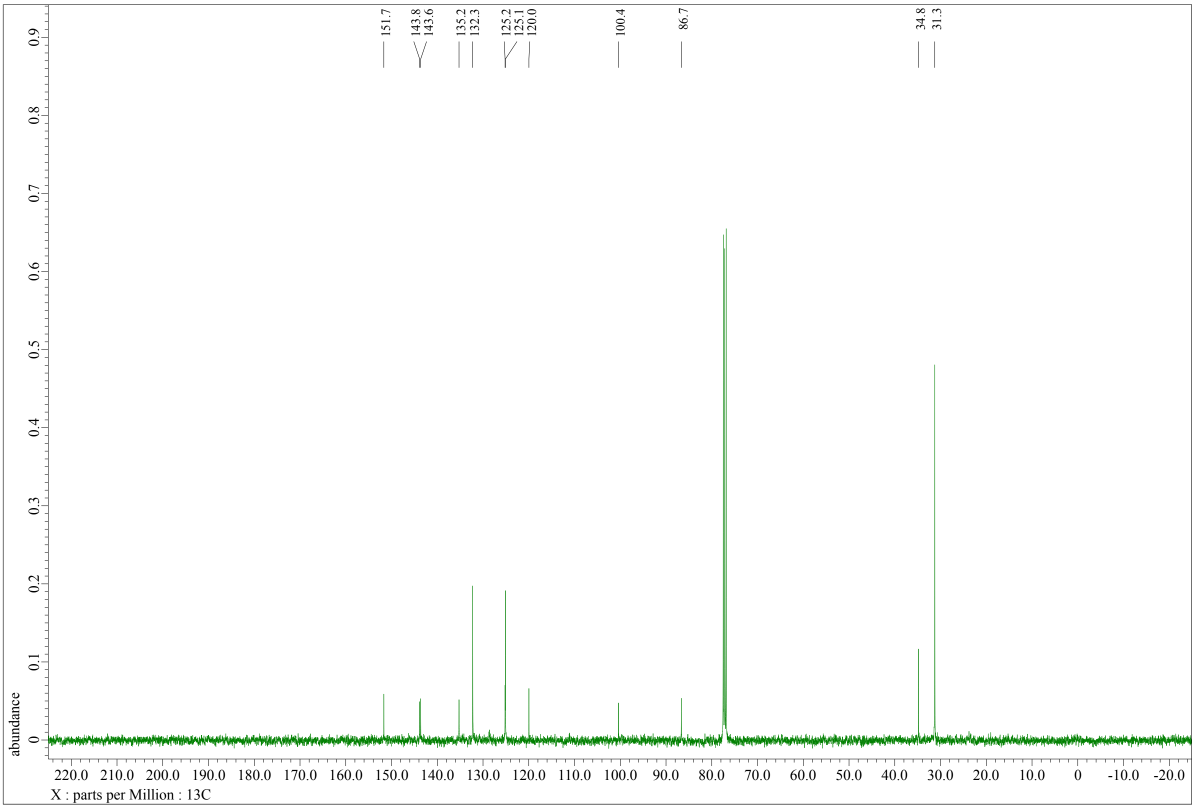

101 MHz

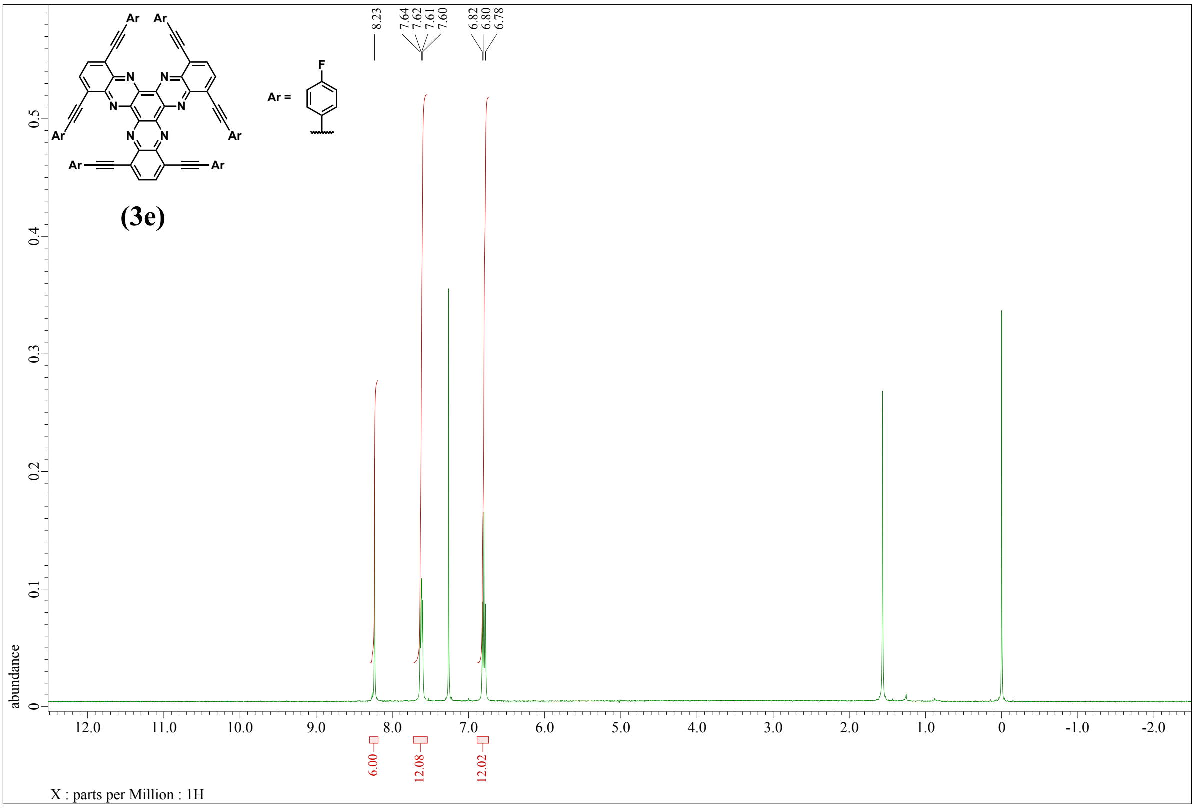

400 MHz

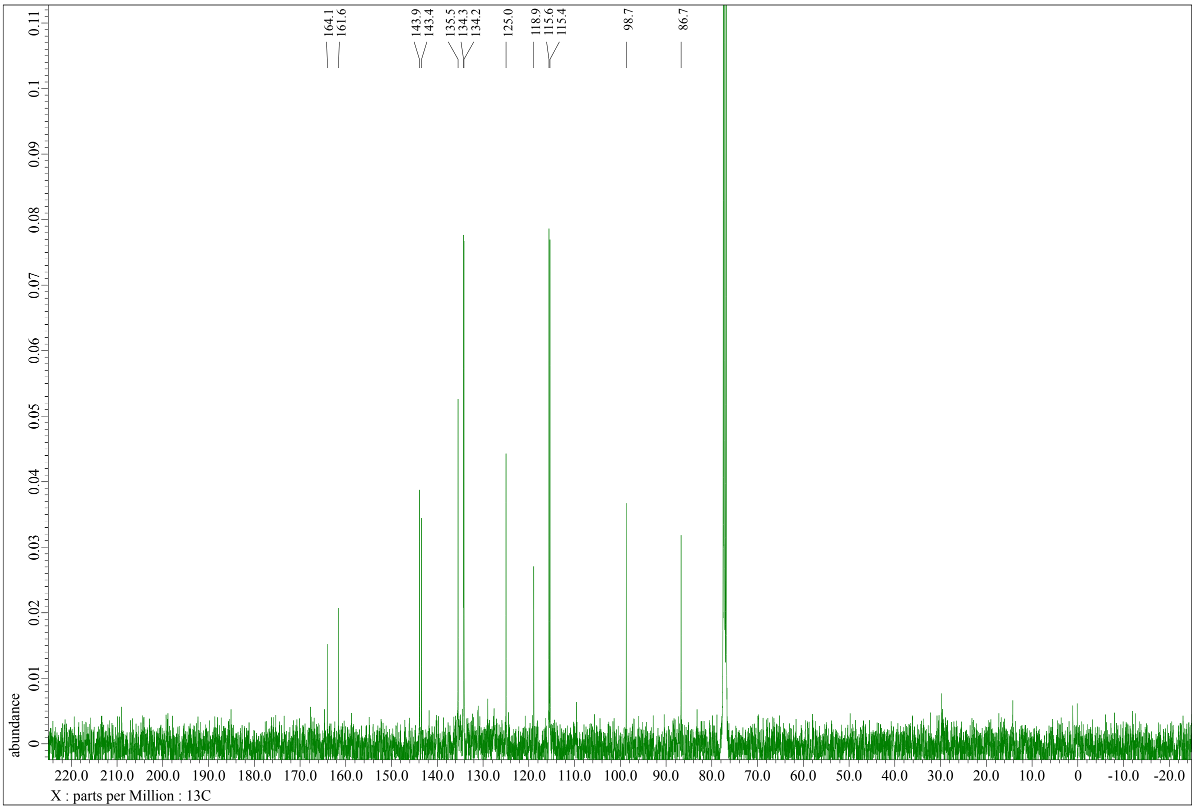

101 MHz

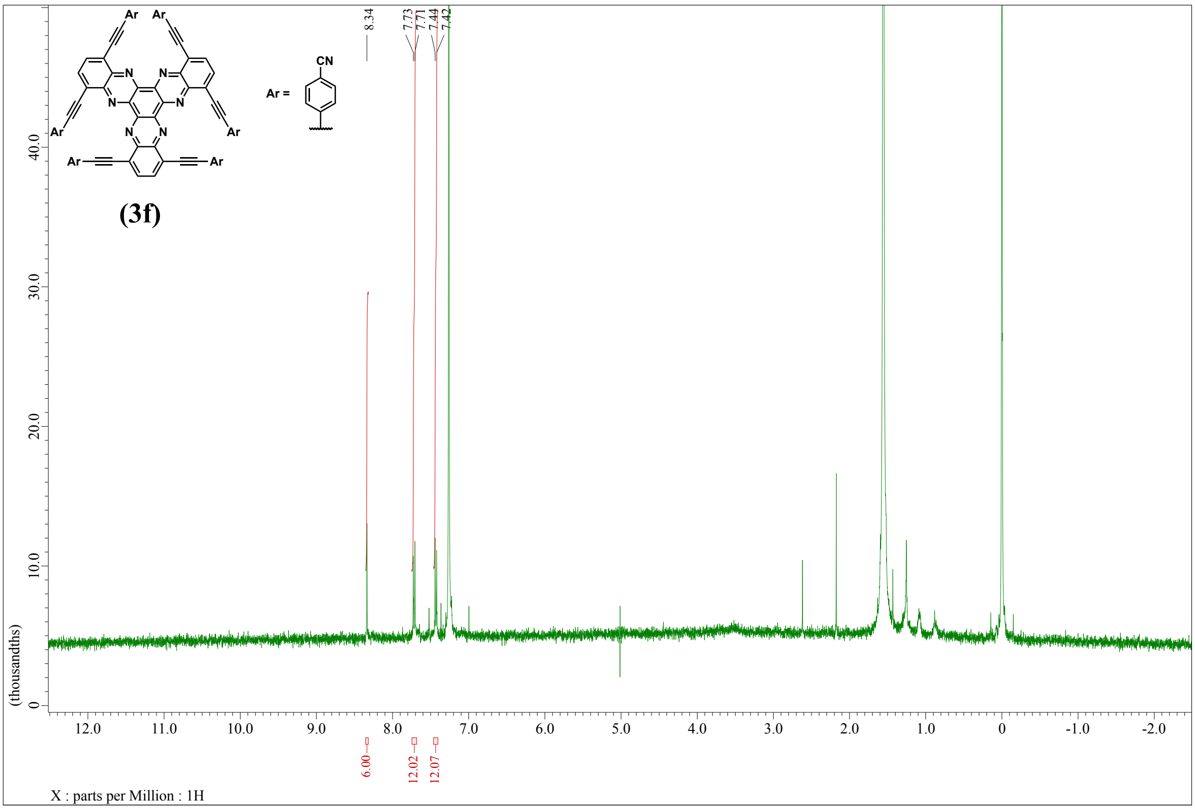

Ar =
CN
(3f)
8.34
7.73
7.71
7.44
7.42
40.0
30.0
20.0
10.0
0
(thousandths)
12.0
11.0
10.0
9.0
8.0
7.0
6.0
5.0
4.0
3.0
2.0
1.0
0
-1.0
-2.0
6.00
12.02
12.07
X : parts per Million : 1H

400 MHz

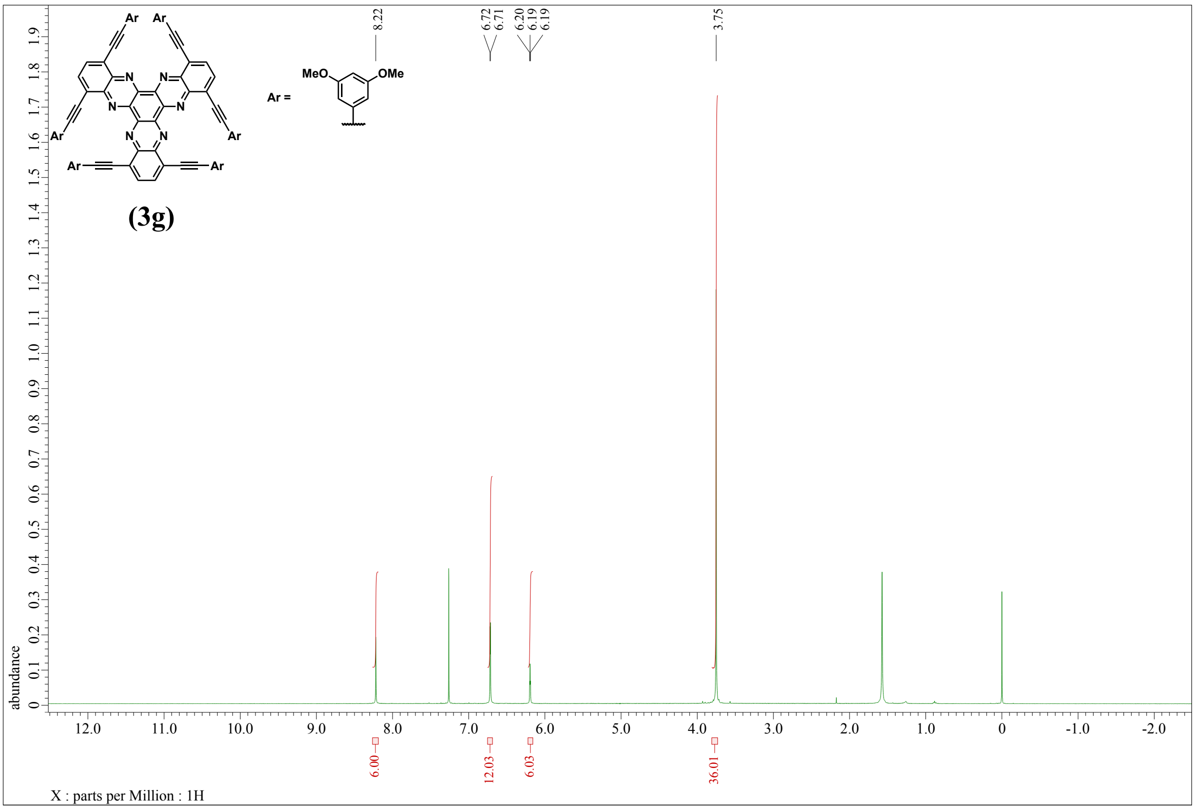

400 MHz

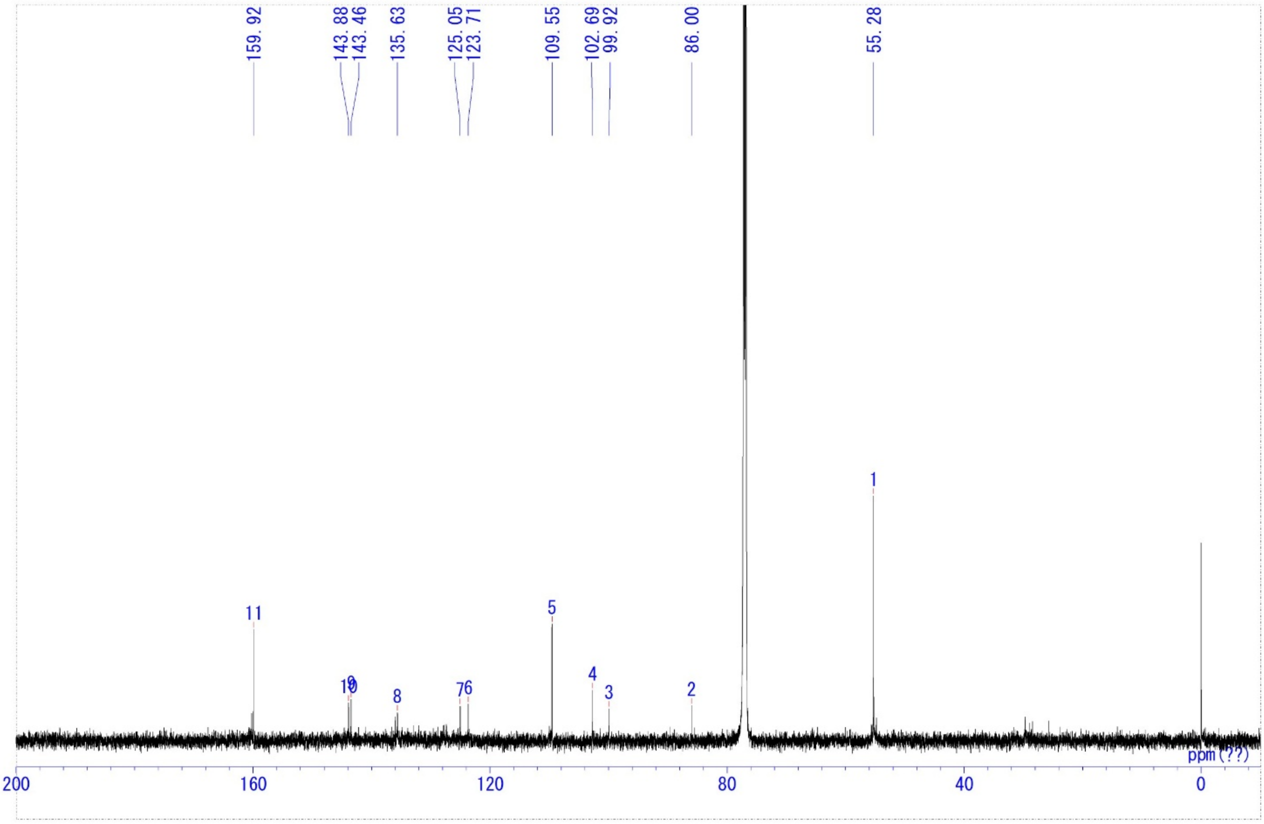

150 MHz

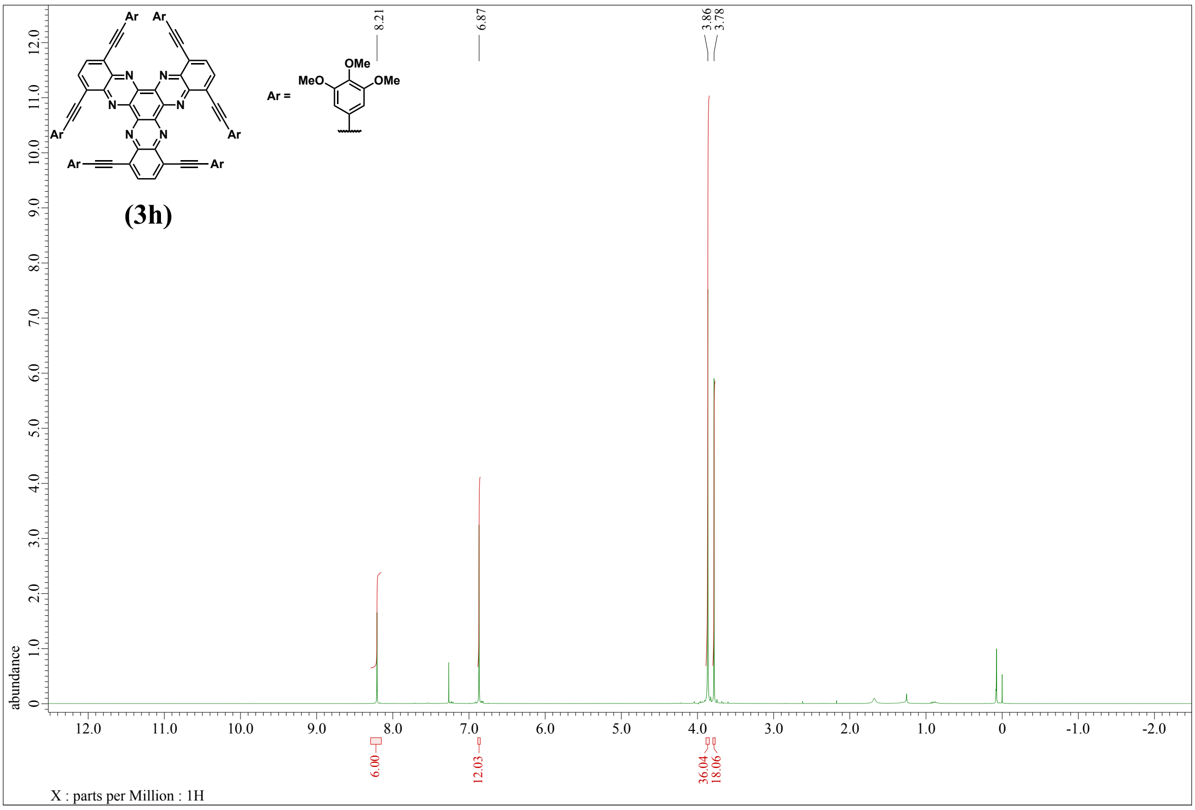

400 MHz

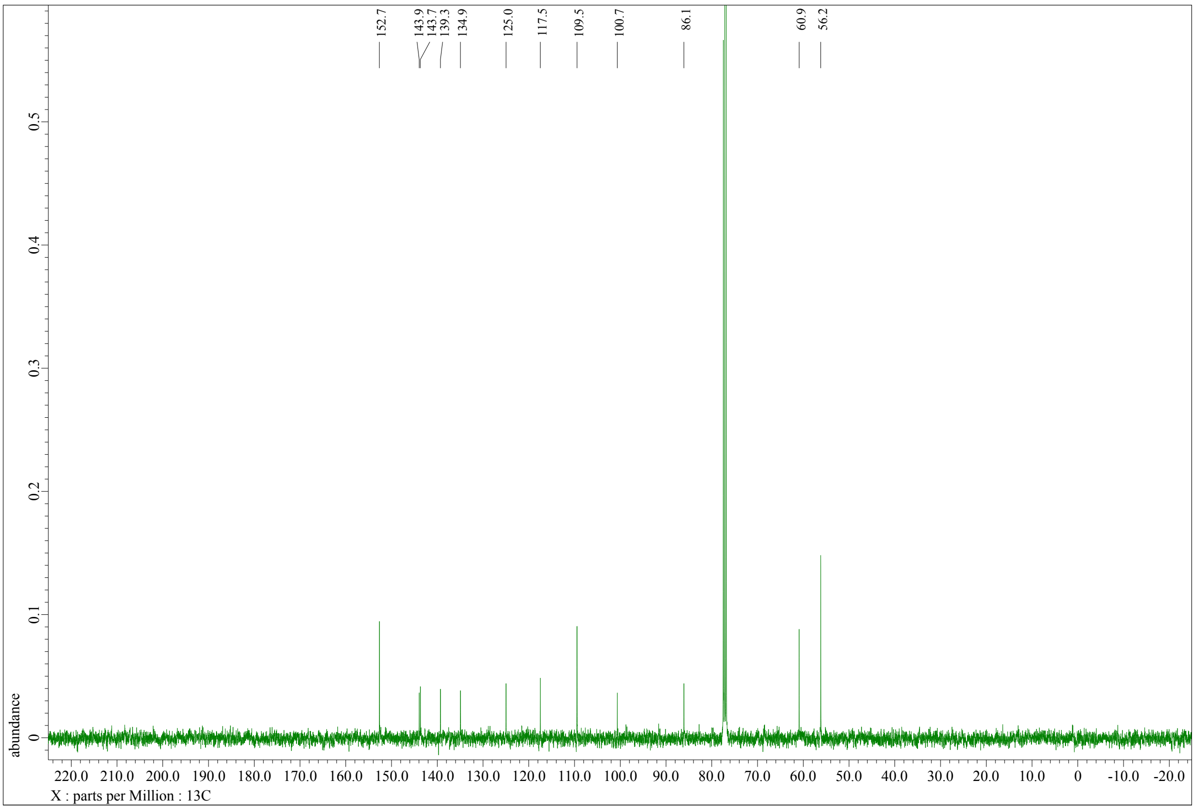

101 MHz

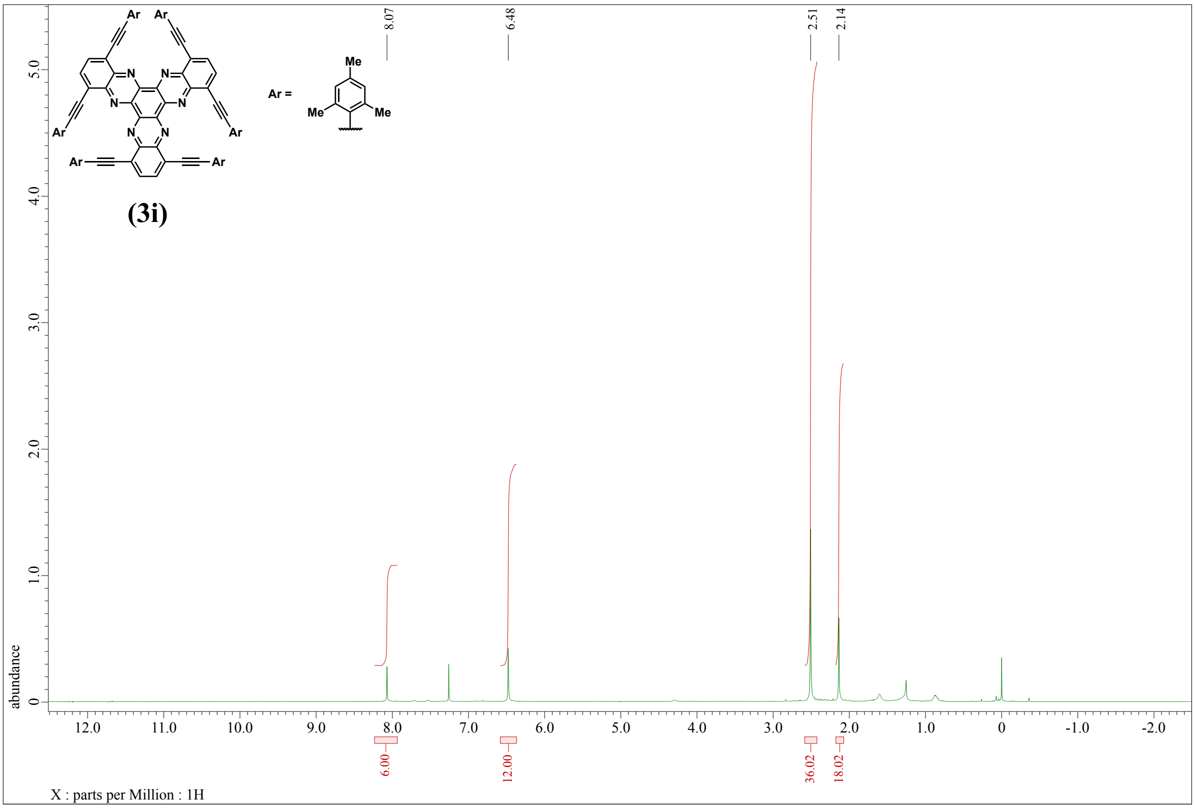

400 MHz

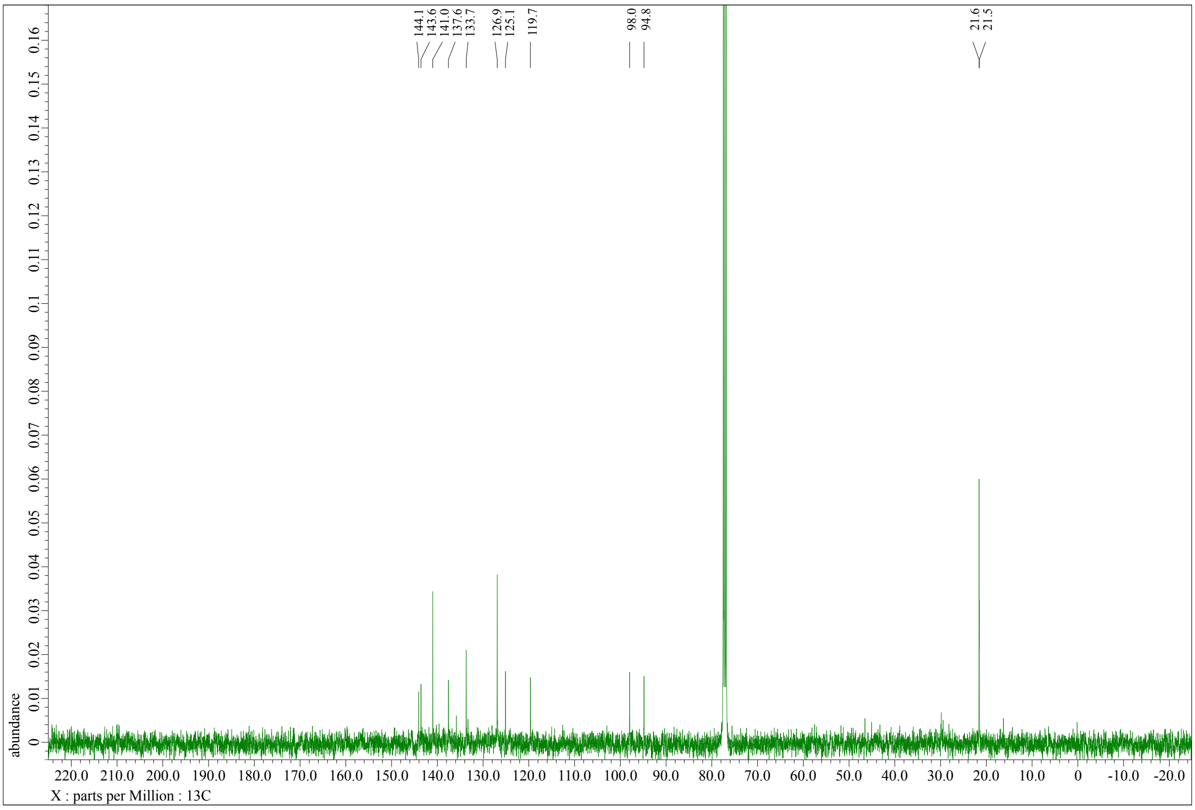

101 MHz

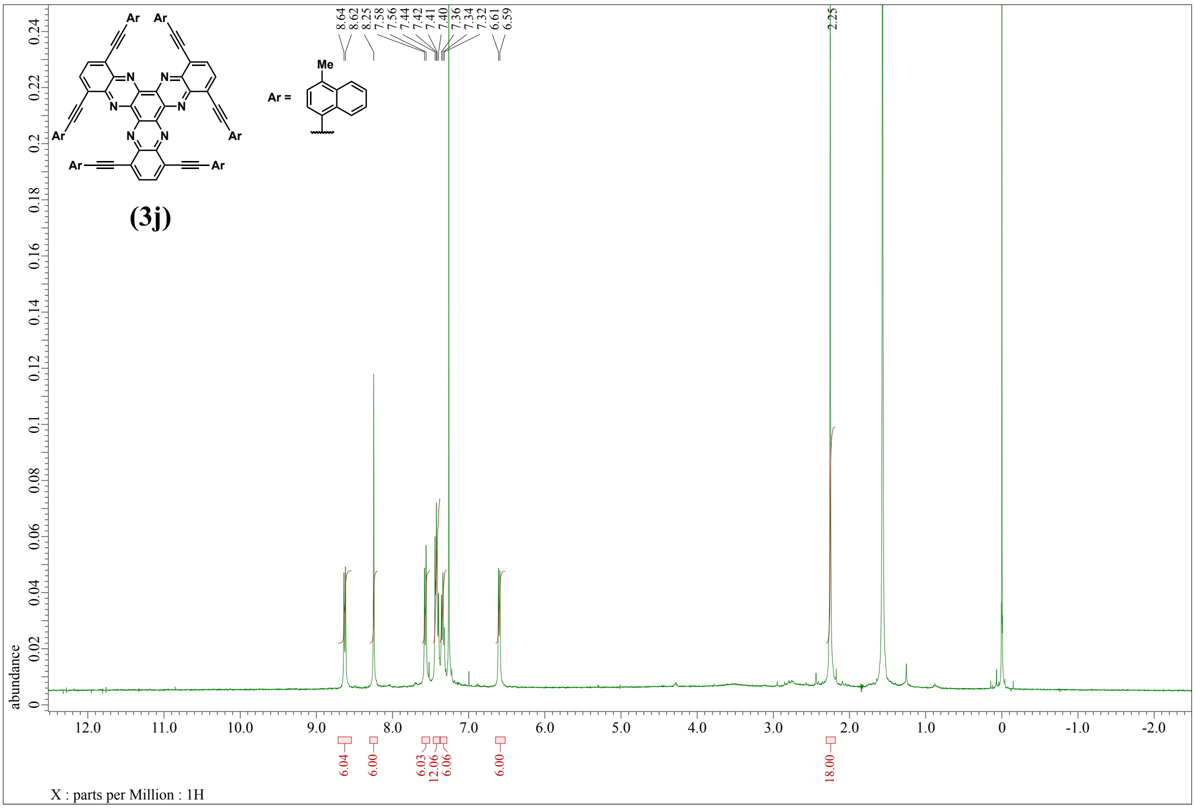

400 MHz

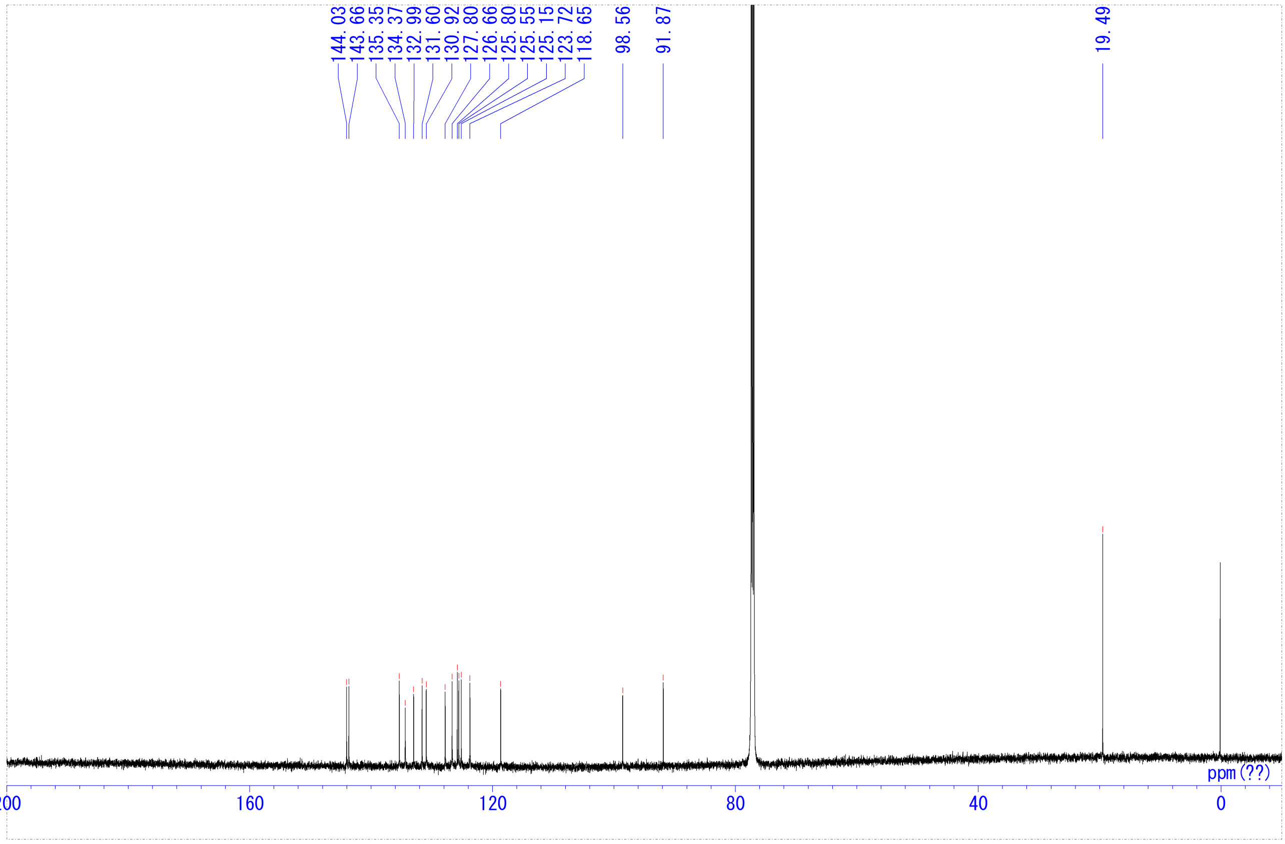

150 MHz

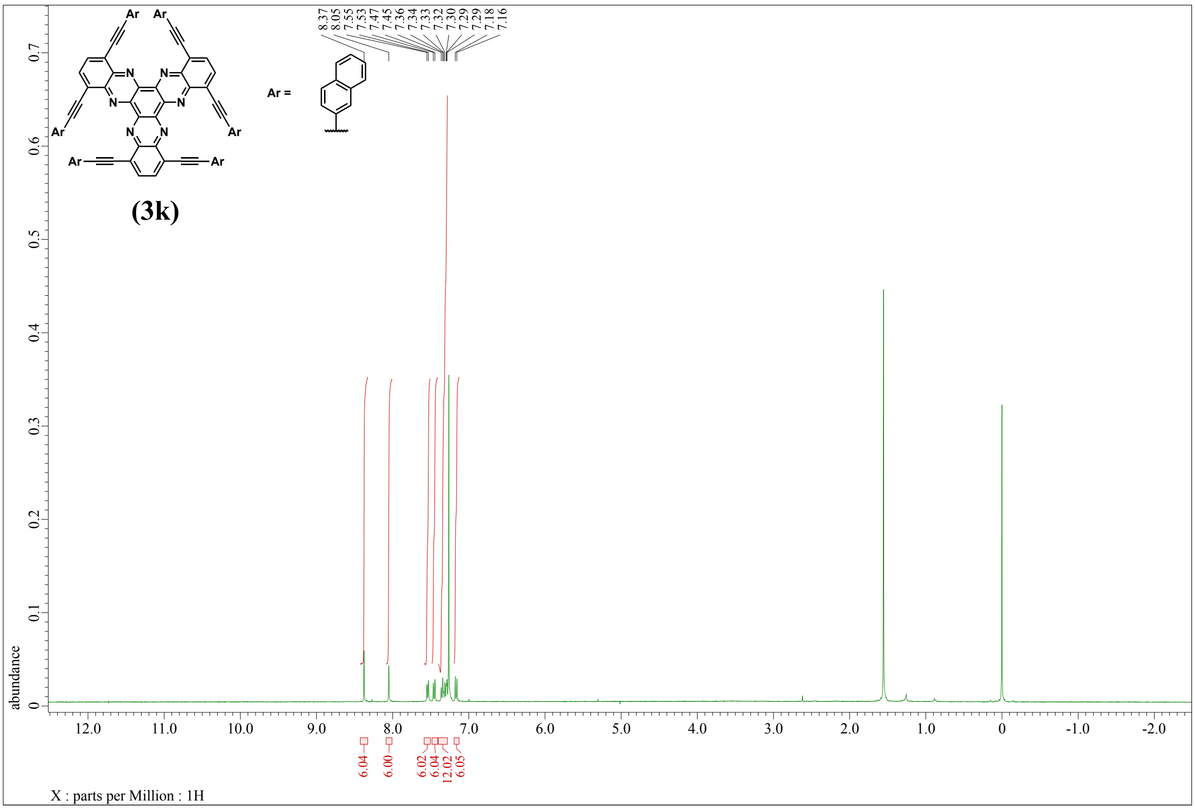

400 MHz

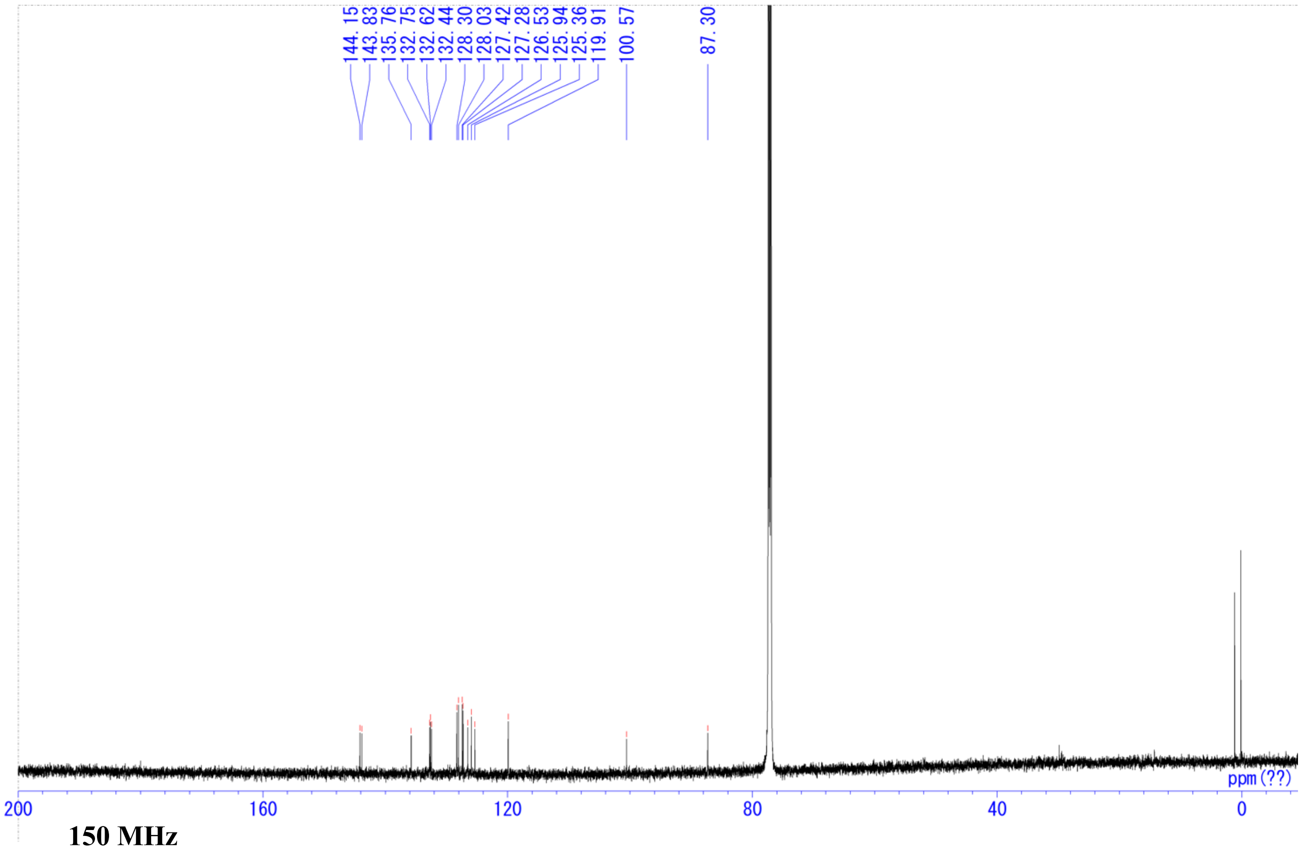

150 MHz

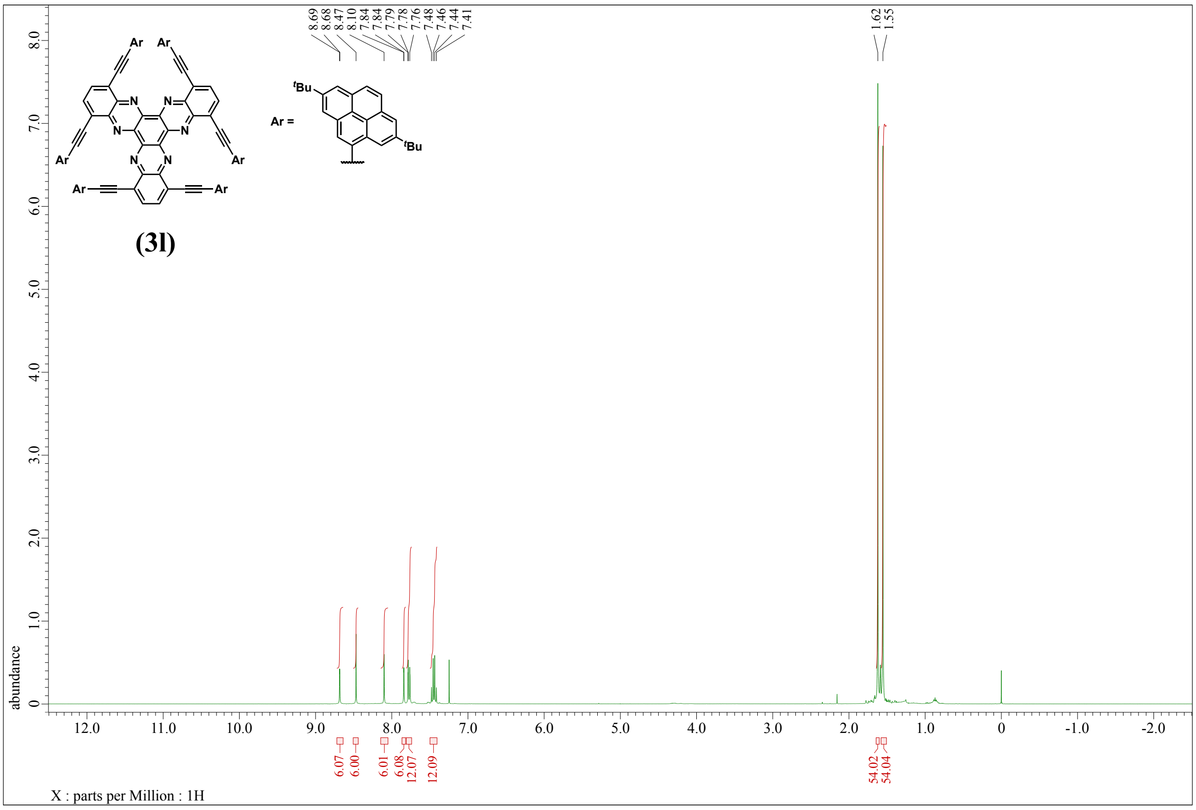

400 MHz

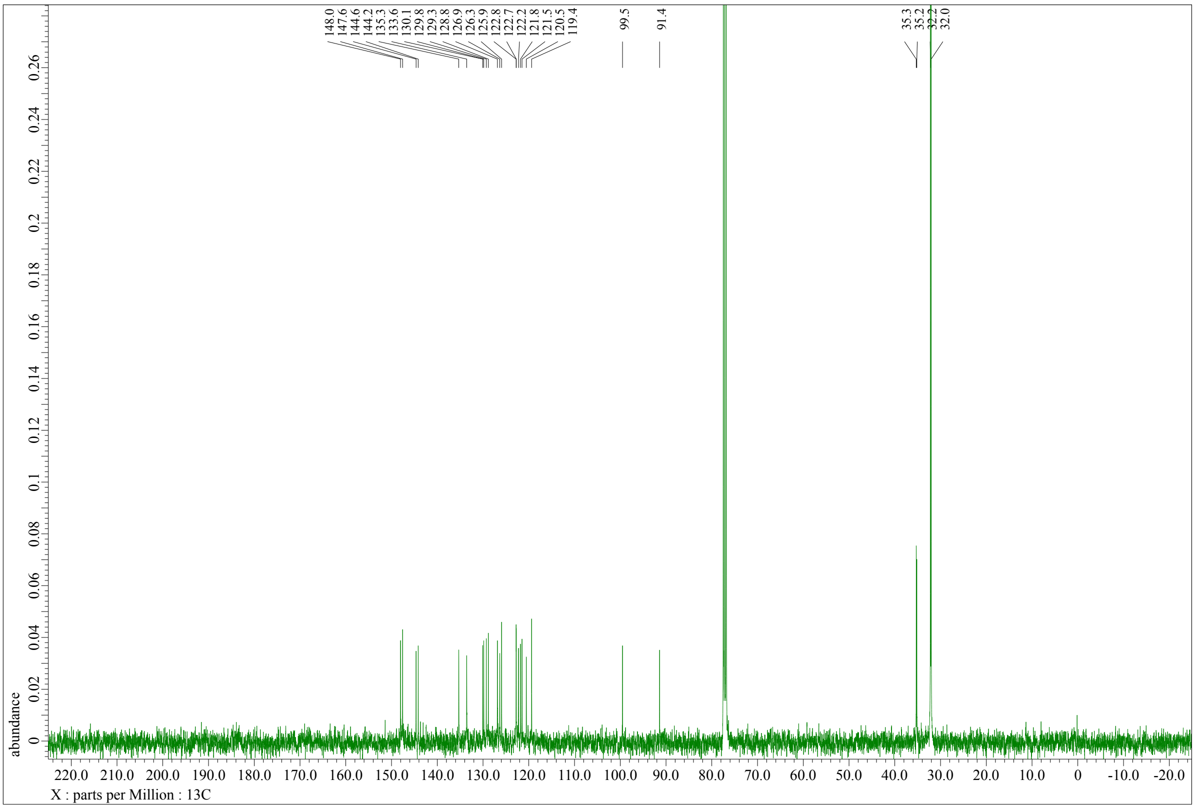

101 MHz

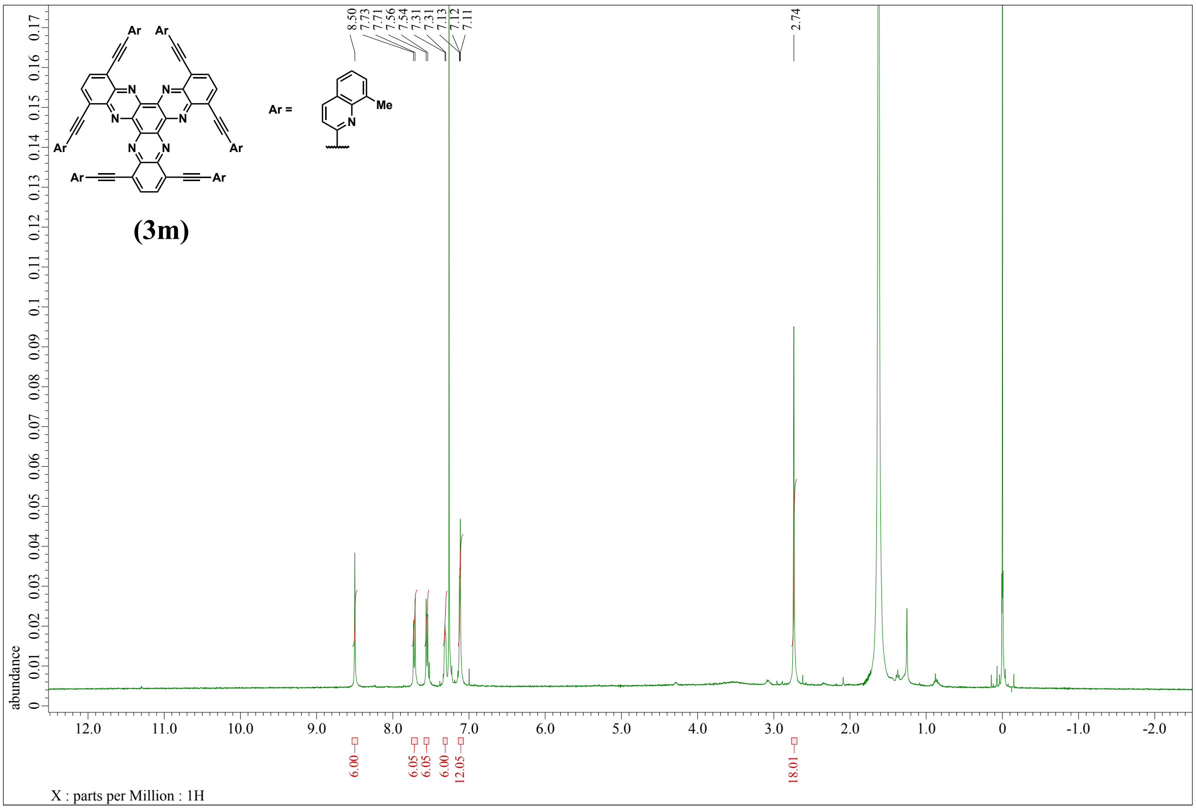

400 MHz

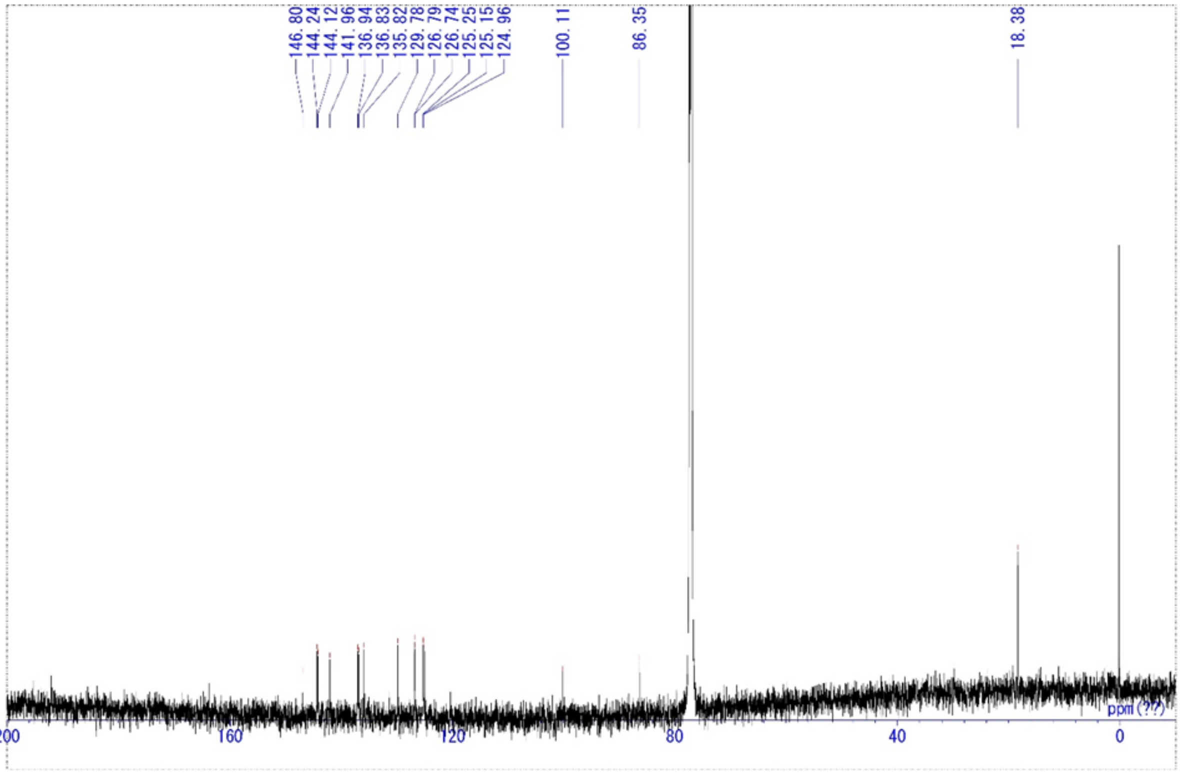

150 MHz

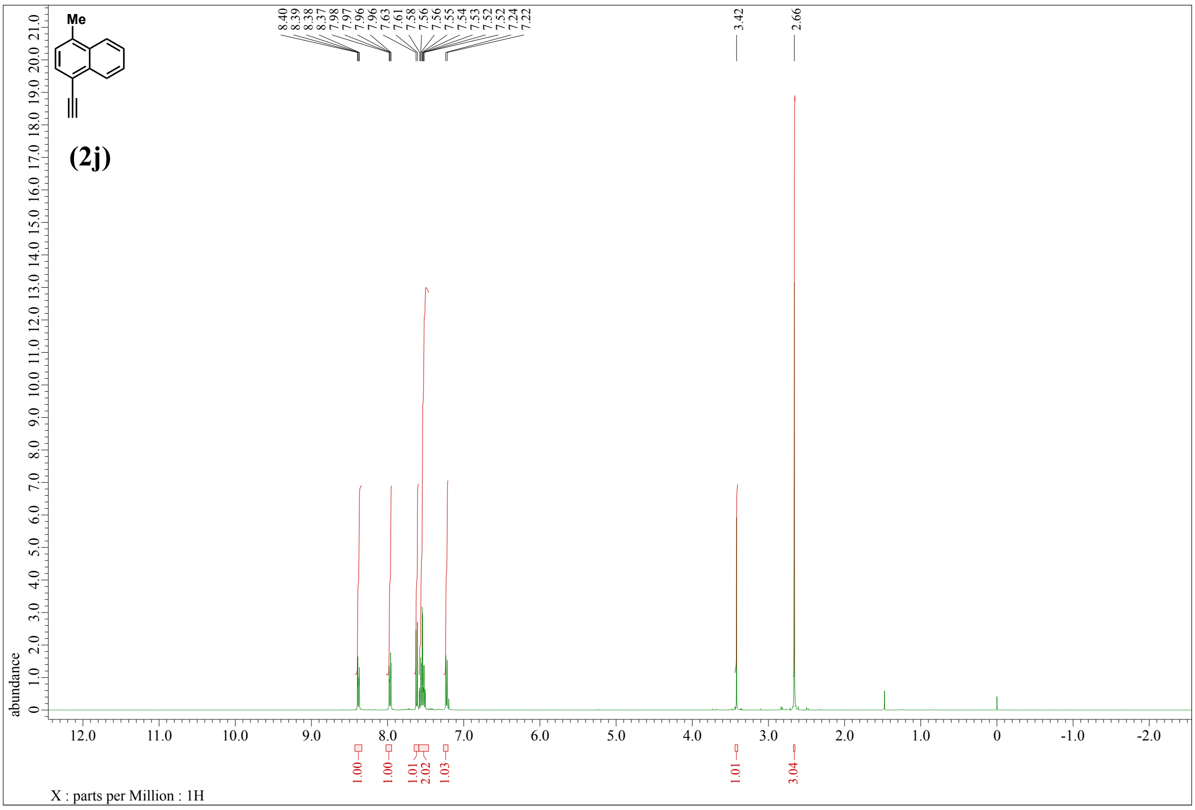

400 MHz

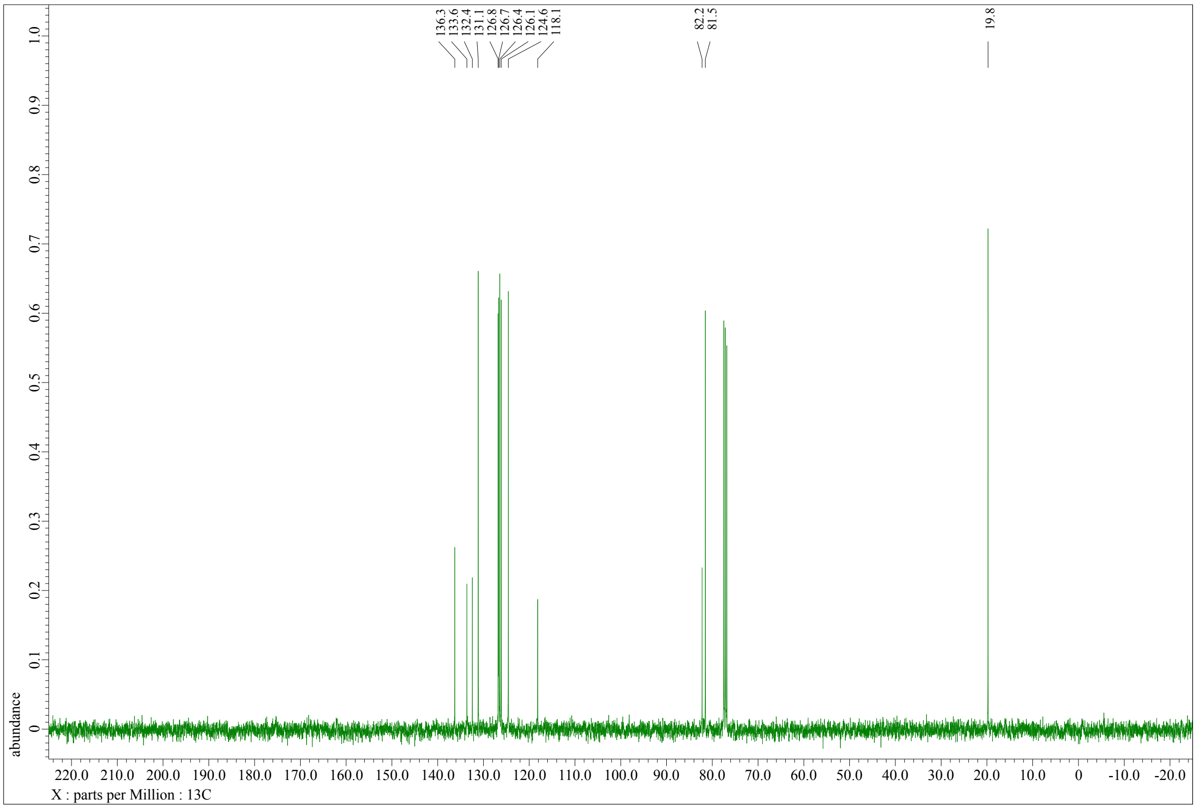

101 MHz

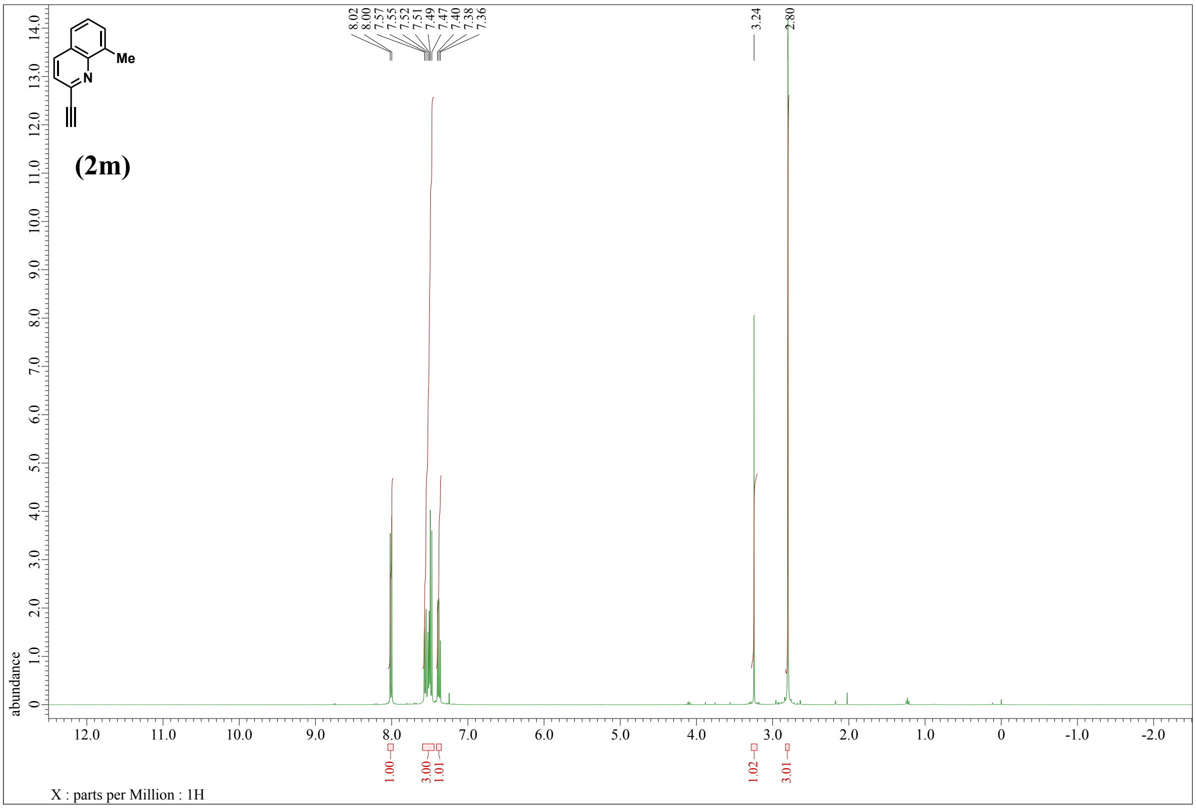

400 MHz

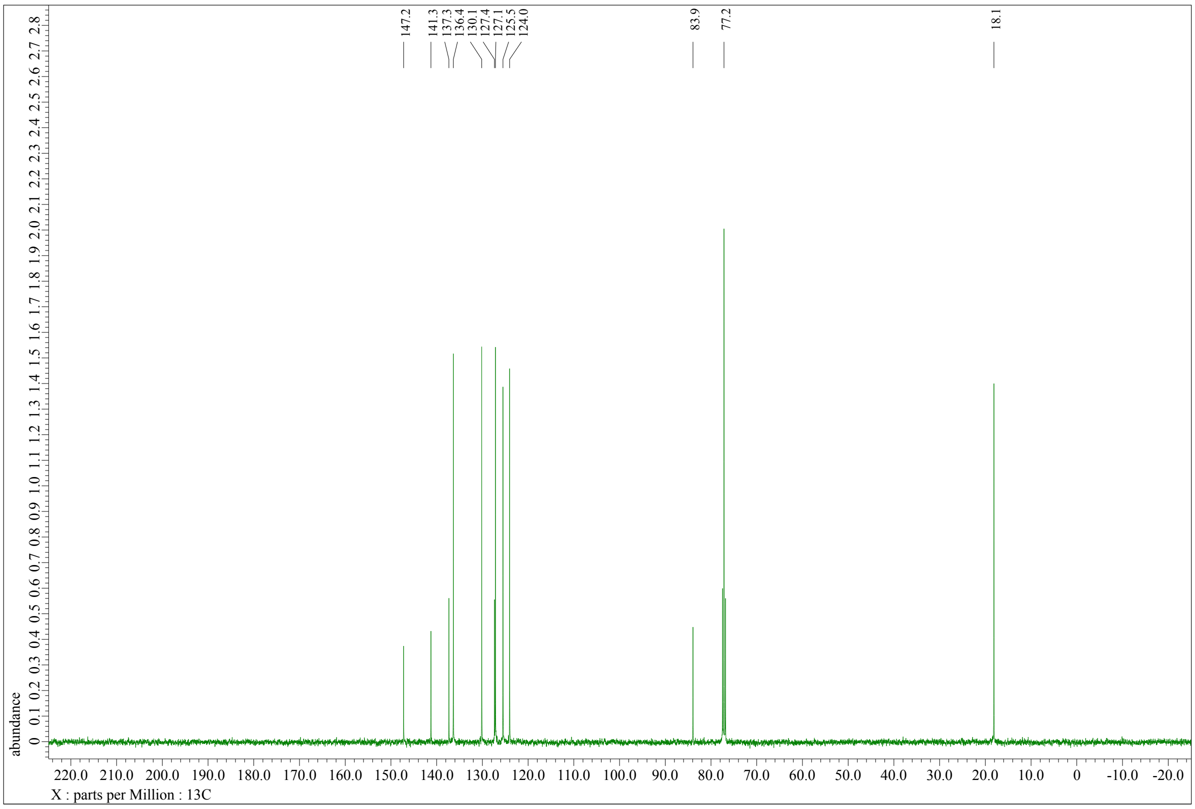

101 MHz

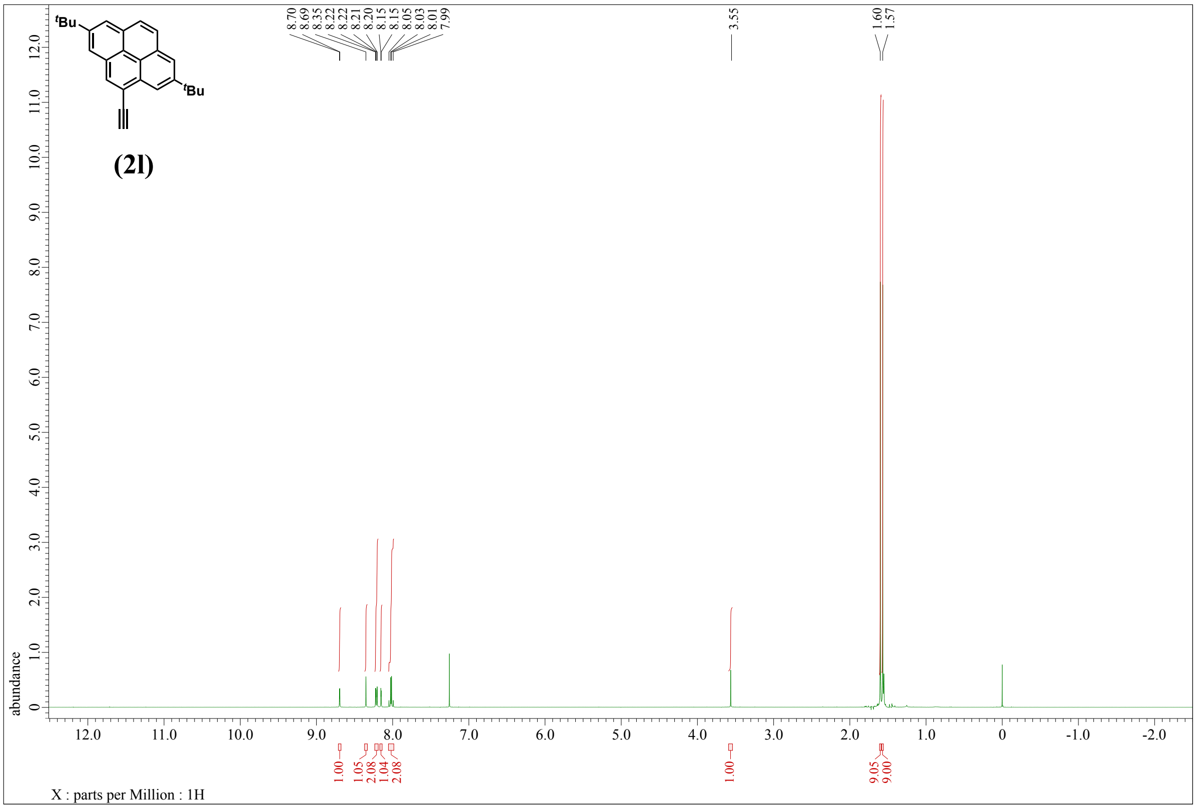

400 MHz

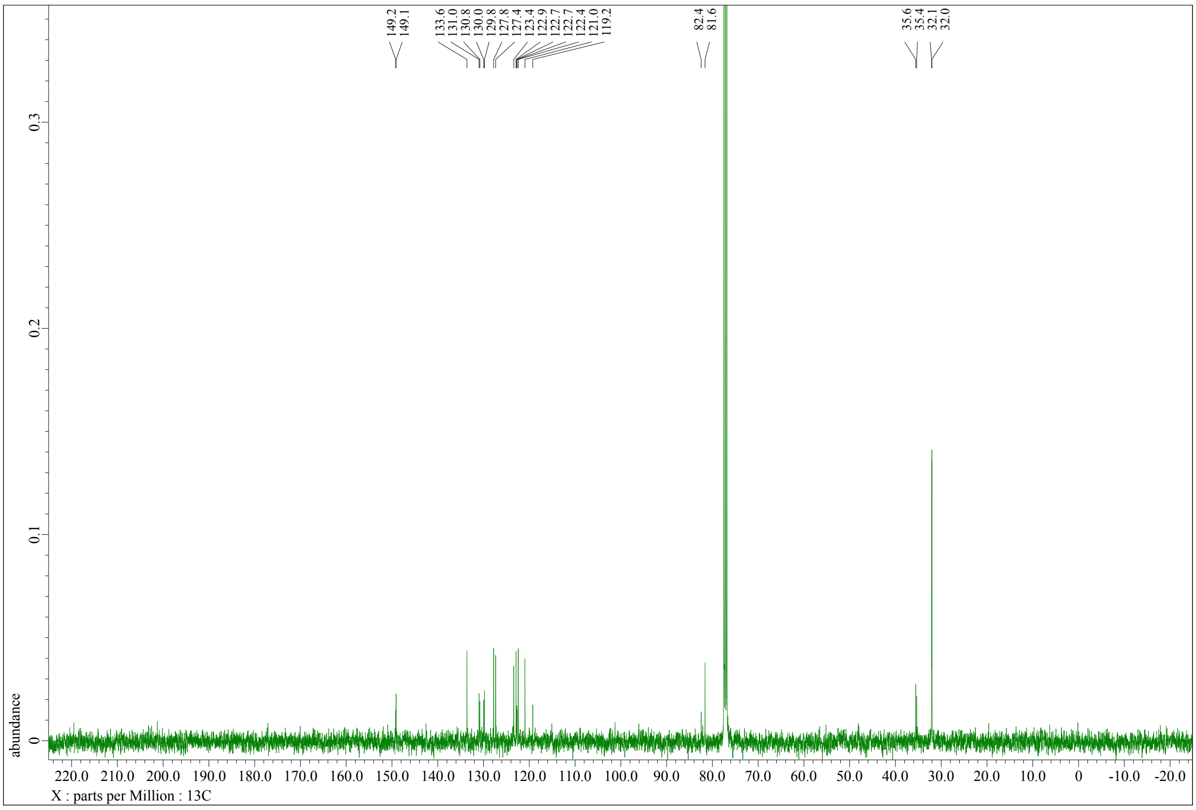

101 MHz
